# A Dominant Mutation in *mediator of paramutation2*, One of Three Second-Largest Subunits of a Plant-Specific RNA Polymerase, Disrupts Multiple siRNA Silencing Processes

**DOI:** 10.1371/journal.pgen.1000725

**Published:** 2009-11-20

**Authors:** Lyudmila Sidorenko, Jane E. Dorweiler, A. Mark Cigan, Mario Arteaga-Vazquez, Meenal Vyas, Jerry Kermicle, Diane Jurcin, Jan Brzeski, Yu Cai, Vicki L. Chandler

**Affiliations:** 1 Department of Plant Sciences, University of Arizona, Tucson, Arizona, United States of America; 2 Department of Biological Sciences, Marquette University, Milwaukee, Wisconsin, United States of America; 3 Pioneer Hi-Bred International, Johnston, Iowa, United States of America; 4 Genetics Department, University of Wisconsin, Madison, Wisconsin, United States of America; The University of North Carolina at Chapel Hill, United States of America

## Abstract

Paramutation involves homologous sequence communication that leads to meiotically heritable transcriptional silencing. We demonstrate that *mop2* (*mediator of paramutation2*), which alters paramutation at multiple loci, encodes a gene similar to Arabidopsis NRPD2/E2, the second-largest subunit of plant-specific RNA polymerases IV and V. In Arabidopsis, Pol-IV and Pol-V play major roles in RNA–mediated silencing and a single second-largest subunit is shared between Pol-IV and Pol-V. Maize encodes three second-largest subunit genes: all three genes potentially encode full length proteins with highly conserved polymerase domains, and each are expressed in multiple overlapping tissues. The isolation of a recessive paramutation mutation in *mop2* from a forward genetic screen suggests limited or no functional redundancy of these three genes. Potential alternative Pol-IV/Pol-V–like complexes could provide maize with a greater diversification of RNA–mediated transcriptional silencing machinery relative to Arabidopsis. *Mop2-1* disrupts paramutation at multiple loci when heterozygous, whereas previously silenced alleles are only up-regulated when *Mop2-1* is homozygous. The dramatic reduction in *b1* tandem repeat siRNAs, but no disruption of silencing in *Mop2-1* heterozygotes, suggests the major role for tandem repeat siRNAs is not to maintain silencing. Instead, we hypothesize the tandem repeat siRNAs mediate the establishment of the heritable silent state—a process fully disrupted in *Mop2-1* heterozygotes. The dominant *Mop2-1* mutation, which has a single nucleotide change in a domain highly conserved among all polymerases (*E. coli* to eukaryotes), disrupts both siRNA biogenesis (Pol-IV–like) and potentially processes downstream (Pol-V–like). These results suggest either the wild-type protein is a subunit in both complexes or the dominant mutant protein disrupts both complexes. Dominant mutations in the same domain in *E. coli* RNA polymerase suggest a model for *Mop2-1* dominance: complexes containing *Mop2-1* subunits are non-functional and compete with wild-type complexes.

## Author Summary

How an individual's genes are activated or silenced is an essential question impacting all fields of biology. Usually gene expression patterns, i.e., which genes are on and which are off in different tissues and during development, are highly reproducible; and those patterns are efficiently reset in the next generation of progeny. Paramutation represents an exception to these genetic rules, in that for certain genes the silencing that is established in an individual is efficiently transmitted to their progeny. Importantly, in these subsequent generations, the silenced gene continues to silence active versions of that gene. Prior work has demonstrated that these heritable gene expression changes are not accompanied by changes in DNA sequence: they are epigenetic. Understanding mechanisms for heritable changes in gene expression has major implications for researchers studying complex traits, including diseases. In this manuscript we demonstrate that a subunit of a RNA polymerase is required for paramutation in maize and other gene silencing processes that also involve RNA–mediated chromatin changes. We show that the multiple, closely related, plant-specific RNA polymerases mediating gene silencing have diverged functions in maize. Results from our experiments suggest testable models for the role of these polymerases in multiple gene-silencing processes.

## Introduction

Paramutation, an interaction between specific alleles that leads to a heritable change of expression of one allele, was first described for the maize *red1* (*r1*) gene [1]. Subsequently three more regulatory genes of the flavonoid biosynthetic pathway, *b1 (Booster1)*, *pl1 (plant color1)*, and *p1* (*pericarp color1*) [2]–[4], and a gene involved in phytic acid biosynthesis [5] were shown to undergo paramutation in maize. Paramutation-like phenomena have also been reported in other plants, fungi, and animals [for a review, see [6]–[8]].

Paramutation terminology defines alleles that induce silencing as paramutagenic and alleles that become silenced as paramutable. Once silenced (paramutated), alleles are designated with an apostrophe to signify their paramutant state. In addition to becoming heritably silenced, paramutant alleles also acquire the ability to silence naïve paramutable alleles. Paramutant and paramutable states often have different stabilities, which can potentially be reversible depending on the locus [for a review, see [7],[9],[10]]. Most alleles of a locus do not participate in paramutation.

Key sequences mediating paramutation have been identified for two systems, *b1* [11],[12] and *p1* [4],[13]. Recombination mapping between alleles that do and do not participate in *b1* paramutation defined a specific sequence that when tandemly repeated is absolutely required for paramutation [11],[12]. Characterization of these repeats revealed that the paramutable and paramutagenic alleles have identical DNA sequences and numbers of repeats, but differ in their chromatin structure demonstrating that paramutation is epigenetic and associated with changes in chromatin [11]. Transgenic approaches were used to identify sequences within *p1* sufficient to mediate paramutation. These sequences lie within a direct repeat flanking the *p1* alleles that participate in paramutation [4],[13]. At the *r1* locus, paramutagenic alleles contain direct and inverted repeats and the strength of paramutation correlates with repeat number [14]; paramutable alleles have inverted repeats [15]–[18]. Thus, while there are no sequence similarities between the regions that mediate paramutation at these distinct loci, a common theme is the presence of direct or inverted repeat sequences.

Mutations that alter paramutation have been isolated using screens with either the *b1* or *pl1* paramutation systems [19]–[21]. Several of the genes identified in these paramutation screens have been cloned and to date all share homology with genes in Arabidopsis that mediate RNAi transcriptional silencing of transgenes or endogenous genes. The first cloned gene required for paramutation was *mediator of paramutation 1* (*mop1*), which encodes a RNA dependent RNA polymerase most similar to Arabidopsis *RDR2* [22] that mediates heterochromatic silencing of repeats through 24 nt siRNAs [23]. In addition to preventing paramutation at multiple loci and increasing the transcription of paramutated alleles [20], *mop1* mutations also reactivate *Mutator* transposons [24]–[26] and transcriptionally silenced transgenes [27]. The second gene cloned, *required to maintain repression1* (*rmr1*), encodes a SNF2-like ATPase [28], a factor similar to, but distinct from Arabidopsis *DRD1* (*Defective in RNA Directed DNA methylation1*) involved in RNAi-mediated transcriptional silencing [29] and *CLSY1* (*CLASSY1*) involved in RNA signal spreading [30]. The *rmr1* mutation increases the expression of previously silenced *pl1* and *b1* alleles, but does not prevent paramutation at *pl1* [19],[28] or *b1* (V. Chandler, unpublished data), suggesting it is involved in maintaining the silenced epigenetic states. While *rmr1* mutations can also reactivate transcriptionally silenced transgenes, these transgenes are efficiently resilenced upon the introduction of a wild type allele [27], in contrast to *mop1* mutations in which the reactivated transgenes can remain heritably active even when the wild type allele is reintroduced [27]. The third gene cloned, *rmr6*, encodes the largest subunit of the plant specific DNA-dependent RNA polymerase most similar to Arabidopsis *NRPD1* [31], the largest subunit of the Pol-IV complex required for primary siRNA biogenesis [32],[33]. Mutations in *rmr6* cause dramatic developmental phenotypes and prevent paramutation at *pl1, b1*, and *r1* as well as relieve silencing of paramutant alleles [21]. The genes cloned to date, when mutated, show a dramatic reduction in 24 nt siRNAs normally associated with heterochromatic silencing of repeated sequences [31],[34].

In Arabidopsis the related RNAi heterochromatic silencing pathway is often referred to as RdDM (RNA directed DNA Methylation); the transcriptional silencing requires small RNA biogenesis and targets homologous promoters with DNA methylation and repressive histone modifications [35]–[37]. This pathway in Arabidopsis mediates transcriptional silencing by transgenes in which inverted repeats of promoters are transcribed to generate dsRNA (referred to a pIR transgenes) [38]. The dsRNA is processed to 24 nt siRNAs, leading to DNA and chromatin modifications and silencing of any endogenous gene or transgene sharing homologous promoter sequences [36]–[39]. This pathway also mediates *de novo* DNA methylation and silencing of several endogenous genes associated with tandem repeats [40].

The current model for the Arabidopsis RNAi heterochromatic silencing pathway involves the genes identified in maize discussed above as well as other factors. Pol-IV is required for siRNA biogenesis and is thought to mediate the synthesis of non-coding RNAs at multiple repetitive endogenous loci using either double-stranded DNA, or single- or double-stranded RNA as a template [for a review, see [37]]. The resulting single stranded RNA is postulated to be used by the RNA dependent RNA polymerase, encoded by *RDR2* [23], to generate double stranded RNA molecules that are then diced into double stranded 24 nt siRNAs by an RNAseIII-like endonuclease, encoded by *Dicer-like 3* (*DCL3*) [23]. Transgenes or endogenous genes that can produce dsRNA via strong Pol-II promoters, such as pIR transgenes, do not require Pol-IV or RDR2 for silencing [41]. Another plant-specific Pol-II related polymerase complex, Pol-V [42], associates with target DNA with the help of the SNF2-like ATP-dependent chromatin remodeler, encoded by *DRD1* (*RNA-directed DNA methylation1*) [43], and a hinge protein, DMS3 [44]. Pol-V produces transcripts regardless of the presence or absence of the 24 nt siRNA signal [45],[46]. Nascent Pol-V RNA transcripts associate with an RNA binding protein KTF1 (KOW domain containing transcription factor 1) [47] and recruit the ARGONAUTE4 (AGO4) protein [48], which is complexed with a guiding strand of complementary 24 nt siRNA. The AGO4 complex then recruits the de-novo DNA methylation enzyme DRM2 (domain rearranged methytrasferase2) [45]–[47],[49], and histone modification factors such as HDA6 (Histone deacetylase6), and a histone methyltransferase, KYP (Kryptonite), to establish and reinforce silencing at target loci [50],[51].

The requirements of a RDR2-like RNA dependent RNA Polymerase encoded by *mop1* [22], a NRPD1-like large subunit of Pol-IV encoded by *rmr6* [31], and the SNF2-like factor encoded by *rmr1* [28], coupled with the requirements for transcribed tandem repeats to mediate *b1* paramutation [11],[22], loss of siRNAs in several paramutation mutants [31],[34], and associated chromatin differences between paramutagenic and paramutable *b1* and *p1* alleles [4],[52],[53], suggest that paramutation involves a mechanism similar to the RdDM pathway in Arabidopsis. However, paramutation has properties that are distinct from RdDM [8],[40]. Most dramatically, the silencing associated with paramutation is highly heritable after the paramutagenic allele is segregated away and the newly silenced allele itself becomes paramutagenic in subsequent generations. These characteristics do not occur with RdDM in Arabidopsis; in most instances expression of the targeted loci returns to normal after the inducing transgene (or locus) is segregated away. Even in the examples of heritable silencing at the *FWA* locus, the silenced allele is not paramutagenic; it can not silence an active allele as reviewed in [40]. In addition, maize contains significant levels of a new class of 22 nt heterochromatic RNAs [34], suggesting greater complexity with these processes in maize. Clearly, further studies of paramutation in maize are needed to understand how the paramutagenic and paramutable alleles communicate to set up and heritably maintain RNA-mediated transcriptional silencing.

In this report we describe the identification of *mop2* and show that it is required for paramutation at multiple loci. Map-based cloning demonstrated that *mop2* encodes a second largest subunit of plant specific RNA polymerases similar to the NRPD2/NRPE2 subunit shared by Pol-IV and Pol-V in Arabidopsis. Unlike Arabidopsis, which encodes a single functional gene, maize encodes three closely related genes, all of which appear to encode full length proteins and show significant overlapping expression in a variety of tissues. We report on a number of additional gene silencing phenotypes of an EMS-induced dominant mutant allele of *mop2*, *Mop2-1*, which has a single nucleotide change in a domain highly conserved among all polymerases ranging from *E.coli* to higher eukaryotes. Our results suggest that *Mop2-1* is disrupting siRNA biogenesis and may be disrupting chromatin targeting properties of small RNAs in maize, and that its ability to disrupt epigenetic processes varies with dosage. Models for *Mop2-1* dominance and implications of our findings for mechanisms of *b1* paramutation are discussed.

## Results

### Dominant mutation, *mediator of paramutation2-1*, prevents *b1* paramutation and activates previously silenced *B'* alleles

**Figure 1:**
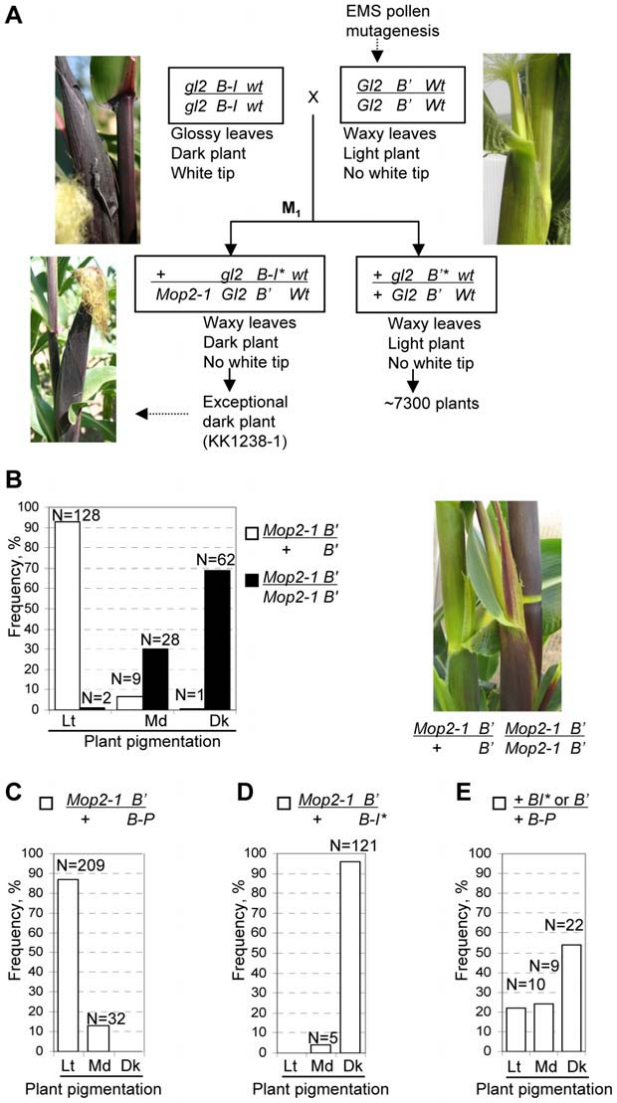
The *Mop2-1* mutation prevents *b1* paramutation and relieves *B'* silencing. (A) The EMS mutagenesis screen used to isolate *Mop2-1*. The stocks carry distinct alleles for two genes that are linked and flank *b1* on chromosome 2S; *glossy2 (gl2)* and w*hite tip* (*wt*). The glossy and white tip phenotypes are only visible in young seedlings and thus are not apparent in the mature plants shown. B'* is used to indicate a *B-I* allele that was paramutated to *B'* in wild type plants. B-I* is used to signify a *B-I* allele exposed to *B'* in the presence of *Mediator of paramutation2 (Mop2-1)*, which prevents paramutation resulting in the *B-I* phenotype. *Mop2-1* is shown linked to *GL2* based on subsequent analysis (Figure S1). (B) Frequencies of plants with different pigmentation levels [Lt (light), Md (medium), and Dk (dark)] in progeny segregating *Mop2-1/Mop2-1* and *Mop2-1/+*. The photo shows that *Mop2-1 B'/+ B'* pigmentation remains light (left plant), indistinguishable from *B'* in wild type. In contrast, homozygous *Mop2-1 B'* plants have increased pigmentation (right plant). For (C) and (D), to test the heritability of the increased *B'* pigmentation that is observed in homozygous *Mop2-1 B'* plants (D) and to generate larger numbers of progeny to examine the penetrance of *Mop2-1/+* on preventing B-I* paramutation (C) dark *Mop2-1 B'* plants were crossed with +/+ tester carrying *B-I/B-P*, (Figure S2). The resulting *Mop2-1 B'/+ B-P* (C) and *Mop2-1 B'/+ B-I* (D) progeny were scored for light (Lt), medium (Md) and dark (Dk) pigment. (E) Testcrosses to assay whether B-I* segregates unchanged from + B-I*/Mop2-1 B' are diagramed in Figure S2. Plant pigmentation is shown from the +B-I*/+ B-P (parental class) and *+B'/+B-P* (recombinant class).

Paramutation at *b1* involves two alleles, paramutable *B-I* (*B-Intense*) and paramutagenic *B'* [54]. Phenotypes of *B-I* and *B'* are easily distinguished; the highly expressed paramutable *B-I* allele specifies high levels of purple anthocyanin pigment in most of the above ground organs (sheath, husk, and tassel), while the low expressed paramutagenic *B'* allele confers light speckled plant pigmentation (Figure 1A). The *B-I* allele is unstable and spontaneous paramutation to the low expressed *B'* state occurs at variable frequencies ranging from 0.1 to >50%, depending on the stock. In contrast to *B-I*, the silenced *B'* state is very stable and no change to higher expression has been observed in wild types backgrounds in many thousands of plants examined [12],[54],[55]. In addition to being very stable, the *B'* state is highly paramutagenic, when *B-I* and *B'* are combined in an heterozygote, *B'* always paramutates *B-I* resulting in all F_1_ progeny having light plant pigment [54]. In addition, the newly paramutated *B'* allele (*B-I* in the previous generation) is as efficient as the parental *B'* allele at causing paramutation of naïve *B-I* alleles [54].

The absolute penetrance of *b1* paramutation in wild type backgrounds, such that *B'* always changes the *B-I* allele to *B'*, was exploited to identify mutations required for *b1* paramutation. For this genetic screen *B'* pollen was treated with the chemical mutagen, ethyl methanesulfonate (EMS), and used to fertilize *B-I* ears (Figure 1A and Materials and Methods). The resulting M_1_ progeny were screened for rare dark purple plants, which would appear if *B'* failed to paramutate *B-I*, presumably due to the presence of a dominant mutation that prevented paramutation. One such exceptional dark plant was found among ∼7300 M_1_ plants (Figure 1A) and the putative mutation this plant carried was named *Mediator of paramutation2-1* (*Mop2-1*). The *B-I* allele that escaped paramutation was designated B-I* to indicate its exposure to *B'*, but that it remained *B-I* (Figure 1A). In subsequent generations the presence of a single copy of the *Mop2-1* mutation continued to protect *B-I* from paramutation by a newly introduced *B'* allele and this protection occurred independent of whether *Mop2-1* was transmitted through the male or female (Figure S1 and Figure S2; data not shown).

From the initial experiments it was apparent that when *Mop2-1* is heterozygous *B'* silencing is not relieved, as these plants are lightly pigmented (Figure 1B and Figure S1). It was also apparent that *Mop2-1* was loosely linked to *b1* (Figure S1). To examine whether the *Mop2-1* mutation when homozygous might relieve *B'* silencing, a family segregating *Mop2-1 B'/Mop2-1 B'* and *Mop2-1 B'/+ B'* plants was developed (Figure S2). If *B'* silencing was relieved in *Mop2-1* homozygous plants, then such plants would be expected to be darker relative to *Mop2-1 B'/+ B'* siblings. This expectation was met as the majority of homozygous *Mop2-1* plants (69%) showed increased pigmentation (Chart in Figure 1B), but none were as dark as *B-I* (Photo in Figure 1B). The remaining homozygous *Mop2-1* plants had medium dark (28%) or light (2%) pigment (Figure 1B) suggesting that the ability of *Mop2-1/Mop2-1* to relieve *B'* silencing was not fully penetrant.

To determine whether the increased expression of the *B'* allele observed in homozygous *Mop2-1* plants was a heritable change in the absence of *Mop2-1*, darkly pigmented *Mop2-1 B'* homozygous plants were crossed with the *B-I/B-P* tester (Figure S2). The *B-Peru* (*B-P*) allele does not participate in paramutation and confers essentially no plant color, which provides an excellent background for scoring *B'* and *B-I* pigmentation. Examination of *Mop2-1 B'/+ B-P* progeny revealed no dark plants (Figure 1C). The majority of plants had light *B'* pigment (87%), indicating that in these individuals *B'* was efficiently re-silenced in the presence of the wild type allele. The presence of some medium dark plants (13%) suggested that increased expression could be weakly heritable. Taken together, these results demonstrate that only when the *Mop2-1* mutation is homozygous is *B'* silencing relieved, unlike preventing paramutation where a single copy of the *Mop2-1* mutation was sufficient to prevent *B'* from silencing *B-I*.

The crosses described in Figure S2 were also used to assess the penetrance of the *Mop2-1/+* effects on *b1* paramutation in a larger population of plants. Analysis of the *Mop2-1 B'/+ B-I* * progeny demonstrated that 96% of these plants were darkly pigmented indicative of no paramutation and demonstrating that *Mop2-1* acts in a dominant and highly penetrant manner to prevent paramutation (Figure 1D). There were a few (5/126) *Mop2-1 B'/+ B-I* * plants that had a medium dark phenotype. These could result from either spontaneous paramutation of B-I* to *B'*, incomplete penetrance of *Mop2-1/+* in preventing paramutation, or both.

To confirm that B-I* segregates phenotypically unchanged from *B'* in *Mop2-1/+* plants, a backcross to the *B-I*/*B-P* stock was performed (Figure S2). Phenotypic and molecular markers were used to identify the + B-I*/+ B-P plants that are informative for B-I* heritability. If B-I* escaped paramutation in the previous generation, then, after accounting for recombination between the linked *b1* and *mop2* loci, assuming full *Mop2-1* penetrance, and absence of spontaneous paramutation (Figure S2), 73% should be parental (+ B-I*/+ B-P; dark plants) and 27% recombinant (*+B'/+B-P*; light plants). Results presented in Figure 1E demonstrate that light plants (22%), likely representing the *B'/B-P* progeny, were observed at a frequency close to the expected 27% (χ2 = 0.53, P = 0.46). The frequency of dark plants (53%) was lower than expected (χ2 = 7.7, P = 0.005), with medium dark plants observed at 24%. As there are no molecular markers to distinguish *B'* from the *B-I*, the medium dark plants could theoretically represent reduced expression of B-I* (because of spontaneous paramutation of B-I* to *B'*) or increased expression of *+B'/+B-P* recombinants. Independent of these hypotheses, the significant number of dark B-I* plants segregating demonstrates that the presence of one *Mop2-1* mutant allele in the previous generation can prevent paramutation. This finding is in sharp contrast to wild type backgrounds in which exceptional *B-I-*like plants have never been observed in thousands heterozygous *B'/B-I* plants grown over decades of experiments [12],[54],[55].

### *Mop2* encodes the second largest subunit of a plant-specific RNA polymerase

**Figure 2:**
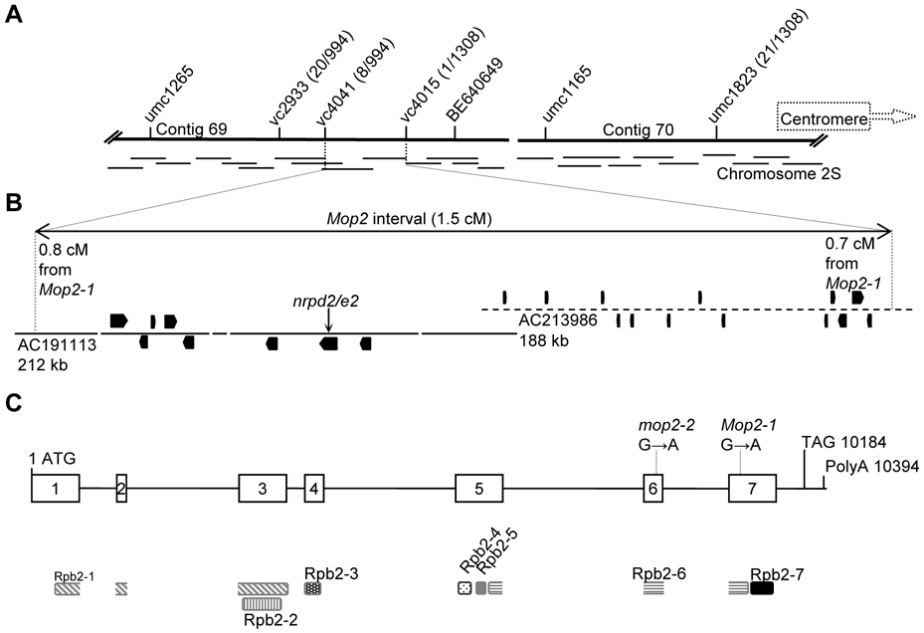
*Mop2-1* encodes a second-largest subunit of a plant-specific RNA polymerase. (A) Location of the *Mop2-1* interval on chromosome 2S is shown (FPC contigs 69–70). Polymorphic markers (indicated above the contigs) were used for mapping. The number of recombinants over the total number of plants screened are shown in parenthesis next to each marker. (B) An expanded map of the *Mop2-1* interval localized to two BACs. The dashed line is used for the AC213986 BAC because at the time of the publication it consisted of more then 30 unordered fragments. Predicted gene models within the two BACs were obtained from www.maizesequence.org. The predicted position and orientation of the *nrpd2/e2* gene (based on synteny with rice) is indicated. (C) Exons and introns of the *nrpd2/e2* gene based on alignment of genomic and cDNA sequences. The exons 1–7, translation start and stop, and polyadenylation sites are indicated. The positions of the G to A transitions in the *Mop2-1* and *mop2-2* alleles are shown. Location of the domains conserved with Pol-II RPB2 are shown below the exons. Domains were identified using the BLASTP program at http://pfam.sanger.ac.uk/search.

The *B'* and *B-I* stocks were differentially marked with two genes linked to *b1* (Figure 1A), which enabled following the original chromosomes carrying *B'* and *B-I* in subsequent generations. Presence of these markers enabled the initial observation that the *Mop2-1* mutation was loosely linked to *B'*, distal of *glossy2* (Figure S1). Screening of a large mapping population (Materials and Methods) further located the *Mop2-1* mutation to the 3.6 cM (13 BACs) interval spanning FPC Contigs 69 and 70 (Release 3b.50, February, 2009) (Figure 2A). Using additional molecular markers, the interval was further reduced to 1.5 cM, which consisted of two BAC clones (∼400 kb) on FPC Contig 69 (Figure 2B). Analysis of putative genes in this interval revealed a strong candidate, a *nrpd2/e2* gene, closely related to the Arabidopsis *NRPD2/NRPE2* gene encoding the second largest subunit of the Pol-IV and Pol-V plant specific RNA polymerases. Arabidopsis *NRPD2/NRPE2* is involved in regulating several epigenetic gene silencing phenomena [32],[33],[41],[42].

**Figure 3:**
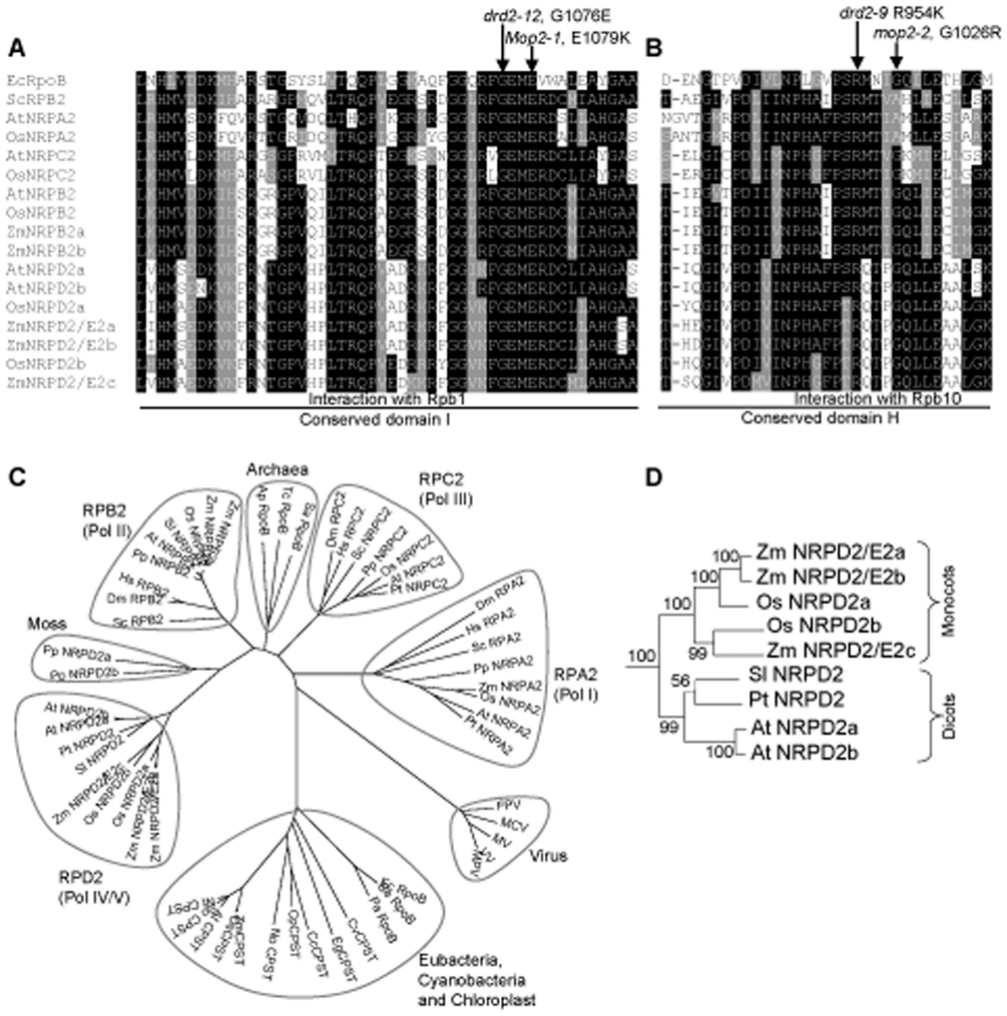
*Mop2-1* and *mop2-2* mutations are located in highly conserved motifs of the NRPD2/E2 proteins. (A) and (B) show excerpts from edited alignments of the second largest subunits of RNA polymerases with the positions of the *Mop2-1* and *mop2-2* mutations shown. Mutations in the single functional *NRPD2/E2* gene in Arabidopsis are also shown, *drd2-12* and *-9* [41]. Species designations are: Ec-*Escherichia coli*, Sc-*Saccharomyces cerevisiae*, At-*Arabidopsis thaliana*, Os-*Oryza sativa*, and Zm-*Zea mays*. The full alignment is in Figure S3. (C) Un-rooted radial bootstrap neighbor-joining phylogenetic tree of the second largest subunits of RNA polymerases. Species designations are shown in Table S1. (D) A portion of the phylogenetic tree shown in (C) with an expanded traditional view of the Pol-IV/Pol-V branch is shown. Bootstrap values are at the base of each branch.

Sequencing of the *nrpd2/e2* gene from this interval in the *Mop2-1* mutant revealed a transition mutation of guanine to adenine (G to A) relative to the progenitor allele, consistent with an EMS-induced mutation (Figure 2C). This change in DNA sequence led to a missense mutation of glutamic acid to lysine (E1079K), within the GEME motif, which is absolutely conserved [56] in Pol-I, Pol-II, Pol-III, and Pol-IV/Pol-V related polymerases from *E.coli* to higher eukaryotes (Figure 3A). The high conservation of the mutated residue strongly suggested that this change in *Mop2-1* would produce a mutant phenotype. The hypothesis that *mop2* is a *nrpd2/e2* gene was supported by the isolation of a second allele of *mop2* from an independent screen (Materials and Methods). The second allele, designated *mop2-2*, carries a G to A transition mutation relative to its progenitor, which is consistent with an EMS induced mutation. This mutation changes a glycine to arginine, (G1026R, Figure 2C) within another highly conserved domain (Figure 3B), distinct from that mutated in *Mop2-1*. Co-segregation analysis revealed that all plants homozygous for the *mop2-2* lesion had a dark plant phenotype consistent with the hypothesis that *mop2-2* disrupts *B'* silencing as a homozygote, similar to *Mop2-1*. Further experiments demonstrate that unlike *Mop2-1*, *mop2-2* is a recessive mutation as the establishment of paramutation is not prevented in heterozygotes (data not shown). Two mutations isolated in Arabidopsis *NRPD2/E2* are in the same domains as *Mop2-1* and *mop2-2* (Figure 3A), but as only homozygous phenotypes are reported [41], it is not clear if the Arabidopsis mutations also have dominant or semi-dominant phenotypes.

### Maize has three expressed genes that encode a second-largest subunit of Pol-IV/Pol-V–like polymerase in maize

**Figure 4:**
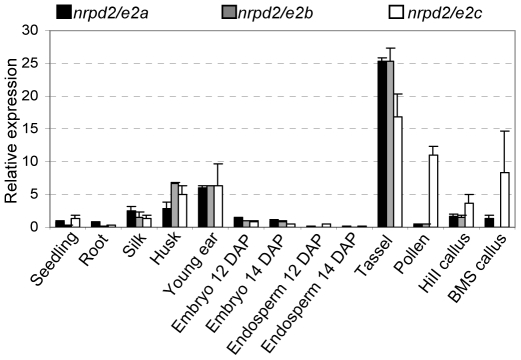
Maize *nrpd2/e2* genes are differentially expressed. Gene-specific primers and quantitative RT–PCR was used to analyze the RNA expression patterns of each of the three *nrpd2/e2* genes in multiple maize tissues from the B73 inbred line and HiII and BMS callus. Details on the developmental stages of the tissues are in Materials and Methods. Expression was normalized to *actin1* expression levels.

BLAST searches of the maize genome revealed that maize encodes three *nrpd2/e2* genes. In addition to the *mop2 nrpd2/e2* gene, designated *nrpd2/e2a*, located on chromosome 2S, there are two genes on chromosome 10: *nrpd2/e2b* on 10L, FPC Contig 418 (94% identity and 97% similarity to *nrpd2/e2a*); and *nrpd2/e2c* on 10S, FPC Contig 401 (67% identity and 79% similarity to *nrpd2/e2a*). Phylogenetic analysis demonstrated that the *nrpd2/e2a* and *nrpd2/e2b* genes are more similar to the presumed rice ortholog *OsNrpd2a*, while maize *nrpd2/e2c* is more similar to the other rice gene *OsNrpd2b* (Figure 3C and 3D). The more similar genes, *nrpd2/e2a* and *nrpd2/e2b* are located in recently duplicated blocks within the maize genome, while the more diverged *nrpd2/e2c* gene is located in a more anciently duplicated block [57]. High conservation within all of the critical polymerase domains (Figure S3) suggested that all three *nrpd2/e2* genes are likely to encode functional proteins. BLAST analysis indicated that *nrpd2/e2a* and *nrpd2/e2b* have multiple EST hits, whereas *nrpd2/e2c* had no significant EST hits in current databases. Lack of *nrpd2/e2c* ESTs could be either because of low expression, or because it is expressed in tissues under represented in the public EST datasets. To further explore the expression of all three genes, we carried out quantitative RT-PCR experiments using gene-specific primers. We detected expression of all *nrpd2/e2* genes in a wide range of tissues, but there was quantitative variation among the genes (Figure 4). For all three genes, the highest expression was in immature tassel and the lowest expression was in endosperm. The expression of *nrpd2/e2c* was more elevated in pollen and two callus samples (HiII and BMS) relative to *nrpd2/e2a* and *nrpd2/e2b* genes. Taken together these results demonstrate that all three maize *nrpd2/e2* genes are likely to be functional, in contrast with Arabidopsis where only one functional *nrpd2/e2* gene exists.

### *Mop2-1* reduces siRNAs levels, but does not alter transcription from the 853 bp tandem repeats that are required for *b1* paramutation

**Figure 5:**
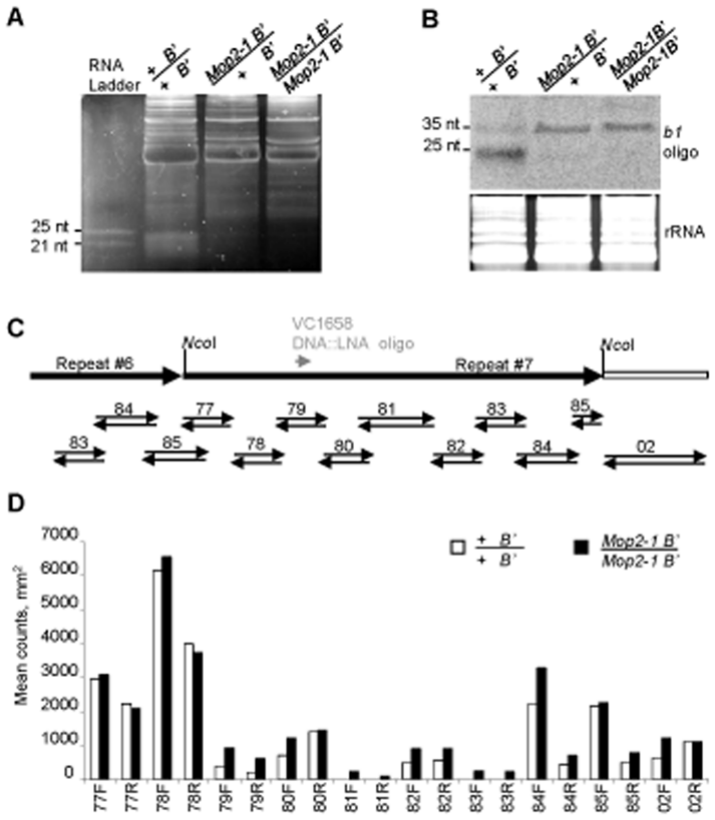
siRNA levels and transcription from the *B'* tandem repeats that mediate *b1* paramutation in *Mop2-1*. (A) Global siRNA levels in *Mop2-1* plants. The small RNA fraction was isolated from young ears (3–5 cm long) of *Mop2-1/Mop2-1*, *Mop2-1/+*, and +/+ plants and ∼100 ug samples were separated on a 15% denaturing polyacrylamide gel and stained with SyberGold. (B) Northern blot analysis of the siRNA fraction from young ears. The *b1* tandem repeat probe used for hybridization is indicated in (C). Staining with SyberGold is shown, which served as a loading control. (C) Drawing of a portion of the *B'* tandem repeats (black arrows) and the sequence immediately downstream (open rectangle). The position of the probes used are shown; the paired arrows indicate RNA probes used in the run-on analysis (D), while the gray arrowhead indicates the position of the DNA::LNA (locking nucleic acid) oligonucleotide used for the Northern blot analysis (B). (D) Results of nuclear run-on analysis of transcription within the seven *B'* tandem repeats in young ears. Letters indicate forward (F) or reverse (R) transcription, respectively, in relation to this drawing. Transcription levels were normalized to the transcription levels of the *Ubiquitin2* gene, measured as mean counts per mm^2^ (Materials and Methods). Two other independent experiments gave similar results (data not shown).

Mutations in the largest and second largest subunit of the Arabidopsis Pol-IV RNA polymerase cause dramatically reduced siRNA production [32],[58],[59]. Similarly, mutations in *rmr6*, which encodes the maize large subunit most similar to NRPD1 within the Pol-IV complex in Arabidopsis, show a dramatic reduction in siRNAs [31]. To determine if *Mop2-1* might reduce the function of a Pol-IV-like complex in maize, siRNA levels in *Mop2-1* were tested both globally and from the tandem repeats that mediate *b1* paramutation. The small RNA fraction was isolated from immature ears, which are a rich source of RNA, separated on gels, and stained with SyberGold. Staining revealed that global siRNA levels were dramatically reduced in both heterozygous (*Mop2-1/+*) and homozygous (*Mop2-1/Mop2-1*) samples (Figure 5A), consistent with the dominant phenotype of *Mop2-1*. We next asked whether levels of siRNAs from the 853 bp tandem repeats (Arteaga-Vazquez et al., in preparation) mediating *b1* paramutation [11] were altered in *Mop2-1*. In the wild type (+/+) background, two siRNA bands (prominent ∼25 nt and faint ∼35 nt) were detected in the *B'* allele (Figure 5B), which carries seven tandem 853 bp repeats and causes paramutation. In contrast, in the *B' Mop2-1* samples there was a dramatic reduction of the 24 nt band, while the ∼35 nt siRNAs appeared to increase (Figure 5B). Consistent with the dominant phenotypes that occur in *Mop2-1*, reduced levels of siRNAs were seen in both heterozygous and homozygous *Mop2-1* individuals, although there was more reduction in 24 nt siRNAs in homozygotes. Future experiments to examine whether the reduction of 24 nt siRNAs in *Mop2-1* is associated with reduced asymmetric (CHH) DNA methylation, as observed in Arabidopsis will be important to carry out.

Small RNAs larger than 24 nt have been reported in multiple species. In the ciliate protozoan, *Tetrahymena thermophila*, 27–30 nt RNAs direct developmentally directed DNA elimination [60], in mammals and zebrafish 26–31 nt PIWI-interacting RNAs are present in the germline [61],[62], and in Drosophila repeat associated RNAs direct retrotransposon and repetitive sequence silencing [63]. In Arabidopsis, the role of a specific 30 nt siRNA reported for *Flowering Locus C* is unknown [64] and 30–40 nt siRNAs are induced in response to pathogen infection or under specific growth conditions [65]. In our studies, the ∼35 nt *b1* tandem repeat RNAs were only observed when using the VC1658 LNA probe, one of four LNA probes for the *b1* tandem repeats that we have used (data not shown). One possibility is that transcripts from the LNA-VC1658 region are stable enough to detect alternative processing that increases when the predominant 24 nt pathway is disrupted. Further studies will be required to determine if the presence of the ∼35 nt siRNA class is significant.

The reduction in tandem repeat 24 nt siRNA levels in *Mop2-1* plants could theoretically be because *Mop2-1* is causing a reduction in transcription of the tandem repeats or a defect in processing. Previously, we showed that the 853 bp tandem repeats that mediate *b1* paramutation are transcribed [22]. To test whether transcription from the 853 bp repeats was altered in *Mop2*-*1*, we conducted nuclear run-on analyses from nuclei isolated from young ears, the same tissue used for siRNA analyses. The results presented in Figure 5D revealed no significant differences in transcription from the 853 bp repeats between wild type (*+ B'*) and homozygous *Mop2-1 B'* samples. This result indicated that *Mop2-1* did not disrupt transcription from the *b1* tandem repeats and suggested that the lack of 24 nt siRNAs is caused by a defect downstream of transcription. The dramatic reduction in 24 nt siRNAs is consistent with mutations in the large subunit of the Pol-IV complex in Arabidopsis and maize supporting the hypothesis that the *Mop2-1* mutation is disrupting the function of a Pol-IV-like complex in maize.

Our observations that there is no change in the transcription from the *b1* tandem repeats in *Mop2-1* mutants, further suggests that the major polymerase(s) responsible for this transcription is unlikely to be Pol-IV. Consistent with that hypothesis, other experiments suggest that the polymerase responsible for the bulk of the *b1* tandem repeat transcription is likely to be Pol-II as transcription is dramatically reduced with levels of alpha-amanitin that inhibit Pol-II (see Discussion).

### *Mop2-1* disrupts paramutation at multiple loci

**Figure 6:**
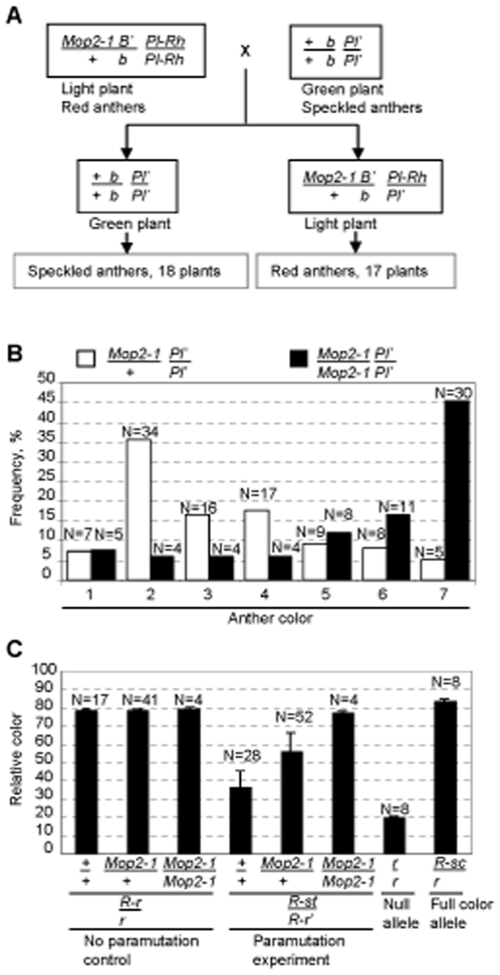
The *Mop2-1* mutation alters paramutation at *pl1* and *r1*. (A) *Mop2-1* prevents *pl1* paramutation. Paramutation occurs when paramutable *Pl-Rh*, specifying dark red anther pigment and paramutagenic *Pl'*, conferring light speckled anther pigment are brought together in a wild type background (18 individuals). In contrast, when paramutable *Pl-Rh* is exposed to paramutagenic *Pl'* in the *Mop2-1/+* background *Pl'* fails to paramutate *Pl-Rh* and dark anthered plants are observed (17 individuals). (B) *Mop2-1* effect on *Pl'* silencing. Silencing associated with *Pl'* paramutation was assayed in progeny from crosses between *Pl'*; *Mop2-1 B'/+ B'* and *Pl'*; *Mop2-1 B'* plants. Histograms show distribution of anther color scores [3] in which lightest pigment is 1 and solid red pigment is 7. The number of plants in each class is shown above the corresponding bar. The majority of *Mop2-1* homozygotes have dark anthers (anther scores 5–7) suggesting *Pl'* silencing is relieved, while in most *Mop2-1/+* heterozygotes *Pl'* silencing is maintained (anther scores 1–4). The presence of a few plants with light anthers among *Mop2-1* homozygotes suggests that *Mop2-1* is not completely penetrant in relieving *Pl'* silencing. As reversion of *Pl'* to *Pl-Rh* is never observed in wild type plants [3], the observation of some *Mop2-1/+* plants with dark anthers suggest *Mop2-1* is partially dominant for relieving *Pl'* silencing. (C) Effect of *Mop2-1* on preventing *r1* paramutation. Details of the crosses are in Figure S5. The paramutable *R-r* allele (solid or dark mottled seed) was exposed to the paramutagenic *R-st* allele (stippled seed) in the presence of the *Mop2-1* mutation (homozygous or heterozygous) or wild type. In wild type, *R-st* paramutates *R-r* (designated as *R-r'*) and lighter mottled seed color is observed. In *Mop2-1* heterozygotes and homozygotes, *R-st* fails to fully paramutate *R-r*, resulting in medium mottled or darkly mottled to solid seed color, respectively. Colorless *r* alleles and solid colored *R-sc* alleles were pigmentation standards.

Paramutation has been well characterized at three other maize loci: *pl1*, *p1* and *r1*, all encoding transcription factors that activate pigment synthesis [4],[7],[9],[66]. We were interested in determining whether *Mop2-1* disrupted paramutation at these loci and, if so, whether disruption was similar to *b1* paramutation, *i.e.*, when heterozygous *Mop2-1* prevented paramutation and when homozygous it increased the expression of silenced alleles. Table 1 summarizes the alleles for each gene used in each experiment and the results. For each of these paramutation systems, genetic backgrounds were available that enabled the monitoring of paramutation through changes in pigment levels (Materials and Methods). To investigate paramutation at each locus, *Mop2-1* was introduced into the appropriate genetic background (Figure 6, Table 2, Figure S4, and Figure S5) and pigment was monitored in the appropriate tissues.

**Table 1:** Paramutation phenotypes at multiple loci in *Mop2-1* plants.

Locus^a^	Paramutagenic allele	Paramutable allele	Prevents paramutation^b^	Releases silencing^b^	Data
*b1*	*B'* (light plant)	*B-I* (dark purple plant)	dominant	recessive	Figure1
*pl1*	*Pl'* (speckled anthers)	*Pl-Rh* (purple anthers)	dominant	semi-dominant	Figure 6A, 6B
*p1*	*P1-rr'* (patterned pericarp)	*P1-rr* (red pericarp)	recessive	no^c^	Table 2
*r1*	*R-st* (stippled)	*R-r* (purple seeds)	semi-dominant	no dominant phenotype^d^	Figure 6C

**a** At *b1*, *pl1* and *p1* the paramutagenic and paramutable alleles are epialleles, *i.e.* they have exactly the same DNA sequence, but distinct expression and chromatin states. In contrast, the paramutagenic *R-st* and paramutable *R-r* alleles are structurally distinct [for a review, see [7]]. When *R-r* is paramutated by *R-st*, it is referred to as *R-r'*; *R-r* and *R-r'* are epialleles.

**b** The prevention of paramutation is assayed when a paramutagenic and paramutable allele are combined in plants homozygous or heterozygous for *Mop2-1*. The test for release of silencing assays pigment levels when a previously paramutated (silenced) allele is exposed to heterozygous or homozygous *Mop2-1*.

**c** Three sequential generations carrying *P1-rr'* in *Mop2-1* homozygotes (40 total ears) showed no increased expression of *P1-rr'*.

**d** *Mop2-1/+* did not release *R-r'* silencing previously established by *R-st*. Tests with homozygous *Mop2-1* were inconclusive because paramutated *R-r'* reverted at high frequency to a highly expressed state similar to *R-r* regardless of the *Mop2-1* genotype.

**Table 2:** *Mop2-1/Mop2-1* prevents establishment of *p1* paramutation^a^.

*Mop2-1* genotype^b^	Red pericarp	Orange pericarp	Patterned or colorless pericarp	Total number of ears
*Mop2-1/+*		1	24	25
*Mop2-1/Mop2-1* c	5	2		7

**a** Paramutation at *p1* is observed in the pericarp (seed coat), where the paramutable *P1-rr* allele specifies dark red pericarp pigment. When paramutated, by exposure to paramutagenic *P1-rr'* and the P1.2b::GUS transgene (transgenic event P2P147-37, [13]), pigmentation of paramutable *P1-rr* is reduced to colorless or light patterned pericarp. Details of experiment are in Figure S4.

**b** Molecular genotyping was used to determine the *Mop2-1* genotype.

**c** The negative pleiotropic developmental phenotypes in *Mop2-1* homozygotes result in frequent abortion of ears resulting in only a small number of *Mop2-1/Mop2-1* homozygous ears that can be pollinated and produce sufficient seed to score the *p1* pigment in mature ears.

The results at the *pl1* locus resembled *b1* paramutation; *Mop2-1* was dominant for preventing *pl1* paramutation (Figure 6A) and it relieved the silencing of the paramutated *Pl'* allele (Figure 6B). However, the levels of increased expression of the paramutant *Pl'* allele were variable suggesting *Mop2-1* was partially dominant (Figure 6B). In contrast to what was observed at the *b1* and *pl1* loci, *Mop2-1* prevented *p1* paramutation only when homozygous and there was no change on the expression of the silenced allele even after multiple generations of exposure to *Mop2-1* (Table 1 and Table 2). At the third locus tested, *r1*, *Mop2-1* was semi-dominant for preventing paramutation (Figure 6C). These results demonstrated that the *Mop2-1* mutation differentially alters paramutation at these loci. Potential reasons for this are explored in the Discussion.

### *Mop2-1* is required for transcriptional silencing mediated by two transgenes expressing inverted repeat promoters

**Figure 7:**
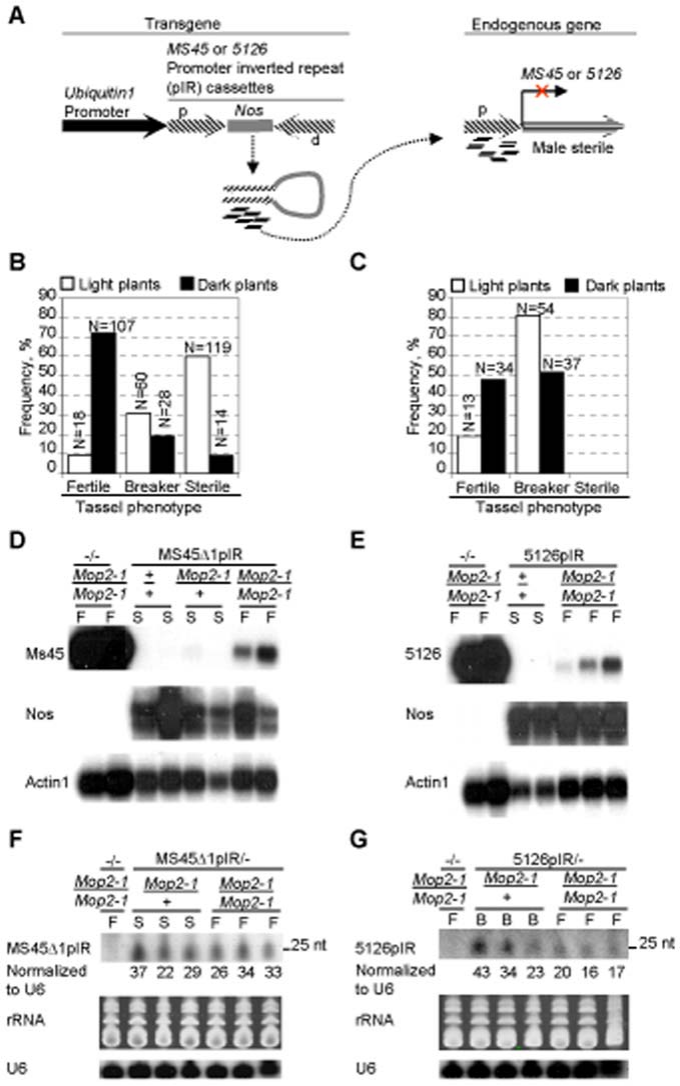
*Mop2-1* disrupts pIR transgene-mediated silencing at the *Ms45* and *5126* loci. (A) Outline of the experiment with the pIR transgenes. The strong constitutive *Ubiquitin1* promoter was used to transcribe inverted repeats containing *Ms45* and *5126* promoter fragments, which results in siRNA that target promoters of the endogenous *Ms45* and *5126* genes for silencing. Silencing of the *Ms45* and *5126* genes leads to male sterility in wild type plants. (B) Disruption of pIR silencing of the *Ms45* locus in *Mop2-1* plants. The chart shows frequencies of fertile, breaker (extruded anthers), and sterile plants among dark (homozygous) and light (heterozygous) *Mop2-1* plants. The number of plants in each group is shown above each bar. (C) Disruption of pIR silencing of the 5126 locus in *Mop2-1* plants. Frequencies of fertile and breaker phenotypes are shown, with the number of plants in each group above each bar. (D) and (E) show Northern blot analyses on samples from non transgenic siblings (first two lanes) and from Ms45Δ1pIR and 5126pIR transgenic lines, respectively (remaining lanes). PolyA enriched RNA samples from anthers containing quartet/early uninucleate microspores were used for Northern blot analysis. Tassel phenotypes are indicated with F for fertile and S for sterile. Probes used for hybridization are indicated on the left of each panel. *Actin1* was used as a loading control. (F) and (G) show results from Northern blot analyses for Ms45Δ1pIR and 5126pIR transgenic siRNAs in heterozygous and homozygous *Mop2-1* plants and non transgenic controls (−/−). Hybridization with the U6 probe and SyberGold staining of rRNA served as loading controls. The numbers below each lane indicate the quantification of the transgene siRNA levels normalized to U6. Tassel phenotypes are indicated: S for sterile, B for breaker and F for fertile.

Roles for the Arabidopsis Pol-IV and Pol-V polymerase complexes in transcriptional silencing mediated by siRNAs generated from the expression of promoters in inverted repeats (pIR) are well documented [67],[68]. To test whether *nrpd2/e2a* might be involved in a similar transgene-mediated silencing system in maize, we tested whether the *Mop2-1* mutation could prevent the silencing of two pIR-targeted loci (Figure 7A), each required for male fertility [67]. One of these genes, *Ms45* is expressed in the anther tapetum during early vacuolate stage of pollen development and is required for microspore development [67]. In wild type backgrounds, targeting the *Ms45* gene by the Ms45Δ1pIR inverted repeat transgene results in complete sterility; 100% of tassels do not extrude anthers and they fail to produce any pollen [67]. To test whether *Mop2-1* disrupts silencing, transgenic Ms45Δ1pIR/- plants were used as females and pollinated with the homozygous *Mop2-1 B'* stock (Figure S6). In the first generation, all plants were heterozygous (*Mop2-1/+*); if *Mop2-1* disrupted this process as a dominant or semi-dominant mutation, then full or partial restoration of male fertility would be expected. Examination of the *Mop2-1/+* plants revealed that although all plants remained male sterile, small shriveled anthers developed in many of the plants (data not shown). Because Ms45Δ1pIR leads to complete absence of anthers in wild type plants, improved anther development in *Mop2-1/+* plants was a significant finding, and suggested *Mop2-1* was semi-dominant. To test whether homozygous *Mop2-1* plants would show a more dramatic relief of *Ms45* silencing, transgenic plants were pollinated with the *Mop2-1 B'* stock (Figure S6). The resulting herbicide resistant plants segregating heterozygous and homozygous *Mop2-1* individuals were examined. Because the *B'* allele was introduced together with the *Mop2-1* mutation, dark plant pigmentation was used to initially identify *Mop2-1* homozygous plants in the segregating families. If *Mop2-1/Mop2-1* prevents pIR transgene-induced silencing of *Ms45*, dark plants would be expected to exhibit partial or complete restoration of male fertility. This expectation was met as 72% of the dark plants were fertile, 19% had small anthers that did not shed pollen (referred to as breakers), and only 9% were sterile (Figure 7B). These results indicated that Ms45Δ1pIR-induced silencing of the *Ms45* gene was disrupted in the majority of the darkly pigmented plants, likely representing *Mop2-1*/*Mop2-1* homozygotes. The majority of light *B'* plants (60%), mostly representing *Mop2-1/+*, were completely sterile, but a few were fertile (9%) or had the breaker phenotype of extruded sterile anthers (31%) (Figure 7B). The detection of fertile plants among light *B'* plants could result from cumulative effects of carrying *Mop2-1/+* for two generations, or incomplete correspondence between the *Mop2-1* genotype and release of *B'* silencing. Both explanations are likely to be occurring because molecular genotyping revealed that 28/30 dark plants were homozygous and 28/33 of light plants were heterozygous for *Mop2-1*. Altogether, these results demonstrated that the *Mop2-1* mutation can prevent the Ms45Δ1pIR transgene from silencing the endogenous *Ms45* gene in a semi-dominant manner, with the strongest phenotypes observed in homozygotes.

A second pIR-targeted locus was tested for whether *Mop2-1* could disrupt silencing. The *5126* gene has also been demonstrated to be expressed during microsporogenesis [67]. The 5126pIR-induced silencing of the *5126* gene results in complete male sterility, but a portion of tassels do show the breaker phenotype, *i.e.*, 30% of the flowers on a tassel extrude small shrunken anthers that do not contain pollen [67]. Similar to the results with the Ms45Δ1pIR/− transgenes, some improvement of anther development of the 5126pIR/− transgenic plants was noted in the first generation of plants heterozygous for *Mop2-1/+*; unlike wild type backgrounds some plants extruded anthers that produced small amounts of pollen (unpublished data). After a backcross with the homozygous *Mop2-1* stock (Figure S6), families segregating *Mop2-1/Mop2-1* and *Mop2-1/+* progeny were examined. Almost half of the darkly pigmented *Mop2-1* homozygous plants had fertile tassels (48%), while the other half had the breaker phenotype (Figure 7C). There were fertile plants (19%) among light *B' Mop2-1/+* heterozygous plants, although most showed the breaker phenotype (81%). These results indicate that the *Mop2-1* mutation can disrupt pIR silencing at the *5126* locus in a semi-dominant manner, with increased activity as a homozygote.

We also tested whether *Mop2-1* disrupted pIR-mediated silencing of a third transgene, pg47pIR, which targets the *pg47* locus, a gene that is highly expressed during pollen development [68],[69]. In contrast to the results with Ms45Δ1pIR and 5126pIR, the *Mop2-1* mutation did not reverse pg47pIR-mediated silencing of the *pg47* gene family (Cigan M, unpublished data). The *pg47* gene is expressed in pollen, a tissue where *nrpd2/e2a* is expressed at a very low level and *nrpd2/e2c* is expressed highly (Figure 4). Thus, we favor the hypothesis that *nrpd2/e2a* is not used extensively in the pollen, and therefore a mutation in it has no phenotype in this tissue. However, an alternative explanation that silencing induced by different pIR transgenes may use distinct mechanisms can not be eliminated. Taken together, these results suggest that a mutation in *nrpd2/e2a* disrupts pIR-mediated silencing, but not at all loci.

### *Mop2-1* heterozygotes and homozygotes have similar levels of Ms45Δ1pIR and 5126pIR transgene transcripts and siRNAs

In Arabidopsis, Pol-IV is required for siRNA production, whereas Pol-V primarily acts downstream of siRNA production generating non-coding transcripts and helping to direct chromatin modifying enzymes to target loci [41],[42],[45],[70]. To gain insight into possible mechanisms by which the *Mop2-1* mutation disrupts pIR-mediated silencing in maize, we assayed transcript levels of the Ms45Δ1pIR and 5126pIR transgenes and the targeted endogenous genes in *Mop2-1* plants. Results demonstrated that endogenous *Ms45* and *5126* transcripts were absent in sterile +/+ or *Mop2-1/+* plants consistent with silencing and that they were present in fertile *Mop2-1/Mop2-1* plants (upper panels in Figure 7D and 7E), consistent with *Mop2-1* preventing silencing. However, the levels of the endogenous genes' transcripts in *Mop2-1/Mop2-1* plants were not as high as in non-transgenic plants (upper panels in Figure 7D and 7E), indicating that partial silencing was still occurring even in homozygous *Mop2-1* plants, although clearly enough transcription of the endogenous genes was occurring to restore fertility. Hybridization with the Nos spacer fragment probe [67], which is unique to the Ms45pΔ1IR and 5126pIR transgenes, revealed that transgene transcript levels, while variable, were similar between sterile (*+/+* or *Mop2-1/+*) and fertile (*Mop2-1/Mop2-1*) plants (Figure 7D and 7E). Thus, *Mop2-1* is not acting to reduce the pool of transgenic transcripts that serve as precursors for siRNA production and silencing.

Because the *Mop2-1* mutation reduced levels of total endogenous siRNAs and the *b1* 853 bp repeat specific siRNAs, we tested whether *Mop2-1* might also dramatically reduce the siRNAs produced from the Ms45pΔ1IR and 5126pIR transgenes. Because we did not have wild type transgenic lines that were isogenic with the *Mop2-1* heterozygotes and homozygotes, we compared the transgene siRNA levels in sterile and breaker heterozygous *Mop2-1/+* plants to those in fertile homozygous *Mop2-1/Mop2-1* plants. If the ability of *Mop2-1* to relieve silencing was due solely to reduced siRNA biogenesis, we would expect few transgenic siRNAs to be seen in fertile homozygotes. Results in Figure 7F demonstrated that 24 nt siRNAs are produced at similar levels from the Ms45Δ1pIR transgene in both fertile *Mop2-1* homozygous plants and sterile *Mop2-1/+* heterozygous plants. In the 5126pIR transgenic plants transgene siRNAs were also detected in both breaker *Mop2-1* heterozygotes and fertile homozygotes, although two out of three *Mop2-1* heterozygotes had approximately two fold higher siRNA levels relative to homozygotes (Figure 7G). These results demonstrated that the ability of the *Mop2-1* mutation to restore male fertility in the Ms45pΔ1IR and 5126pIR transgenic plants was not simply because it eliminated transgene siRNAs. One possibility is that *Mop2-1* can act downstream of siRNA production to relieve sterility. As Pol-V in Arabidopsis acts downstream of siRNA biogenesis, this hypothesis is consistent with *Mop2-1* also disrupting a Pol-V-like complex in maize. Alternative explanations are presented in the Discussion.

### *Mop2-1* plants exhibit deleterious pleiotropic developmental phenotypes

Mutations in Arabidopsis Pol-IV/Pol-V complexes have not been reported to display major developmental phenotypes, except for a delay in flowering [32],[41]. In contrast, *Mop2-1* displays a number of developmental phenotypes, but these are less dramatic and more variable than the developmental phenotypes observed with mutations in the *rmr6* gene [21], which encodes the large subunit of a Pol-IV like complex in maize [31]. When propagating the *Mop2-1* mutation through numerous generations, a number of phenotypes were routinely observed including reduced transmission, altered flowering time and abnormal developmental phenotypes. This was similar to phenotypes of *mop1* mutations [20], but different from *rmr6* mutations, as the aberrant phenotypes were not observed in every plant carrying the *Mop2-1*or *mop1* mutations, but were observed in all plants homozygous for *rmr6* mutations [21].

The types of abnormal morphological and development phenotypes that we observed in stocks segregating *Mop2-1* were reduced plant height, skinny plant stature, tassel seed, failure to develop an ear, and poor seed set in ears that did develop. These phenotypes are variable and they occur in both heterozygous or homozygous *Mop2-1* plants, but they are more frequent and more severe in homozygous *Mop2-1/Mop2-1* plants. For example, in one experiment *Mop2-1/Mop2-1* plants were on average 12 cm shorter and flowered 4.2 days later then heterozygous siblings, and 22% of *Mop2-1/Mop2-1* versus 10% of *Mop2-1/+* siblings failed to differentiate ears. These negative pleiotropic phenotypes on plant health and reproduction influenced how the stocks could be maintained such that self pollinations were rarely successful and large numbers of crosses were required to obtain sufficient numbers of homozygous *Mop2-1* plants with mature ears and reasonable seed set for our experiments.

To determine the transmission of *Mop2-1*, 231 plants were genotyped for the *Mop2-1* mutation from a family that would be expected to segregate equal numbers of *Mop2-1* homozygous and heterozygous plants if there was no reduction in *Mop2-1* transmission. This experiment revealed that the number of *Mop2-1* homozygotes was reduced (39%) (χ = 211.2, P = 0.0001). To test whether this was due to reduced transmission or reduced germination frequency, we genotyped 94 seeds directly and observed a similar reduced number of *Mop2-1* homozygotes, only 39% instead of the 50% expected for normal transmission. This strongly suggested reduced transmission was contributing to the reduced number of *Mop2-1* homozygotes.

The observation of developmental phenotypes with *Mop2-1* differs from the recessive loss-of-function truncation mutations, which lack developmental phenotypes (Stonaker et al., this issue). This is not simply because *Mop2-1* is dominant and the mutations isolated in the Hollick lab are recessive as we also see developmental phenotypes with our recessive *mop2-2* allele. Although we haven't grown *mop2-2* plants for as many generations as *Mop2-1* plants, homozygous *mop2-2* plants are very skinny and rarely set seed. One possibility is that our two missense mutations are having broader effects relative to the null mutations isolated in the Hollick lab. Another possibility is that the genetic background of the null mutants is suppressing developmental phenotypes, or the genetic background of our mutants is enhancing developmental phenotypes. It has long been known that different genetic backgrounds can enhance or suppress developmental phenotypes in maize [71]. A third possibility is that the more extreme environmental growth conditions in Arizona relative to Northern California are enhancing the developmental phenotypes.

## Discussion

### Maize contains multiple *nrpd2/e2* genes with overlapping expression patterns

Our results demonstrate that *mop2*, a key gene involved in paramutation at multiple loci, encodes a protein closely related to the second largest subunit of the plant-specific RNA polymerase complexes, Pol-IV/Pol-V, first described in Arabidopsis. Unlike Arabidopsis, which encodes only one functional protein (NRPD2/E2), which is in both the Pol-IV and Pol-V complexes [32],[41],[42],[58], maize encodes three closely related genes. These three genes are likely to encode functional proteins as they are full length, have all of the polymerase domains conserved and are expressed in multiple tissues. This observation suggests that multiple Pol-IV/Pol-V-like complexes and potentially even novel complexes may exist in maize. Potentially these could have diversified to function at different loci, different developmental stages, or under different environmental stresses. Multiple Pol-IV/Pol-V related complexes could confer greater complexity and epigenetic regulatory capacity to maize as compared to Arabidopsis.

Our expression analyzes revealed that all three maize genes are widely expressed in multiple organs and tissues, similar to that of *NRPD2/E2* in Arabidopsis [42],[70]. All three maize genes are most highly expressed in maize reproductive organs such as tassels and immature ears. The most diverged gene, *nrpd2/e2c*, is the major gene expressed in pollen and it is also more highly expressed in callus relative to the other two genes. Given this difference in expression and that the phylogenetic analyses suggested *nrpd2/e2c* represents a more ancient duplication, it is the most likely candidate for a distinct function relative to *nrpd2/e2a,b*. It is striking that the two most similar genes, *nrpd2/e2a* and *nrpd2/e2b* share similar expression patterns across all tissues tested, yet our results demonstrate that a recessive mutation in *nrpd2/e2a*, *mop2-2*, has paramutation defects. This demonstrates that at least with respect to paramutation, the n*rpd2/e2a* gene is unlikely to be functionally redundant with *nrpd2/e2b*. This hypothesis is supported by studies from the Hollick lab in which recessive mutations in the same gene were isolated in forward genetic screens for *pl1* paramutation (Stonaker et al, this issue). These three genes may not be functionally redundant either because they function in distinct complexes or because there are differences in their expression patterns not detectable with quantitative RT-PCR in complex multi-cellular tissues. Expression differences among different cell types within organs, distinct cell layers within the same tissue, differences in subcellular location, or even locus specific distribution are all possible explanations for the lack of functional redundancy. Given the high degree of similarity among all three genes, the generation of transgenic plants with differentially tagged genes as well as mutations in the other two subunits will enable these hypotheses to be distinguished.

### *Mop2-1* may be disrupting both Pol-IV–like and Pol-V–like complexes

The loss of siRNAs from repetitive elements throughout the genome and from the tandem repeats that are required for *b1* paramutation is consistent with *Mop2-1* disrupting a Pol-IV like complex. When siRNAs are produced independently of Pol-IV as they are in many pIR transgene experiments [41], *i.e.* potentially in our case when the strong Pol-II promoter from the maize *ubiquitin1* gene is used to produce inverted repeat transcripts, one can also examine the potential for *Mop2-1* to reduce Pol-V like function, downstream of siRNA biogenesis. In unpublished data we have shown that the pIR transgenes we used do not require the endogenous siRNA biogenesis pathway for silencing, as the null *mop1-1* mutation (in the RDR orthologous to RDR2), which dramatically reduces 24 nt siRNAs does not prevent pIR silencing (M. Cigan and V. Chandler, unpublished data). In *Mop2-1* homozygotes pIR-mediated silencing is relieved, yet there are similar levels of siRNAs from the pIR transgenes in fertile homozygotes relative to sterile heterozygotes. This result is consistent with *Mop2-1* also acting downstream of siRNA biogenesis, potentially by disrupting a Pol-V like complex. However, it is possible that *Mop2-1* may only be acting through a Pol-IV-like complex. For example, *Mop2-1* may partially impair secondary siRNA production from the pIR transgenes, which might partially reduce transcriptional silencing resulting in fertility. This model can account for *Mop2-1* effects entirely through Pol-IV deficiencies.

If *Mop2-1* does alter both Pol-IV and Pol-V functions, it could be because like Arabidopsis, the wild type *nrpd2/e2a* encoded subunit functions in both Pol-IV-like and Pol-V-like RNA polymerase complexes. Alternatively, the *Mop2-1* mutation may confer a gain of function phenotype that enables the mutant subunit to interact with and disrupt complexes the wild type subunit normally does not form. The observation that loss of function mutations in *nrpd2/e2a* also cause a dramatic reduction in global siRNAs (Stonaker et al, this issue), suggests that at a minimum NRPD2/E2a functions in a Pol-IV like complex.

### Model for the dominance of *Mop2-1* and potential explanations for dependence of certain phenotypes on *Mop2-1* dosage

The *Mop2-1* mutation's effects on paramutation at multiple loci, pIR-mediated silencing, and plant growth and development depend on the dosage of the *Mop2-1* allele with some phenotypes more variable than others. Certain phenotypes were observed with high penetrance when *Mop2-1* was heterozygous (dominant for prevention of *b1* and *pl1* paramutation, reduction in global and *b1* tandem repeat siRNAs); some phenotypes required *Mop2-1* to be homozygous (recessive for release of *B'* silencing and prevention of *p1* paramutation); and still other phenotypes were seen in *Mop2-1* heterozygotes, but were much stronger in *Mop2-1* homozygotes (semi-dominant for prevention of *r1* paramutation, disrupting pIR-mediated silencing of *Ms45* and *5126*, release of *Pl'* silencing, further reduction of *b1* tandem repeat siRNAs, and many developmental phenotypes). There were also phenotypes that *Mop2-1* did not alter (no release of *P1-rr'* silencing and no disruption of pIR-mediated silencing of *pg47*). Below we discuss a model for *Mop2-1* dominance, based on data from similar mutations in the second largest subunit of *E.coli* RNA Polymerase, and suggest explanations for the dependence of specific phenotypes on *Mop2-1* dosage.

The GEME motif mutated in *Mop2-1* is nearly invariant among all second largest subunits in all polymerases from organisms ranging from bacteria, fungi, animals, and plants. The *Mop2-1* mutation changes the second glutamic acid residue (E1076) of the GEME motif to a lysine. In RpoB (the second largest subunit of *E.coli* RNA polymerase) the GEME motif is located within the “anchor” region required for interaction with the clamp fold within the largest polymerase subunit [72]. The clamp swings open to produce a larger opening of the cleft that permits entry of promoter DNA and subsequent initiation of transcription. Extensive mutagenesis of the second largest subunit, *rpoB* in *E.coli* revealed that mutations within all four GEME motif amino acids result in dominant phenotypes when the mutant subunit is produced from a plasmid at similar levels to the chromosomal encoded non-mutant copy [56]. Substitutions in this region produced a RNA polymerase that competed with the wild type RNA polymerase complex potentially because the mutant polymerase was blocked after transcription initiation [56],[73]. The strength of the dominant phenotypes varied depending on the specific substitution.

The extreme conservation of the GEME motif [56] and the results in *E.coli*, lead us to hypothesize that a similar molecular mechanism contributes to the dominant phenotype of the *Mop2-1* mutation. Our model is that NRPD2/E2a proteins with the *Mop2-1* mutation associate normally with the same largest subunit(s) (NRPD1 and potentially NRPE1, or both) that the wild type subunit associates with, forming mutant polymerase complexes that are functionally defective, but that efficiently compete with wild type polymerase complexes. This model predicts that the relative dosage of mutant and wild type subunits would influence the number of functional complexes available. Assuming that the *Mop2-1* encoded protein is expressed equivalently to the wild type, a *Mop2-1* heterozygote should have equal amounts of wild type and mutant proteins, such that phenotypes that are particularly sensitive to Pol-IV or Pol-V dosage would be altered in the heterozygote. Moreover, processes that require both Pol-IV and Pol-V complexes might show more dramatic phenotypes than processes that require either Pol-IV or Pol-V alone, if there are additive consequences of partial loss of each complex. This model further predicts that a *Mop2-1* homozygote, which would have no wild type NRPD2/E2, would produce a stronger phenotype relative to the heterozygote, potentially equivalent to a loss of function mutation.

The presence in maize of two other second largest subunits, including NRPD2/E2b that is 94% identical to NRPD2/E2a, provides a further complication and could also contribute to different phenotypes between *Mop2-1* heterozygotes and homozygotes if the *Mop2-1* encoded NRPD2/E2a protein competes with the other subunits for complex binding. This could lead to gain of function phenotypes, in which the *Mop2-1* encoded NRPD2/E2a subunit is partially poisoning complexes that normally carry the NRPD2/E2b, c subunits. Competition would be postulated to be most effective in homozygous *Mop2-1* plants where the dosage of the mutant subunit is highest. As stated previously, detailed biochemical analyses with each of the subunits differentially tagged will be necessary to begin to distinguish among possible models.

### New insights on roles of Pol-IV/Pol-V–like polymerases in *b1* paramutation

As *b1* paramutation is the most extensively characterized system at the molecular level, we will limit our discussion to the *b1* system. The *Mop2-1* mutation prevents *b1* paramutation when heterozygous and releases silencing of *B'* when homozygous indicating Pol-IV/Pol-V-like RNA polymerases are required for *b1* paramutation. While the requirement for a Pol-IV-like polymerase for *b1* paramutation was previously demonstrated in studies with a mutation in the large subunit most similar to Pol-IV in Arabidopsis [21],[31], our results provide further clarification on potential roles for Pol-IV/Pol-V-like complexes at distinct steps in paramutation.

*Mop2-1* reduces siRNA production, characteristic of a Pol-IV-like mutation, but does not reduce transcription from the *b1* tandem repeats, suggesting that NRPD2/E2a containing RNA polymerase complexes do not significantly contribute to *b1* repeat transcription. Transcription from the *b1* repeats is sensitive to actinomycinD, indicating that these transcripts are produced from DNA templates (Arteaga-Vazquez et al., in preparation). In Arabidopsis, Pol-V has been shown to use DNA as a template to produce non-coding transcripts [45]. Transcripts produced from Pol-V in Arabidopsis are rare [45], and if a similar situation exists in maize, differences between presumed Pol-V mediated transcription in wild type and homozygous *Mop2-1* plants might be difficult to detect with nuclear run-ons, especially if most of the *b1* repeat transcription is mediated by Pol-II. Transcription from the *b1* repeats is highly sensitive to alpha-amanitin consistent with Pol-II being the best candidate for the polymerase performing the major transcription of the non-coding *b1* repeats (Arteaga-Vazquez et al., in preparation). Further molecular and biochemical characterization of *b1* repeat transcripts will determine whether these transcripts are polyadenylated, and chromatin immunoprecipitation assays with tagged Pol IV and Pol V complexes will determine if either physically interact with the *b1* tandem repeats mediating paramutation.

Our observation that silenced alleles are not up-regulated in *Mop2-1* heterozygous plants, in spite of the dramatic reduction in *b1* tandem repeat siRNAs, suggests that the major role for the tandem repeat siRNAs is not to maintain silencing. Instead, we hypothesize that the tandem repeat siRNAs may mediate the allele communication that establishes the heritable silent state; a process that is fully disrupted in *Mop2-1* heterozygotes. Use of the *Mop2-1* plants in future experiments may enable us to separate mechanisms operating at the initial establishment of paramutation from those operating subsequently to maintain silencing. Previously this has not been possible as establishment can only be observed if it is heritably maintained, so mutations defective in maintaining silencing will also appear defective in establishment. However, mutations that do not relieve silencing, but do prevent the establishment of paramutation such as *Mop2-1* (when heterozygous) provide a system to investigate mechanisms for establishment.

## Materials and Methods

### Generation of *Mop2-1* and *mop2-2* mutations

The *Mop2-1* mutation was generated by treating *Gl2 B' Wt*; *Pl-Rh*; *r-g* (inbred W23 background) pollen with ethyl methanesulfonate [74]. Treated pollen was used to pollinate *gl2 B-I wt*; *Pl-Rh*; *r-g* [*W23/K55*] ears, producing M_1_ seed (Figure 1A). In wild-type stocks, *B'* will always paramutate *B-I*, and all progeny will be light. Thus, rare plants with *B-I* pigmentation levels may indicate the presence of a dominant mutation preventing paramutation. Presence of the recessive g*l2* and *wt* markers on the *B-I* chromosome enabled rapid identification of self-pollination contaminant offspring. Pollen from the exceptional dark plant (KK1238-1) was crossed onto *gl2 B' wt* ears (Figure S1). The *mop2-2* mutation was isolated in an independent EMS screen in which *B-I* pollen was treated with EMS [74] and placed on silks of *B'* plants. Resulting F_1_ plants were screened for dominant mutation phenotypes (none found) and self pollinated, and F_2_ progeny were screened for dark plants.

### Mapping of the *Mop2-1* mutation

Initial linkage of *Mop2-1* relative to the *b1*, *gl2* and *wt* loci was noticed in the test cross of the original *Mop2-1* dark plant with *+ gl2 B' wt* tester (Figure S1). Of the 15 progeny plants, 12 plants inherited parental 2S chromosomes from the original mutant plant, three plants exhibited phenotypes consistent with recombination, two between *Mop2-1* and *Gl2*, and one between B-I* and *wt* (Figure S1). This result indicated that the *Mop2-1* mutation is distinct from *b1* and located distal of *Gl2*. The map position of the *Mop2-1* mutation was subsequently confirmed and refined using simple sequence repeat (SSR) molecular markers (www.maizegdb.org) and a larger number of plants (data not shown). Two markers tightly linked to *Mop2-1*, bnlg1117 and bnlg1338, were used to routinely follow *Mop2-1* segregation prior to cloning. PCR products were resolved using 4% Super Fine Resolution agarose gels. To further refine the location of *Mop2*, a large mapping population was generated by crossing homozygous *Mop2-1* plants with B73, the inbred sequenced for the maize genome project that is also highly polymorphic relative to the *Mop2-1* stock. The F_1_ was then backcrossed to homozygous *Mop2-1*, the resulting seed planted, and DNA was extracted from 1308 dark plants, the phenotype expected for homozygous *Mop2-1*. The resulting samples were screened with polymorphic markers on chromosome 2S (available upon request) to first determine the boundaries of the *Mop2-1* interval (13 BACs) and then to further map it to within a two BAC interval on FPC contig 69 (Figure 2).

### Bioinformatics to identify candidate genes

Examination of gene models within the two BAC interval (NCBI accessions AC1911113.2 and AC213986.2) revealed the presence of 21 putative protein encoding genes (Release 3b.50, February, 2009), including the AC191113.2_FGT037 gene model, which is predicted to encode the second largest subunit of a RNA polymerase most similar to rice gene SJNBa0063C18.1 (*OsNrpd2a*). The gene model AC191113.2_FGT037 was refined using FGENESH+ and Arabidopsis *NRPD2/NRPE2* and *OsNrpd2a* genes as guides. The refined model corresponded well to the *NRPDB101* gene model from www.chromdb.org, and was experimentally verified by PCR amplification of the full length transcript and sequencing (NCBI GQ453405). The corrected AC191113.2_FGT037 gene model was renamed *nrpd2/e2a*. Please note that the order of fragments within the AC191113 in Figure 2 is different from that shown www.maizesequence.org at the time of publication. We reordered fragments based on additional sequence information that indicated positions of overlapping fragments within the AC191113 and neighboring BACs (not shown).

### Sequencing of the candidate gene in *Mop2-1* and *mop2-2* stocks

A custom BAC library was constructed from *Mop2-1* genomic DNA with the assistance of the Arizona Genomic Institute (Tucson, AZ). Nylon filters with printed DNA from this library were hybridized with a probe unique to the 3' UTR of *nrpd2/e2a* (available upon request). One of the positive clones that contained the full length *nrpd2/e2a* gene was used as a template to PCR amplify all predicted exons (primer sequences available upon request). The resulting PCR fragments were sequenced in both directions using the core sequencing facility at University of Arizona (Tucson, AZ). Consistent with an EMS-induced mutation, a G to A transition was identified in the *nrpd2/e2a* gene, within an absolutely conserved motif in exon 7. To identify additional mutations, we sequenced the exons of the *nrpd2/e2a* gene in three newly isolated EMS-induced *b1* paramutation mutants. Exons of the *nrpd2/e2a* gene were PCR amplified and amplicons were sequenced in both directions for each candidate mutant. One of the new mutants was found to carry a G to A transition in a conserved domain in exon 6, consistent with an EMS induced mutation. This mutation was named *mop2-2*. The *nrpd2/e2a* gene was sequenced in a total of 12 dark plants (*mop2-2* homozygotes) and all carried the same lesion, indicating that the lesion segregated with the mutant paramutation phenotype.

### Sequence alignment and phylogenetic analysis

Sequence alignment was carried out using MUSCLE [75], manually edited in GENEDOC 2.6.04. Phylogenetic and molecular evolutionary analyses were conducted using *MEGA* version 4 software [76]. Bootstrap neighbor-joining method with 1000 replicates was used to generate the phylogenic trees. Protein sequences of the maize second largest subunits of Pol-I (*ZmNRPA2*) and Pol-II (*ZmNRPB2a*, *ZmNRPB2b*) were predicted using FGENESH+ software and corresponding Arabidopsis proteins as guides (Figure S7). To obtain genomic sequence suitable for protein prediction of maize *nrpd2/e2b*, the gap in the AC212557 sequence was PCR amplified and sequenced. FGENESH+ (http://linux1.softberry.com/berry.phtml) was used to generate the gene model, which is equivalent to GRMZM2G146935_T02 at www.maizesequence.org. The maize *nrpd2/e2c* gene model was predicted from the AC203335.4 BAC sequence using FGENESH+ and rice *OsNrpd2b* as a guide, which was equivalent to GRMZM2G133512_T01 at www.maizesequence.org. The quality of the resulting gene models was inspected using the ClustalX multiple sequence alignment program [77]. For the alignment shown in Figure S3 and the phylogenetic tree shown in Figure 3C and 3D, sequences of the second largest subunits were provided by the Pikaard lab or retrieved from NCBI. The complete list of the genes used for the phylogenetic analysis is in Table S1.

### RT–PCR analysis of the maize *nrpd2/e2* genes

For expression analysis of the maize *nrpd2/e2* genes total RNA was extracted from tissues flash frozen in liquid nitrogen using the Trizol protocol as described by manufacturer (Invitrogen). Total RNA was treated with DNAseI (Invitrogen) and acid phenol (Ambion) to remove DNA contamination. First stand synthesis was carried out using oligo(dT) primers and 10 ug of total RNA. Superscript III First Strand Synthesis System (Invitrogen) was used according to manufacturer's recommendations at 55 C for 1 hour. About 200 ng of cDNA were used for each quantitative PCR assay on the Bio-Rad MyIQ Real-Time PCR machine and quantified using My**-**IQ software (Bio-Rad). Expression of *nrpd2/e2* genes was normalized to *actin1* expression. Sequences of gene-specific primers are available upon request.

All plant materials were from the B73 inbred with the exception of the Black Mexican Sweet (BMS) tissue culture cells and the HiII callus cells. All tissues were collected in the morning, 2–4 hours after sunrise. At the time of collection all plant tissues were immediately frozen in liquid nitrogen. Seedlings were germinated for 6 days after imbibing in paper towels at 16 day/8 night photo period at 20C and tissue before the first leaf emerged from the coleoptile. Root tissue was from seedlings grown in a vermiculite/soil mixture, 10 days after emergence (2–3 leaf stage). Seedling roots were washed before freezing. Husk, silks, immature ears, immature tassels, and pollen were collected from field grown plants. Husk and silks were collected simultaneously on the first day of silk emergence. Immature ears and tassels were collected when they were 1.5 cm to 2.5 cm in length. Pollen was collected in the morning from tassels on the first day of shedding. Pollen was filtered through fine metal mesh filters to remove debris before freezing. Endosperm and embryos were collected from greenhouse grown plants at 12 and 14 days after pollination (DAP). HiIIAxB type II embryogenic callus was maintained on N6 media with 1.0 mg/L of 2, 4-dichlorophenoxyacetic acid with sub-culturing every two weeks as described [78]. The BMS callus suspension culture used in these experiments was acquired from C. Armstrong (Monsanto Company, St. Louis, MO) in 2001. BMS cultures were maintained in N6 media supplemented with 1.5 mg/L of 2, 4-dichlorophenoxyacetic acid with monthly subcultures to a fresh media. Both the HiII and BMS cultures were cultured in the dark at 26–28°C.

### *b1* and *pl1* genetic stocks

The *gl2 b wt*, *Pl*, *r-g* (inbred K55 background), the *B-I Pl r-g* (inbred W23 background) and the *B' Pl r-g* (inbred K55 background) stocks were originally obtained from E.H. Coe, Jr. (University of Missouri, Columbia). Paramutagenic *Pl'* allele and paramutable *Pl-Rhoades* (*Pl-Rh*) were previously described [3].

### *r1* genetic stocks and tests with *Mop2-1*

The phenotypes and paramutation properties of the *R-st*, *R-r* and *r* alleles were previously described [79],[80]. Paramutation at *r1* is typically assayed in the aleurone, the outer cell layer of the endosperm, where purple anthocyanin pigments accumulate in the highly expressed *R-r* allele; reduced pigmentation is observed when *R-r* is paramutated to *R-r'* by *R-st*. The fully colored *R-sc* allele [79],[81] was used as a positive seed color control (Figure 6C). Both heterozygous and homozygous *Mop2-1* plants were assayed for effects on *r1* paramutation (Figure S5), although only a small number of *Mop2-1* homozygotes could be assayed because these plants have reduced fertility. To quantify changes in seed color occurring during paramutation, the relative color was determined as described [82].

### *p1* genetic stocks and tests with *Mop2-1*

The paramutable *P1-rr* stock, the standard *P1-rr4B2* allele of the *p1* gene [83], has red pericarp and red cob pigmentation, while the silenced *P1-rr'* allele has lightly patterned or colorless pericarp and pink cob pigment [4]. The highly paramutagenic *P1-rr'/P1-rr'*; *P1.2b::GUS/-* stock was used to assess *Mop2-1* effects on *p1* paramutation, by crossing with a *Mop2-1 B' P1-rr* stock. The P1.2b::GUS transgene (transgenic event P2P147-37) carried the P1.2 enhancer fragment that is sufficient for paramutation, fused to the basal *P1-rr* promoter, the *Adh1* (maize *Alcohol dehydrogenase1* gene intron 1), the *E.coli* GUS gene and the *PinII* (potato *Proteinase InhibitorII*) 3', along with a resistance gene for the BASTA herbicide [13]. Details of the crosses are presented in Figure S4.

### Genetic and molecular tests with Ms45Δ1pIR and 5126pIR transgenes

The Ms45Δ1pIR and 5126pIR transgenic constructs and phenotypes upon silencing were previously described [67]. To assay whether the *Mop2-1* mutation could disrupt pIR transgene induced silencing of the endogenous *Ms45* and *5126* genes, *Mop2-1* plants were crossed with four independent transgenic events each for Ms45Δ1pIR and 5126pIR. Herbicide resistant F_1_ plants were backcrossed to the *Mop2-1* stock to generate a family segregating heterozygous or homozygous *Mop2-1* plants. These were grown in the field and sprayed with herbicide to remove non transgenic plants, while the remaining transgenic plants were visually scored for plant color and male fertility at anthesis. Plants homozygous for the *Mop2-1* mutations were initially identified using the dark plant color associated with increased expression of the *B'* allele. For a subset of the Ms45Δ1pIR plants, the presence of the *Mop2-1* mutation was confirmed by genotyping with molecular markers. Northern blot analysis of *Ms45* and *5126* transcript levels were as previously described [67],[84]. Conditions for small RNA Northern blots were the same as described below, using previously described probes for MS45 and 5126 transgene siRNA detection [67].

### Northern blot analysis of siRNAs

RNA was extracted from 3 g of immature ears (3–5 cm long) using TRIZOL reagent (Invitrogen), and the large RNA fraction was precipitated using 5% polyethyleneglycol MW 8000 [85]. The aqueous phase, enriched for the small RNA fraction, was subjected to phenol:chlorophorm:isoamyl-alcohol (24∶1∶1) extraction followed by ethanol precipitation. The pellet was resuspended in DEPC treated water. Approximately 100 ug of the small RNA fraction was loaded in each lane. RNA was electrophoresed on 15% denaturing UREA polyacrylamide gels, electroblotted onto GeneScreen Nylon membrane and immobilized using UV crosslinking. Blots were hybridized with a ^32^P end labeled DNA:LNA (DNA::Locking Nucleic Acid) oligonucleotide [86]. The DNA::LNA oligo (vc1658F, TGAA+CATCTT+GTCCA+GTTAAAT+CACTGG+ACACC+GTGAC+AGCC+ACA; “+” precedes an LNA base) was synthesized by Sigma-Proligo. For the U6 probe, DNA oligo (vc1969F, AGACATCCGATAAAATTGGAACGATACAGA) was end labeled with ^32^P. Hybridization and image was processed using QuantityOne software (BioRad).

### Nuclear run-on analysis

Approximately ∼5 g of immature ears were used to extract nuclei as described [20]. The nuclei isolations and run on reactions were as described [22]. To prepare the *b1* tandem repeat probes, PCR fragments carrying T3 promoter tails were used as templates for *in vitro* transcription with T3 RNA polymerase (Invitrogen), as recommended by the manufacturer. Sequences of the primers used to produce the *b1* RNA probes are available upon request. The positive control, the *Ubiqutin2* RNA probe, was as described [27]. Lambda phage genomic DNA, 100 ng per slot, was used as a negative control.

## Supporting Information

Figure S1 Genetic mapping of the *Mop2-1* mutation using phenotypic markers linked to the *b1* locus on chromosome 2S. Asterisk denotes the *B-I* that was protected from paramutation in *Mop2-1/+* plants. Red bars indicate the interval in which recombination occured in the previous generation. In testcross 1, 12 out of 15 progeny plants inherited parental combinations of phenotypic markers on chromosome 2. Analysis of phenotypes of the three recombinant plants indicates that *Mop2-1* is located distal to the *gl2* locus. Testcross 2 was carried out to score the presence of the *Mop2-1* mutation.
(0.10 MB PDF)

Figure S2 Crossing schema for the genetic test used to assay *Mop2-1/+* effects on preventing *b1* paramutation and relief of *B'* silencing. The *B-I* allele exposed to homozygous *Mop2-1* is denoted by an asterisk. The red bar indicates the potential for recombination as the *b1* and *Mop2-1* loci are linked (27 cM). The *B-Peru (B-P)* allele of the *b1* gene does not undergo paramutation. Weak plant pigment specified by *B-P* is convenient for observing *B'* and B-I* phenotypes. Because *B'* and *B-I* do not pigment seeds, the purple seed color specified by *B-P* is used for pre-planting segregation of B'/B-I* and *B'/B-P* seeds. If B-I* escaped paramutation in the previous generation, then accounting for the linkage between *b1* and *mop2*, assuming absence of spontaneous paramutation of B-I* to *B'*, and 100% penetrance of the *Mop2-1* mutation, 73% of dark + B-I*/+ *B-P* and 27% of light + *B'/+ B-P* progeny are expected.
(0.10 MB PDF)

Figure S3 Alignment of second largest subunits of RNA polymerases. Alignment was performed using MUSCLE, edited using GENEDOC, and shaded using BOXSHADE. Identical amino acids are shaded in black, while similar amino acids are shaded in gray. Conserved domains are underlined and indicated A though I [72]. The active site (metal B) is indicated by asterisks [72]. Positions of *Mop2-1* and *mop2-2* mutations are indicated above the alignment in blue. Positions of removed amino acids are indicated above the alignment in gray.
(0.33 MB PDF)

Figure S4 Crossing schema for experiment to test effect of *Mop2-1* on *p1* paramutation. The paramutagenic *P1-rr'* allele has light patterned pericarp, while paramutable *P1-rr* has red pericarp pigment. The P1.2b::GUS transgene carried the highly paramutagenic P2P147-37 integration event [13]. To assay whether the *Mop2-1* mutation would prevent *p1* paramutation, plants carrying the paramutagenic endogenous *P1-rr'* allele and the P1.2b::GUS transgene were pollinated with the *Mop2-1 P1-rr* stock. Because the *B'* allele was introduced together with the *Mop2-1* mutation we used dark plant pigment for initial identification of *Mop2-1* homozygous plants in segregating families, and subsequent molecular markers were used to verify the *Mop2-1/Mop2-1* and *Mop2-1/+* genotypes. Spraying with the BASTA herbicide eliminated non transgenic plants. In the F_1_ all transgenic plants had light pericarp color indicating that when *Mop2-1* is heterozygous it does not *p1* prevent paramutation. A backcross with the *Mop2-1* stock was used to generate families in which the effect of homozygous *Mop2-1* on preventing *p1* paramutation was assayed. Results are presented in Table 2.
(0.17 MB PDF)

Figure S5 Schematic drawing of genetic experiment that tests *Mop2-1* effect on preventing *r1* paramutation. Plants heterozygous for *Mop2-1/+* and carrying *R-st/r* or *R-r/r* were crossed to produce F_1_ plants. Although *r1* paramutation occurs in the F_1_, observation of paramutation requires a testcross to a colorless allele that does not participate in paramutation (*r*) to obtain seeds in which pigment levels reveal the extent of paramutation of *R-r* to *R-r'* [81]. To produce testcross progeny, mottled and fully colored F_1_ seeds (*R-st/R-r'* or *r/R-r*) were planted. Resulting plants were genotyped for the *Mop2-1* mutation and out crossed onto silks carrying the *r* allele. Seeds resulting from the test cross were sorted to identify mottled and/or full colored seeds and light reflectance was measured to determine the relative color of the seeds. Data summarized in Figure 6C.
(0.10 MB PDF)

Figure S6 Schematic drawing of genetic experiment that tests *Mop2-1* effect on Ubi::MS45pIR and Ubi::5126pIR transgene induced silencing. pIR is used to symbolize the inverted repeat transgenes. Dark plant pigmentation specified by *B'* in *Mop2-1/Mop2-1* plants was used to classify progeny. Molecular genotyping of a subset of plants revealed close correspondence between the *B'* phenotype and the *Mop2-1* genotype; 28/33 dark plants were homozygous and 28/30 light plants were heterozygous for *Mop2-1*. Recombination between the linked *b1* and *mop2* loci does not influence outcome of this experiment because at least one *B'* allele is present to report the *Mop2-1* genotype.
(0.16 MB PDF)

Figure S7 Protein models of maize second largest subunits of Pol-I and Pol-II used for phylogenetic analysis. Protein sequences of the maize second largest subunits of Pol-I (ZmNRPA2) and Pol-II (ZmNRPB2a, ZmNRPB2b) were predicted using FGENESH+ (http://linux1.softberry.com/) software and the corresponding Arabidopsis proteins as guides.
(0.04 MB DOC)

Table S1 Information for DNA dependent RNA polymerase second-largest subunits used for phylogenetic analysis.
(0.02 MB PDF)
