# Supplementary material for: A Dominant Mutation in mediator of paramutation2, One of Three Second-Largest Subunits of a Plant-Specific RNA Polymerase, Disrupts Multiple siRNA Silencing Processes
Source: PLoS Genet. 2009 Nov 20;5(11):e1000725. doi: 10.1371/journal.pgen.1000725 (PMC2774164; doi:10.1371/journal.pgen.1000725)
Supplement: Figure S1 — Genetic mapping of the Mop2-1 mutation using phenotypic markers linked to the b1 locus on chromosome 2S. Asterisk denotes the B-I that was protected from paramutation in Mop2-1/+ plants. Red bars indicate the interval in which recombination occured in the previous generation. In testcross 1, 12 out of 15 progeny plants inherited parental combinations of phenotypic markers on chromosome 2. Analysis of phenotypes of the three recombinant plants indicates that Mop2-1 is located distal to the gl2 locus. Testcross 2 was carried out to score the presence of the Mop2-1 mutation. (0.10 MB PDF) [file pgen.1000725.s001.pdf]

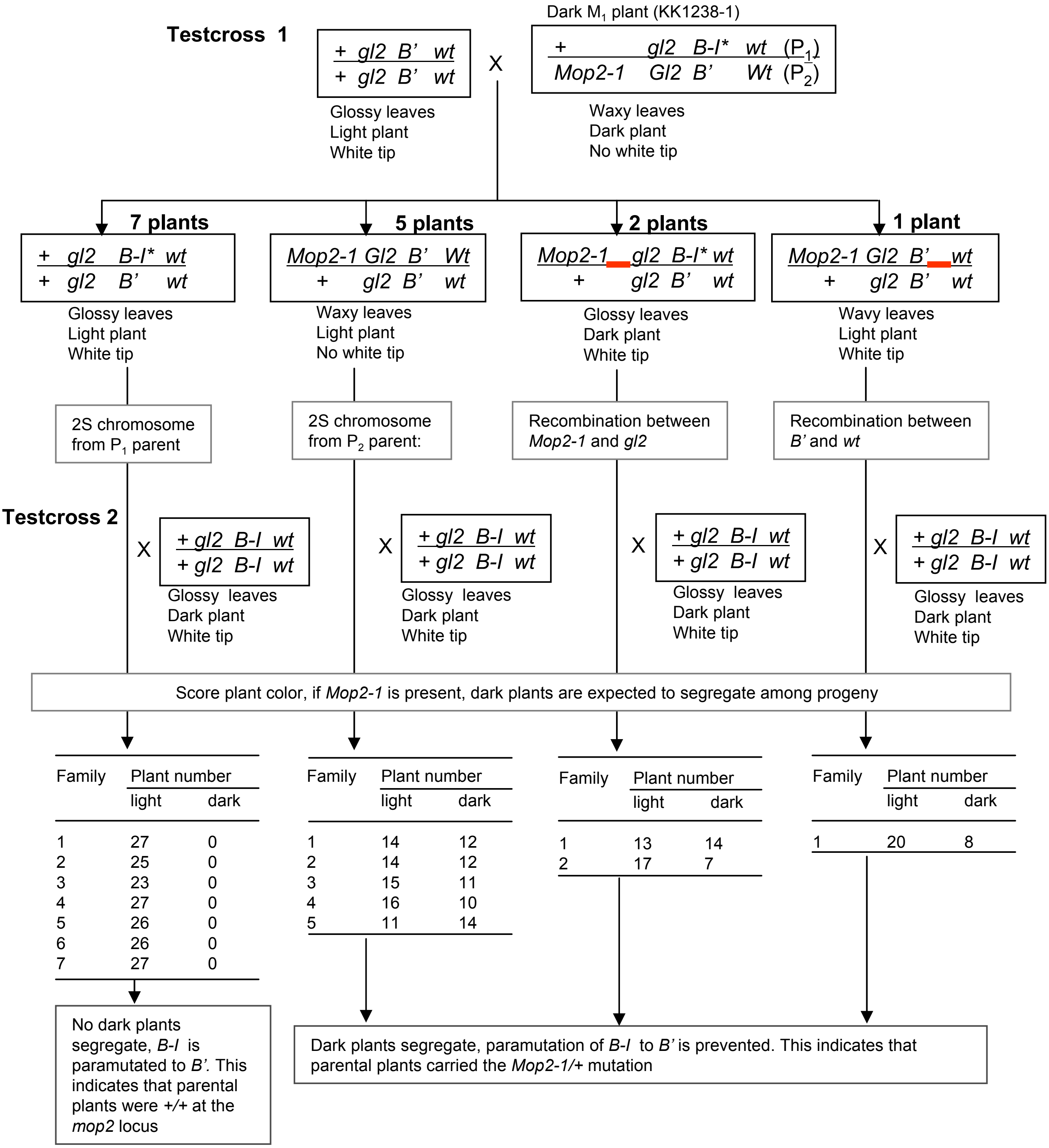


**Figure S1. Genetic mapping of the *Mop2-1* mutation using phenotypic markers linked to the *b1* locus on chromosome 2S.** Asterisk denotes the *B-I* allele that was protected from paramutation in *Mop2-1/+* plants. Red bars indicate the interval in which recombination occurred in the previous generation. In testcross 1, 12 out of 15 progeny plants inherited parental combinations of phenotypic markers on chromosome 2. Analysis of phenotypes of the three recombinant plants indicates that *Mop2-1* is located distal to the *gl2* locus. Testcross 2 was carried out to score the presence of the *Mop2-1* mutation.
