# Supplementary material for: A Dominant Mutation in mediator of paramutation2, One of Three Second-Largest Subunits of a Plant-Specific RNA Polymerase, Disrupts Multiple siRNA Silencing Processes
Source: PLoS Genet. 2009 Nov 20;5(11):e1000725. doi: 10.1371/journal.pgen.1000725 (PMC2774164; doi:10.1371/journal.pgen.1000725)
Supplement: Figure S2 — Crossing schema for the genetic test used to assay Mop2-1/+ effects on preventing b1 paramutation and relief of B' silencing. The B-I allele exposed to homozygous Mop2-1 is denoted by an asterisk. The red bar indicates the potential for recombination as the b1 and Mop2-1 loci are linked (27 cM). The B-Peru (B-P) allele of the b1 gene does not undergo paramutation. Weak plant pigment specified by B-P is convenient for observing B' and B-I* phenotypes. Because B' and B-I do not pigment seeds, the purple seed color specified by B-P is used for pre-planting segregation of B'/B-I* and B'/B-P seeds. If B-I* escaped paramutation in the previous generation, then accounting for the linkage between b1 and mop2, assuming absence of spontaneous paramutation of B-I* to B', and 100% penetrance of the Mop2-1 mutation, 73% of dark + B-I*/+ B-P and 27% of light + B'/+ B-P progeny are expected. (0.10 MB PDF) [file pgen.1000725.s002.pdf]

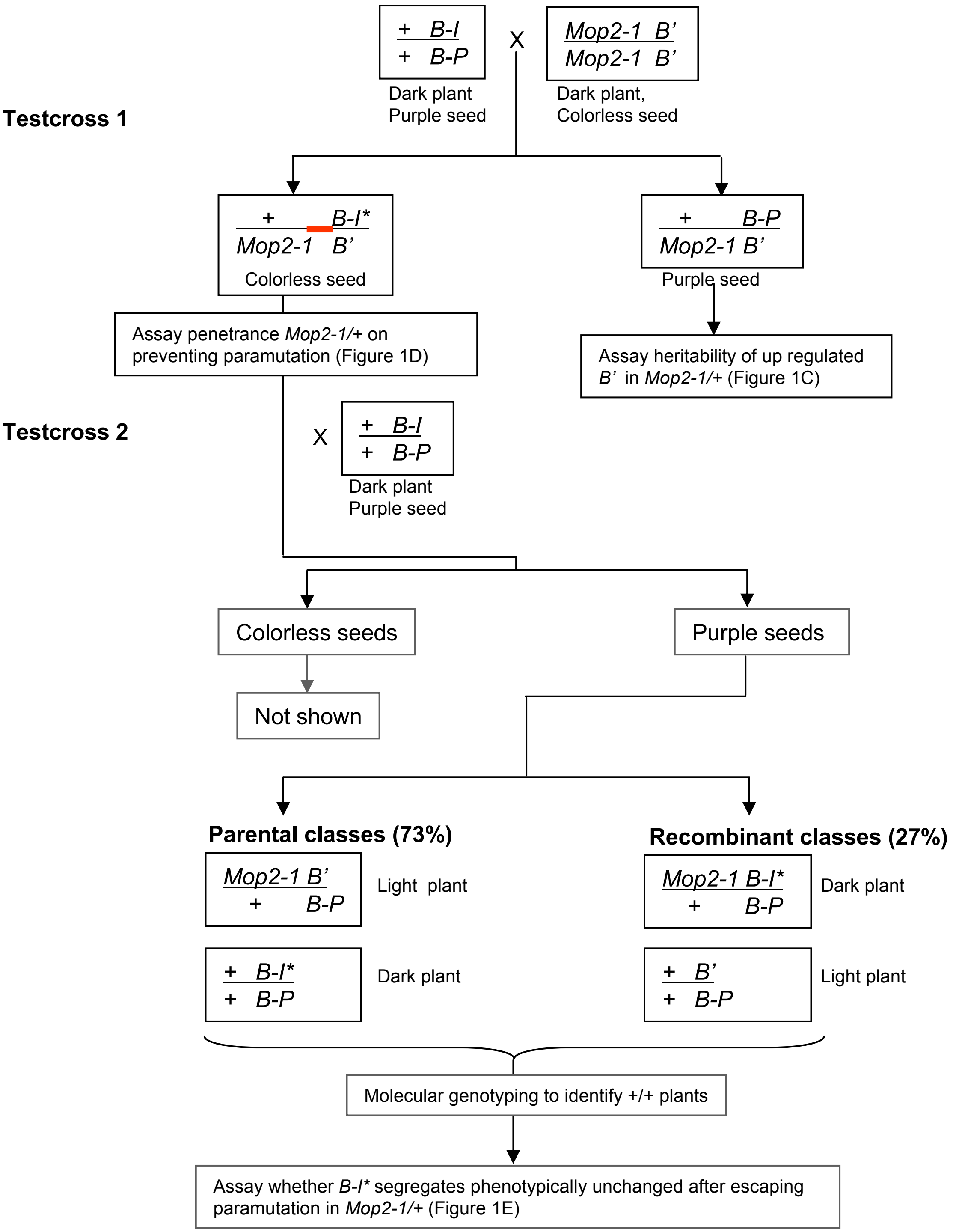


**Figure S2. Crossing schema for the genetic test used to assay *Mop2-1/+* effects on preventing *b1* paramutation and relief of *B'* silencing.** The *B-I* allele exposed to homozygous *Mop2-1* is denoted by an asterisk. The red bar indicates the potential for recombination as the *b1* and *Mop2-1* loci are linked (27cM). The *B-Peru* (*B-P*) allele of the *b1* gene does not undergo paramutation. Weak plant pigment specified by *B-P* is convenient for observing *B'* and *B-I** phenotypes. Because *B'* and *B-I* do not pigment seeds, the purple seed color specified by *B-P* is used for pre-planting segregation of *B'/B-I** and *B'/B-P* seeds. If *B-I** escaped paramutation in the previous generation, then accounting for the linkage between *b1* and *mop2*, assuming absence of spontaneous paramutation of *B-I** to *B',* and 100% penetrance of the *Mop2-1* mutation, 73% of dark + *B-I*/+ B-P* and 27% of light + *B'/+ B-P* progeny are expected.
