# Supplementary material for: A Dominant Mutation in mediator of paramutation2, One of Three Second-Largest Subunits of a Plant-Specific RNA Polymerase, Disrupts Multiple siRNA Silencing Processes
Source: PLoS Genet. 2009 Nov 20;5(11):e1000725. doi: 10.1371/journal.pgen.1000725 (PMC2774164; doi:10.1371/journal.pgen.1000725)
Supplement: Figure S3 — Alignment of second largest subunits of RNA polymerases. Alignment was performed using MUSCLE, edited using GENEDOC, and shaded using BOXSHADE. Identical amino acids are shaded in black, while similar amino acids are shaded in gray. Conserved domains are underlined and indicated A though I [72]. The active site (metal B) is indicated by asterisks [72]. Positions of Mop2-1 and mop2-2 mutations are indicated above the alignment in blue. Positions of removed amino acids are indicated above the alignment in gray. (0.33 MB PDF) [file pgen.1000725.s003.pdf]

Figure S3. Alignment of Second Largest Subunits of RNA Polymerases.

```
EcRpoB        1 ------------------------------------------------------------
ScRPB2        1 ------------------------------------------------------------
AtNRPA2       1 ------------------------------------------------------------
OsNRPA2       1 ------------------------------------------------------------
AtNRPC2       1 -----------------------------------------------------------M
OsNRPC2       1 -----------------------------------MEKVRVLFYRTIAPQHPSSLHRTEE
AtNRPB2       1 --------------MEYNEYE---------------------------------------
OsNRPB2       1 --------------MEDDEYEEGMEMEMGGHHHPHHGG--GYGAEE-----Y--GAVGGE
ZmNRPB2a      1 --------------MEDDEYEDGMDMGYGGHHQ-RGGGHAGYGAEEDDEMGYGEGGGDGD
ZmNRPB2b      1 --------------MEDDEYEEGMEMGYGGHHQ-HGGGHAGYGAEEDDEVGY--GGGGGE
AtNRPD2a      1 --------------------------------------------------------MPDM
AtNRPD2b      1 ------------------------------------------------------------
OsNRPDa       1 -----------------------------------------------MDVDRGCHSMDTT
ZmNRPD2/E2a   1 MEEPQKDSGQPSKSSDSELEAMVLDDNGAGKSHSMEENRDSPIDVDGGQSSMDVDIKGKS
ZmNRPD2/E2b   1 MEELQKDSALPSNSSDSEPEAMELDDNGAGKSHSIEGNRDSPIDTDEGQPSMDVDIKGKS
OsNRPD2b      1 ---------------------------------------------------MDVDL----
ZmNRPD2/E2c   1 MGKPQTDGDQPVNSDTDVMDCLNFDGHGDVEDEVAKGEQQPSGEVDEQQSVMDVDLTCIT
                           |ZmNRPD2/E2c                 |ZmNRPD2/E2c
                           1 aa deleted                 3 aa deleted

EcRpoB        1 -----------------MVYSYTEKKRIRKDFGKRPQVLDVPYLLSIQLDSFQKFIEQDP
ScRPB2        1 -MSDLANSEKYYDEDPYGFEDESAPITAEDSWAVISAFFREKGLVSQQLDSFNQFVDYTL
AtNRPA2       1 ----------------MVVNAKDSTVPTMEDFK------ELHNLVTHHIESFDYMTLKG-
OsNRPA2       1 ------------------------------------------------------------
AtNRPC2       2 GLDQEDLDLTNDDHFIDKEKLSAPIKSTADKFQLVPEFLKVRGLVKQHLDSFNYFINVGI
OsNRPC2      26 EEAPAAKDPNASLPSPTYRSLAAPVTKPVDKFALLPAFLKVRGLVKEHIDSFNYFITKGI
AtNRPB2       8 ---PEPQYVED-----DDDEEIT----QEDAWAVISAYFEEKGLVRQQLDSFDEFIQNTM
OsNRPB2      38 EMEDEEADGDA-----PDEEEIT----QEDAWAVISAYFEEKGLVRQQLDSFDEFIQNTM
ZmNRPB2a     46 EM-EEEADGDA-----EQQEDIT----QDDAWAVISAYFEEKGLVRQQLDSFDEFIQNTM
ZmNRPB2b     44 EM-DEDADGDA-----EQQEDIT----QDDAWAVISAYFEEKGLVRQQLDSFDEFIQNTM
AtNRPD2a      5 DIDVKDLEEFEATTGEINLSELGEGFLQSFCKKAATSFFDKYGLISHQLNSYNYFIEHGL
AtNRPD2b      1 ------------------------------------------------------------
OsNRPD2a     14 RSSLGDDGKGKRDSYAQIPVDMSIPSLEKFCKEASRSFFDEIGLISHQINSYNEFVSHGL
ZmNRPD2/E2a  61 SLSDDVNGKSSSEPYSNAPIDMSVESLEKFCKEASRSFFDEVGLISHQINSYNEFVSHGL
ZmNRPD2/E2b  61 SLNDDVNGKSSSEPFSNPPINMSVESLEKFCKEASRSFFDEVGLISHQINSYNEFVSHGL
OsNRPD2b      6 RVIPSLKDGGHADPPVQVPVDKRIASLEKLCKEASRSFFRETRLVSHQINSYNDFVSHGL
ZmNRPD2/E2c  65 SVGDEGNGKASSDLPSQVPVDFNVASLEKFCKEAARSFFSETGLVSHQINSYDHFVSHGL
                       |ZmNRPD2/E2c
                       21 aa deleted

EcRpoB       44 EGQYGLEAAFRSVFPIQSYS--------GNSELQYVSYRLGEPVFD--------------
ScRPB2       60 QDIICEDSTLILEQLAQHTT--ESDNISRKYEISFCKIYVTKPMVNESDGVTH----ALY
AtNRPA2      38 ------------------------------------------------------------
OsNRPA2       1 ------------------------------------------------------------
AtNRPC2      62 HKIVKANSRITST-------------VDPSIYLRFKKVRVGEPSIINVN--TVE---NIN
OsNRPC2      86 RNIVKANNRIEAR-------------NNPSIFLRYNSVRVGVPSVQVQY--IAE---KIT
AtNRPB2      56 QEIVDESADIEIRPESQHNPGHQSDFAETIYKISFCQIYLSKPMMTES---DGE-TATLF
OsNRPB2      89 QEIVDESADIEIRPESQHNPGRQAEFAETLHKISFCQIYLSKPMMTEA---DGE-TATLF
ZmNRPB2a     96 QEIVDESADIEIRPESQHNPGRQAEFAETLHKISFCQIYLSKPMMTEA---DGE-TATLF
ZmNRPB2b     94 QEIVDESADIEIRPESQHNPGRQAEFAETLHRISFCQIYLSKPMMTEA---DGE-TATLF
AtNRPD2a     65 QNVFQSFGEMLVE--PSFDVVKKKDNDWRYATVKFGEVTVEKPTFFSD---DKE--LEFL
AtNRPD2b      1 ------------------------------------------------------------
OsNRPD2a     74 QELFDSLGEVTVE--PSYDPSNRGPGGWRHAIIKFGRVQLEEPVFWSHGCDIDEQSLKLK
ZmNRPD2/E2a 121 QELFDSLGEVIVE--PGYDPSKKGSGGWKHAIIKFGRVKLEKPVFWTG---KDEGSVDFK
ZmNRPD2/E2b 121 QELFDSLGEVIVE--PGYDPSKKGSGSWKHAIIKFGRVKLEKPVFWTG---KDEVSVDFK
OsNRPD2b     66 QKMFDSLDEVTVE---PDYDPSKKVGPWRHATIKFGRVELVYSLH---------------
ZmNRPD2/E2c 146 QELFDSLGEITVE---PDYDPSNKHGAWKHATVKFGRVKLDEPVFMLENSDLEEQDLKFK
```

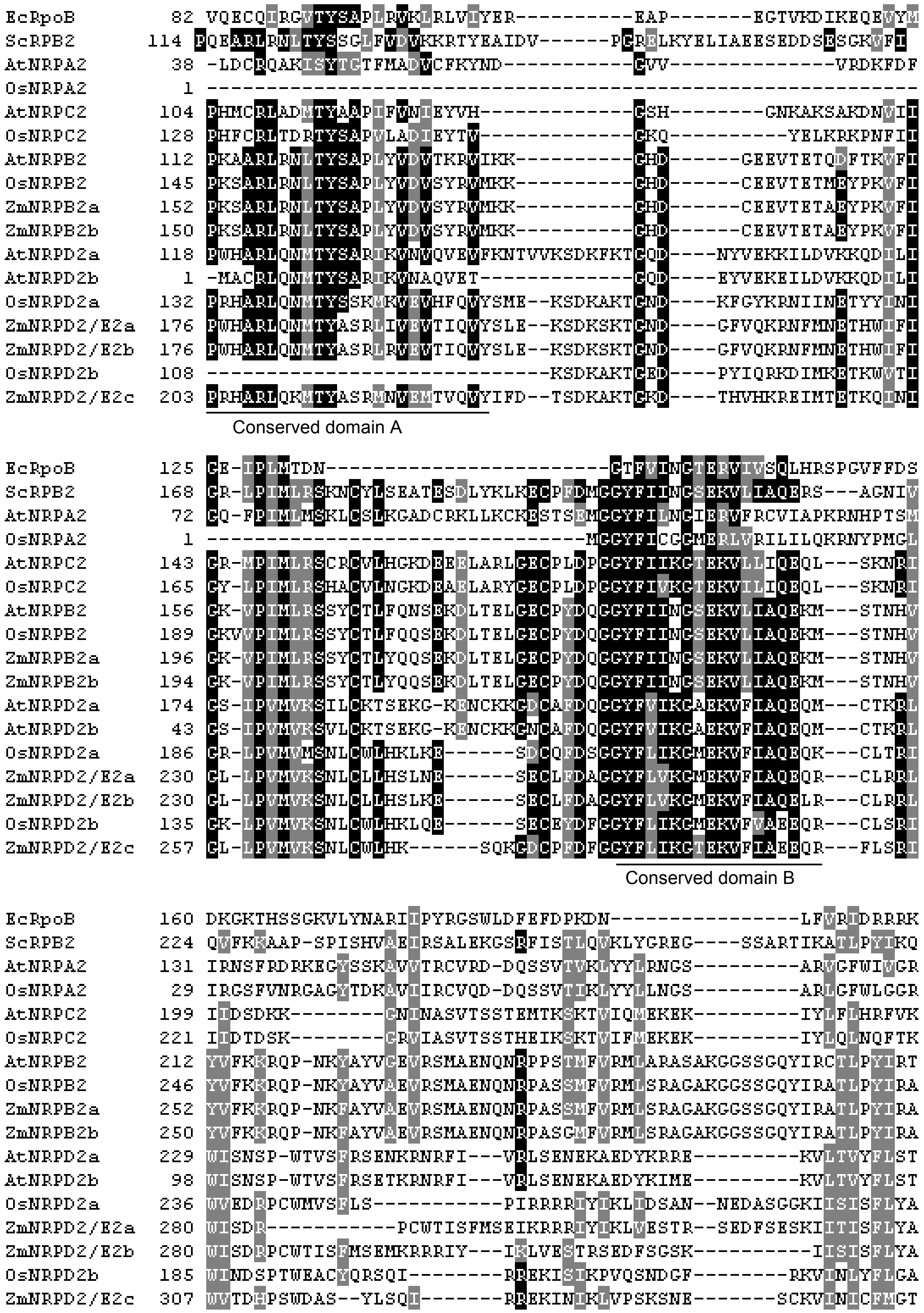
EcRpoB 82 VQECQIRGVTYSAPLRVKLRLVIYER----------EAP-------EGTVKDIKEQEVYM
ScRPB2 114 PQEARLRNLTYSSGLFVDVKKRTYEAIDV------PGRELKYELIAEESEDDSESGKVFI
AtNRPA2 38 -LDCRQAKISYTGTFMADVCFKYND-----------GVV--------------VRDKFDF
OsNRPA2 1 --
AtNRPC2 104 PHMCRLADMTYAAPIFVNIEYVH-------------GSH--------GNKAKSAKDNVII
OsNRPC2 128 PHFCRLTDRTYSAPVLADIEYTV-------------GKQ----------YELKRKPNFII
AtNRPB2 112 PKAARLRNLTYSAPLYVDVTKRVIKK----------GHD------GEEVTETQDFTKVFI
OsNRPB2 145 PKSARLRNLTYSAPLYVDVSYRVMKK----------GHD------CEEVTETMEYPKVFI
ZmNRPB2a 152 PKSARLRNLTYSAPLYVDVSYRVMKK----------GHD------CEEVTETAEYPKVFI
ZmNRPB2b 150 PKSARLRNLTYSAPLYVDVSYRVMKK----------GHD------CEEVTETAEYPKVFI
AtNRPD2a 118 PWHARLQNMTYSARIKVNVQVEVFKNTVVKSDKFKTGQD----NYVEKKILDVKKQDILI
AtNRPD2b 1 -MACRLQNMTYSARIKVNAQVET-------------GQD----EYVEKEILDVKKQDILI
OsNRPD2a 132 PRHARLQNMTYSSKMKVEVHFQVYSME--KSDKAKTGND----KFGYKRNIINETYYINI
ZmNRPD2/E2a 176 PWHARLQNMTYASRLIVEVTIQVYSLE--KSDKSKTGND----GFVQKRNFMNETHWIFI
ZmNRPD2/E2b 176 PWHARLQNMTYASRLRVEVTIQVYSLE--KSDKSKTGND----GFVQKRNFMNETHWIFI
OsNRPD2b 108 ----------------------------KSDKAKTGED----PYIQRKDIMKETKWVTI
ZmNRPD2/E2c 203 PRHARLQKMTYASRMNVEMTVQVYIFD--TSDKAKTGKD----THVHKREIMTETKQINI
Conserved domain A
EcRpoB 125 GE-IPLMTDN-------------------------GTFVINGTERVIVSQLHRSPGVFFDS
ScRPB2 168 GR-LPIMLRSKNCYLSEATESDLYKLKECPFDMGGYFIINGSEKVLIAQERS---AGNIV
AtNRPA2 72 GQ-FPIMLMSKLCSLKGADCRKLLKCKESTSEMGGYFILNGIERVFRCVIAPKRNHPTSM
OsNRPA2 1 ------------------------------MGGYFICGGMERLVRILILQKRNYPMGL
AtNRPC2 143 GR-MPIMLRSCRCVLHGKDEEELARLGECPLDPGGYFIIKGTEKVLLIQEQL---SKNRI
OsNRPC2 165 GY-LPIMLRSHACVLNGKDEAELARYGECPLDPGGYFIVKGTEKVILIQEQL---SKNRI
AtNRPB2 156 GK-VPIMLRSSYCTLFQNSEKDLTELGECPYDQGGYFIINGSEKVLIAQEKM---STNHV
OsNRPB2 189 GKVVPIMLRSSYCTLFQQSEKDLTELGECPYDQGGYFIINGSEKVLIAQEKM---STNHV
ZmNRPB2a 196 GK-VPIMLRSSYCTLYQQSEKDLTELGECPYDQGGYFIINGSEKVLIAQEKM---STNHV
ZmNRPB2b 194 GK-VPIMLRSSYCTLYQQSEKDLTELGECPYDQGGYFIINGSEKVLIAQEKM---STNHV
AtNRPD2a 174 GS-IPVMVKSILCKTSEKG-KENCKKGDCAFDQGGYFVIKGAEKVFIAQEQM---CTKRL
AtNRPD2b 43 GS-IPVMVKSVLCKTSEKG-KENCKKGNCAFDQGGYFVIKGAEKVFIAQEQM---CTKRL
OsNRPD2a 186 GR-LPVMVMSNLCWLHKLKE------SDCQFDSGGYFLIKGMEKVFIAQEQK---CLTRI
ZmNRPD2/E2a 230 GL-LPVMVKSNLCLLHSLNE------SECLFDAGGYFLVKGMEKVFIAQEQR---CLRRL
ZmNRPD2/E2b 230 GL-LPVMVKSNLCLLHSLKE------SECLFDAGGYFLVKGMEKVFIAQELR---CLRRL
OsNRPD2b 135 GK-LPVMVKSNLCWLHKLQE------SECEYDFGGYFLIKGMEKVFVAEEQR---CLSRI
ZmNRPD2/E2c 257 GL-LPVMVKSNLCWLHK------SQKGDCPFDFGGYFLIKGTEKVFIAEEQR---FLSRI
Conserved domain B
EcRpoB 160 DKGKTHSSGKVLYNARIIPYRGSWLDFEFDPKDN-----------------LFVRIDRRRK
ScRPB2 224 QVFKKAAP-SPISHVAEIRSALEKGSRFISTLQVKLYGREG----SSARTIKATLPYIKQ
AtNRPA2 131 IRNSFRDRKEGYSSKAVVTRCVRD-DQSSVTVKLYYLRNGS---------ARVGFWIVGR
OsNRPA2 29 IRGSFVNRGAGYTDKAVIIRCVQD-DQSSVTIKLYYLLNGS---------ARLGFWLGGR
AtNRPC2 199 IIDSDKK---------GNINASVTSSTEMTKSKTVIQMEKEK---------IYLFLHRFVK
OsNRPC2 221 IIDTDSK--------GRVIASVTSSTHEIKSKTVIFMEKEK---------IYLQLNQFTK
AtNRPB2 212 YVFKKRQP-NKYAYVCEVRSMAENQNRPPSTMFVRMLARASAKGGSSGQYIRCTLPYIRT
OsNRPB2 246 YVFKKRQP-NKYAYVAEVRSMAENQNRPASSMFVRMLSRAGAKGGSSGQYIRATLPYIRA
ZmNRPB2a 252 YVFKKRQP-NKFAYVAEVRSMAENQNRPASSMFVRMLSRAGAKGGSSGQYIRATLPYIRA
ZmNRPB2b 250 YVFKKRQP-NKFAYVAEVRSMAENQNRPASGMFVRMLSRAGAKGGSSGQYIRATLPYIRA
AtNRPD2a 229 WISNSP-WTVSFRSENKRNRFI---VRLSENEKAEDYKRRE---------KVLTVYFLST
AtNRPD2b 98 WISNSP-WTVSFRSETKRNRFI---VRLSENEKAEDYKIME---------KVLTVYFLST
OsNRPD2a 236 WVEDRPCWMVSFLS-----------PIRRRRIYIKLIDSAN--NEDASGGKIISISFLYA
ZmNRPD2/E2a 280 WISDR-----------PCWTISFMSEIKRRRIYIKLVESTR--SEDFSESKIITISFLYA
ZmNRPD2/E2b 280 WISDRPCWTISFMSEMKRRRIY---IKLVESTRSEDFSGSK----------IISISFLYA
OsNRPD2b 185 WINDSPTWEACYQRSQI---------RREKISIKPVQSNDGF---------RKVINLYFLGA
ZmNRPD2/E2c 307 WVTDHPSWDAS--YLSQI--------RREKINIKLVPSKSNE-------SCKVINICFMGT

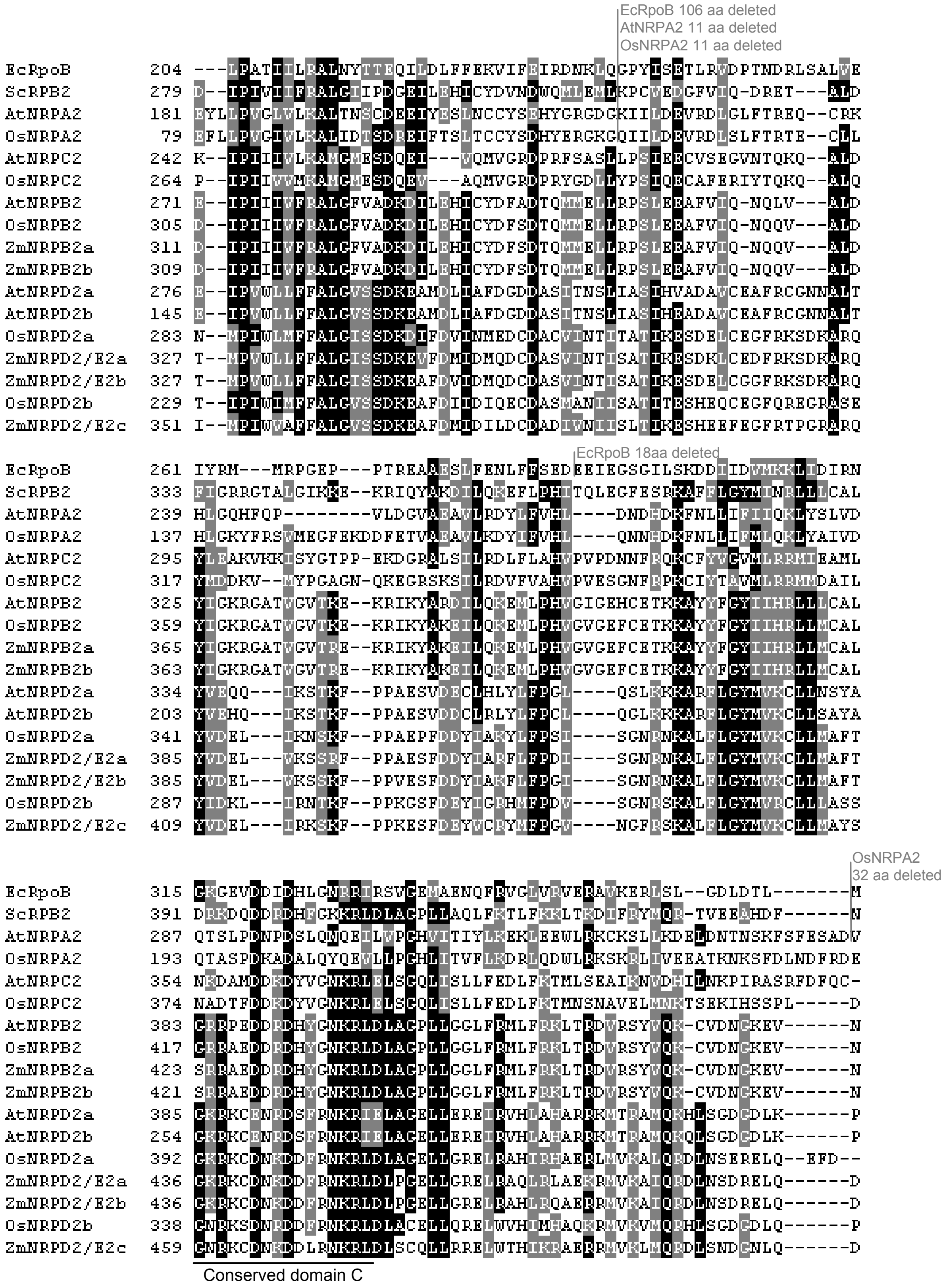

EcRpoB 106 aa deleted
AtNRPA2 11 aa deleted
OsNRPA2 11 aa deleted
EcRpoB 18aa deleted
OsNRPA2
32 aa deleted
Conserved domain C

```
EcRpoB       366 PQDINAK---PISAAVKEFFGSS--------------QLSQFMDQNNPLSEITHKRRISA
ScRPB2       444 MKLINAK---TITSGLKYALATGNWGEQ-KKAMSSRAGVSQVLNRYTYSSTLSHLRRTNT
AtNRPA2      347 KKLINKNPPRSIGTSIETLLKTCALKTQSGLDLQQRAGYTVQAERLNFLRFLSFFRAVHR
OsNRPA2      253 VRKLSKTSA-YVGKAIQSMIKVGKVNSQSGLDLPQRDGMTIHAERLNFHRYISHFRSVHR
AtNRPC2      413 LNKDSRY---SISLGLERTLSTGNFDIK--RFRMHRKGMTQVLTRLSFIGSMGFITKISP
OsNRPC2      429 LLHIKEN---IITHGLERAISTGNWDIK--RFRMHRKGVSQVLSRLSYMASLGYMTRITP
AtNRPB2      436 LQFIKAK---TITSGLKYSLATGNWGQA--NAAGTRAGVSQVLNRLTYASTLSHLRRLNS
OsNRPB2      470 LQFIKAK---TITSGLKYSLATGNWGQA--NQAGTRAGVSQVLNRLTYASTLSHLRRLNS
ZmNRPB2a     476 LQFIKAK---TITSGLKYSLATGNWGQA--NQAGTRAGVSQVLNRLTYASTLSHLRRLNS
ZmNRPB2b     474 LQFIKAK---TVTSGLKYSLATGNWGQA--NQAGTRAGVSQ- Gap of unknown length -
AtNRPD2a     439 IEHLDAS---VITNGLSRAFSTGAWSHPF-RKMERVSGVVANLGRANPLQTLIDLRRTRQ
AtNRPD2b     308 IEHLDAS---VITNGLNRAFSTGAWSHPF-RKMERVSGVVANLGRANPLQTLIDLRRTRQ
OsNRPD2a     448 --HLDAS---IITNGLNRAFSTGSWCHPY-KRNERCAGIVATLRRTNPLQMISDLRKTRQ
ZmNRPD2/E2a  490 LERIDAS---IVTNGLNRAFSTGSWCHPY-KRAERCSGIVATLRRTNPLQMMSDLRKTRQ
ZmNRPD2/E2b  490 LERIDAS---IVTNGLNRAFSTGSWCHPY-KRAERCSGIVATLRRTNPLQMMSDLRKTRQ
OsNRPD2b     392 LECVHAS---IVTNGLNRAFSTGSWCHPF-NKRERCSGIVATLRRTNPLQMMSDMRKTRQ
ZmNRPD2/E2c  514 LRRVDAS---IITNGLNRAFSTGSWRHP--YKKERCSGVVATLRRTNPLQMMSDVRKTRQ
                   ZmNRPD2/E2c
                   1 aa deleted

EcRpoB       409 LGPGGLTRERAGFEVRDVHPTHYGRVCPIETPEGPNIGLINSLSVYAQTNEY--------
ScRPB2       500 P----IGRDGKLAKPRQLHNTHWGLVCPAETPEGQACGLVKNLSLMSCISVGTDPMPIIT
AtNRPA2      407 GA---SFAGLRTTTVRKLLPESWGFLCPVHTPDGTPCG-LLNHMTRTSRITSQFDSKGNI
OsNRPA2      312 GS---SFAKMRTTSVRKLLPESWGFLCPVHTPDGEPCGLLNHMTSTCRISS-FYNSEGAT
AtNRPC2      468 QF----EKSRKVSGPRSLQPSQWGMLCPCDTPEGESCGLVKNLALMTHVTTDEEEGPLVA
OsNRPC2      484 Q----FEKTRKTSGPRALQPSQWGMLCPCDTPEGEACGLTKNLALLTHVTTDQEEGPLMN
AtNRPB2      491 P----IGREGKLAKPRQLHNSQWGMMCPAETPEGQACGLVKNLALMVYITVGSAAYPILE
OsNRPB2      525 P----IGREGKLAKPRQLHNSHWGMMCPAETPEGQACGLVKNLALMVYITVGSAANPILE
ZmNRPB2a     531 P----IGREGKLAKPRQLHNSHWGMMCPAETPEGQACGLVKNLALMVYITVGSAANPILE
ZmNRPB2b     510 ---------GKLAKPRQLHNSHWGMMCPAETPEGQACGLVKNLALMVYITVGSAANPILE
AtNRPD2a     495 Q----VLYTGKVGDARYPHPSHWGRVCFLSTPDGENCGLVKNMSLLGLVSTQSLESVVEK
AtNRPD2b     364 Q----VLYTGKVGDARHPHPSHWGRVCFLSTPDGENCGLVKNMSLLGLVSTQGLESVVEM
OsNRPD2a     502 R----VAYACKAGDARYPNPSYWGKLCFMSTPDGENCGLVKNLAVTATVSSRVAPPLIDR
ZmNRPD2/E2a  546 R----VAYACKAGDARYPNPSYWGKLCFMSTPDGENCGLVKNLAVTAIVSSRVMQPLIES
ZmNRPD2/E2b  546 R----VAYACKAGDARYPNPSYWGKLCFMSTPDGENCGLVKNLAVTSIVSSKVVQPLIES
OsNRPD2b     448 W----VAYACKAGDARYPNPSYWGKLCFLSTPDGEKCGFVKNLAITAIVSCLAREPSVDA
ZmNRPD2/E2c  570 W----FAYACTAGDARYPNPSYWGKLCFLSTPDAEKCGFVKNLAVTAVVSSVVRKPLIDT
                                       Conserved domain D
                                 AtNRPA2 11 aa deleted
                                 OsNRPA2 11 aa deleted

EcRpoB       461 ------------------------------------------------------------
ScRPB2       556 FLSEWGMEPLEDY--VPHQSPDATRVFVNGVWHGVH--RNPARLMETLRTLRRKGDI---
AtNRPA2      463 RDFLKIRKSVVDVKLVRAGPPKVIHVLLDGQVVGTLSSNLVTKVVSYIRRLRVEAPSVIP
OsNRPA2      368 KDFQKIKMSLIARRIERTGPPEVLHVHWDGCIVCS------------IASAKIEEVI--P
AtNRPC2      524 MCYKLGVTDLEVLSAEELHTPDSFLVILNGLILGKH--SRPQYFANSLRRLRRAGKI---
OsNRPC2      540 LCYSLGVEDLSLLSGEEIHASGSFLVMFNGLILGKH--RQPQRFANAMRKLRRSGII---
AtNRPB2      547 FLEEWGTENFEEI--SPSVIPQATKIFVNGMWVGVH--RDPDMLVKTLRRLRRRVDV---
OsNRPB2      581 FLEEWGTENFEEI--SPAVIPQAAKIFVNGCWVGIH--RNPDLLVKTLRRLRRQIDV---
ZmNRPB2a     587 FLEEWGTENFEEI--SPAVIPQAAKIFVNGCWVGIH--RNPDLLVKTLRRLRRQIDV---
ZmNRPB2b     561 FLEEWGTENFEEI--SPAVIPQAAKIFVNGCWVGIH--RNPDLLVKTLRRLRRQIDV---
AtNRPD2a     551 LFACGMEELMDD---TCTPLFGKHKVLLNGDWVGLC--ADSESFVAELKSRRRQSEL---
AtNRPD2b     420 LFTCGMEELMND---TSTPLCGKHKVLLNGDWVGLC--ADSESFVGELKSRRRQS-----
OsNRPD2a     558 FISCGMNKLHEI---PTEEVPRMDKIFLNGDWVGSC--SDPASFVLRLRCMRRSGLI---
ZmNRPD2/E2a  602 FISCGMSKLNDI---PTEHIQRMDKIFLNGNWVGSC--ENSASFVFRLRCMRRSSLI---
ZmNRPD2/E2b  602 FISCGMNKLNDI---PTEHIQRMDKIFLNGNWLGSC--SDSASFVFRLRCMRRSSLI---
OsNRPD2b     504 LVSCGMKKLDEL---LLQEISGKDRIFLNGNLVGVC--ADSVEFVLHLRSMRRRKQI---
ZmNRPD2/E2c  626 FVSCGMKKLDDI---SLQDISGKDRIFLNGSLLGVC--ADPHELTLRLRSLRRSKLI---
```

```
                                                ScRPB2 6 aa deleted
EcRpoB        461 --------------------------GFLETPYRKV------------------------
ScRPB2        609 ----NPEWSMI-RDIREKELKIFTDAGRWYRPLFIVEDKELKVRK-GHIAKLMATEYQDI
AtNRPA2       523 EDLEVGYWPTS-MGGSYPGLYLASCPARFIRPV---------------------------
OsNRPA2       414 EDLEVGYWPLS-HGGAYPGLYLFTNPARFLRPAF--------------------------
AtNRPC2       579 ----GEFWSWF-TNEKQHCVYWASDVGRWCRPLVIADKGISRVKQ-HHMKELQ----DGV
OsNRPC2       595 ----GEFWSIF-VNEKQHCIHIASDGGRWCRPLIIADKGIPRVKE-HHMKQLR----DGI
AtNRPB2       600 ----NTEWGWV-RDIRLKELRIYTDYGRCSRPLFIVDNQKLLIKK-RDIYALQQRESAEE
OsNRPB2       634 ----NTEWGWV-RDIRLKELRLYTDYGRCSRPLFIVENQRLLIKK-RHIRALQQRETPEE
ZmNRPB2a      640 ----NTEWGWV-RDIRLKELRLYTDYGRCSRPLFIVEGQRLLIKK-AHIRALQQRETPDE
ZmNRPB2b      614 ----NTEWGWV-RDIRLKELRLYTDYGRCSRPLFIVEGQRLLIKK-AHIRALQQRETPDE
AtNRPD2a      603 ----PREMEIK-RDKDDNEVRIFTDAGRLLRPLLVVEN------L-QKLKQEK----PSQ
AtNRPD2b      470 ------ELPLE-RDKDDNEVRIFTDAGRLLRPLLVVEN------L-HKLKQDK----PTQ
OsNRPD2a      610 ----DPQWEIK-RDKHQREVRVFSDAGRILRPLLVVEN----------LNKIR----RPK
ZmNRPD2/E2a   654 ----DPQWEIK-RDKHHNEVRVFTDAGRILRPLLVVEN----------LNKIR----KPK
ZmNRPD2/E2b   654 ----DPQWEIK-RDKHYKEVRLFSDAGRILRPLLVVEN----------LNKIR----KPK
OsNRPD2b      556 ----DAQWEIK-RDKQNKEVRIFSDPGRILRPLLIVEN-------LRNIMNRK----NGS
ZmNRPD2/E2c   678 ----DPQWEIK-RDKHHKEVRVFSDPGRIMRPLLVVEN------L-RRITRPK----DGL

                         ScRPB2 4 aa deleted
EcRpoB        471 ------------TDGVVTDEIHYLSAIEEGNYVIAQANSNLDEEGHFVEDLVTCRSKGES
ScRPB2        663 EGGFEDVWSSLLNEGLVEYIDAEEEESILIAMQPEDLEPAEANEENDLDVDPAKRIRVSH
AtNRPA2       555 -------KNISIPSDNIELIGPFEQVANPINIIFISTFP---------------------
OsNRPA2       447 ---------------------------MEIRCPDGGDGGRNKLFP---------------
AtNRPC2       629 RT-----FDDFIRDGLIEYLDVNEENNALWCLRAEAAKAD--------------------
OsNRPC2       645 RS-----FDDFLRDGLIEYLDVNEENNALIALYEHEDQDDVQRSS---------------
AtNRPB2       654 DG-----WHHLVAKGFIEYIDTEEEETTMISMTISDLVQARLRPEEAYTEN---------
OsNRPB2       688 G------WHDLVAKGFIEYIDTEEEETTMISMTINDLIGARHNPEEAYSET----------
ZmNRPB2a      694 G------WHELVSKGYIEYIDTEEEETTMISMTINDLQNARHNPEEAYSET----------
ZmNRPB2b      668 G------WHELVSKGYIEYIDTEEEETTMISMTINDLQNARHNPEEAYSET----------
AtNRPD2a      647 YP-----FDHLLDHGILELIGIEEEEDCNTAWGIKQLLKEPKI-----------------
AtNRPD2b      512 YP-----FKHLLDQGILELIGIEEEEDCTTAWGIKQLLKEPKN-----------------
OsNRPD2a      651 GSSYSFQWLMQQEIIEFIGVEEEEDIRSAWGIRNLFESEEEAPMVKMNKAEDVFNVKRKI
ZmNRPD2/E2a   695 GRSFS--FHELMQQEIIEFIGVEEEEDIQCAWGIRHLFESEGAISS--------------
ZmNRPD2/E2b   695 GRSFS--FQELMQQEIIEFIGVEEEEDIQCAWGIRHLFESEGAISS--------------
OsNRPD2b      600 YS-----FQELMDQNIIELIGVEEEEDIRCAYGIRHLFAGDEEKNFSF------------
ZmNRPD2/E2c   722 YS-----FQELIDQNIVELIGVEEEEDIQCAYGIRHLFSSREKEDWSSSG----------

EcRpoB        519 SLFSRDQVDYMDVSTQQVVSVGASLIPFLEHDDANRALMGANMQR-QAVPT------LRA
ScRPB2        723 HATT---FTHCEIHPSMILGVAASIIPFPDHNQSPRNTYQSAMGK-QAMGVFLTNYNVRM
AtNRPA2       587 -------ATHEEIHPTGMISVVANLTPWSDHNQSPRNMYQCQMAK-QTMAYSTQALQFRA
OsNRPA2       465 -------ATHEEIHPTAILSVVANLTPWSDHNQSPRNMYQCQMAK-QTMGFCGQALKFRT
AtNRPC2       664 -------TTHIEIEPFTILGVVAGLIPYPHHNQSPRNTYQCAMGK-QAMGNIAYNQLNRM
OsNRPC2       685 -------ITHIEIEPLTILGVVAGLIPYPHHNQSPRNTYQCAMGK-QAMGNIAYNQLFRA
AtNRPB2       700 -------YTHCEIHPSLILGVCASIIPFPDHNQSPRNTYQSAMGK-QAMGIYVTNYQFRM
OsNRPB2       733 -------YTHCEIHPSLILGVCASIIPFPDHNQSPRNTYQSAMGK-QAMGIYVTNYQLRM
ZmNRPB2a      739 -------YTHCEIHPSLILGVCASIIPFPDHNQSPRNTYQSAMGK-QAMGIYVTNYQLRM
ZmNRPB2b      713 -------YTHCEIHPSLILGVCASIIPFPDHNQSPRNTYQSAMGK-QAMGIYVTNYQLRM
AtNRPD2a      685 -------YTHCELDLSFLLGVSCAVVPFANHDHGRRVLYQSQKHCQQAIGFSSTNPNIRC
AtNRPD2b      550 -------YTHCELDLSFLLGVSCAIVPFANHDHGKRVLYQSQKHCQQAIGFSSTNPNIRC
OsNRPD2a      711 GGEVSG-YTHCELDLSFLLGLSCGIIPFANHNFARRVLYQSEKHSQQAIGYSTTNPHIRV
ZmNRPD2/E2a   739 -------YTHCELDPSFLLGLSCGIIPFANHNFARRVLYQSEKHSQQAIGYSTTNPHIRV
ZmNRPD2/E2b   739 -------YTHCELDPSFILGLSCGIIPFANHNFARRVLYQSEKHSQQAIGYSTSNPRIRV
OsNRPD2b      643 -------YTHCELDPSFLLGLSCSIIPFANHDTAKRILMQAEKISQQAIGYSTTNPLFRV
ZmNRPD2/E2c   767 -------YTHCELDPSFLLGLSCSLIPFGNHDNARRVQMQAEKISQQAIGYSPTNSQYRL
                                ___________________
                                Conserved domain E
```

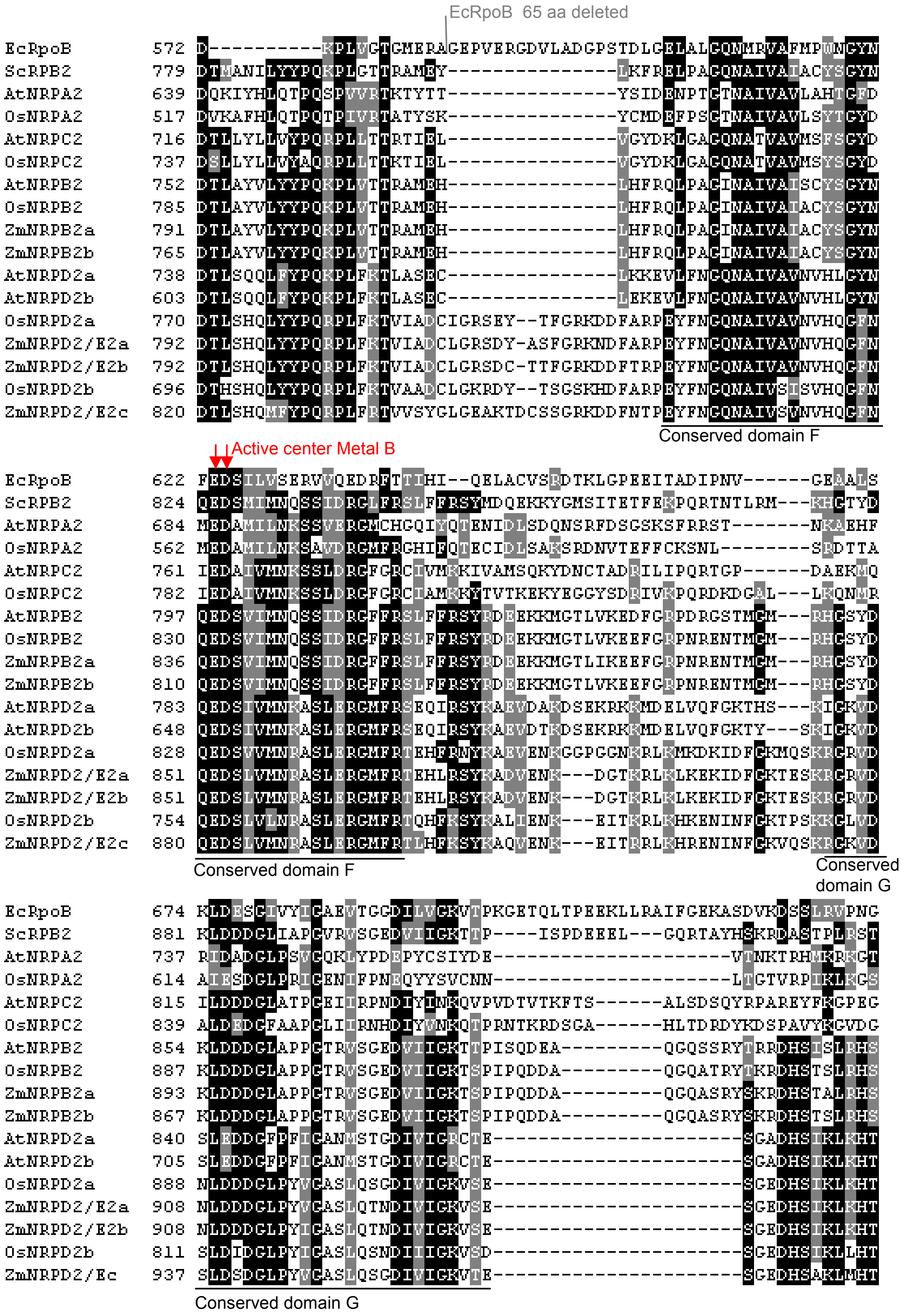
EcRpoB 65 aa deleted
EcRpoB 572 D----------KPLVGTGMERAGEPVERGDVLADGPSTDLGELALGQNMRVAFMPWNGYN
ScRPB2 779 DTMANILYYPQKPLGTTRAMEY---------------LKFRELPAGQNAIVAIACYSGYN
AtNRPA2 639 DQKIYHLQTPQSPVVRTKTYTT---------------YSIDENPTCTNAIVAVLAHTGFD
OsNRPA2 517 DVKAFHLQTPQTPIVRTATYSK---------------YCMDEFPSCTNAIVAVLSYTGYD
AtNRPC2 716 DTLLYLLVYPQRPLLTTRTIEL---------------VGYDKLGAGQNATVAVMSFSGYD
OsNRPC2 737 DSLLYLLVYAQRPLLTTKTIEL---------------VGYDKLGAGQNATVAVMSYSGYD
AtNRPB2 752 DTLAYVLYYPQKPLVTTRAMEH---------------LHFRQLPACINAIVAISCYSGYN
OsNRPB2 785 DTLAYVLYYPQKPLVTTRAMEH---------------LHFRQLPACINAIVAIACYSGYN
ZmNRPB2a 791 DTLAYVLYYPQKPLVTTRAMEH---------------LHFRQLPACINAIVAIACYSGYN
ZmNRPB2b 765 DTLAYVLYYPQKPLVTTRAMEH---------------LHFRQLPACINAIVAIACYSGYN
AtNRPD2a 738 DTLSQQLFYPQKPLFKTLASEC---------------LKKEVLFNGQNAIVAVNVHLGYN
AtNRPD2b 603 DTLSQQLFYPQKPLFKTLASEC---------------LEKEVLFNGQNAIVAVNVHLGYN
OsNRPD2a 770 DTLSHQLYYPQRPLFKTVIADCIGRSEY--TFGRKDDFARPEYFNGQNAIVAVNVHQGFN
ZmNRPD2/E2a 792 DTLSHQLYYPQRPLFKTVIADCLGRSDY-ASFGRKNDFARPEYFNGQNAIVAVNVHQGFN
ZmNRPD2/E2b 792 DTLSHQLYYPQRPLFKTVIADCLGRSDC-TTFGRKDDFTRPEYFNGQNAIVAVNVHQGFN
OsNRPD2b 696 DTHSHQLYYPQRPLFKTVAADCLGKRDY--TSGSKHDFARPEYFNGQNAIVSISVHQGFN
ZmNRPD2/E2c 820 DTLSHQMFYPQRPLFRTVVSYGLGEAKTDCSSGRKDDFNTPEYFNGQNAIVSVNVHQGFN
Conserved domain F
Active center Metal B
EcRpoB 622 FEDSILVSERVVQEDRFTTIHI--QELACVSRDTKLGPEEITADIPNV------GEAALS
ScRPB2 824 QEDSMIMNQSSIDRGLFRSLFFRSYMDQEKKYGMSITETFEKPQRTNTLRM---KHGTYD
AtNRPA2 684 MEDAMILNKSSVERGMCHGQIYQTENIDLSDQNSRFDSGSKSFRRST-------NKAEHF
OsNRPA2 562 MEDAMILNKSAVDRGMFRGHIFQTECIDLSAKSRDNVTEFFCKSNL--------SRDTTA
AtNRPC2 761 IEDAIVMNKSSLDRGFGRCIVMKKIVAMSQKYDNCTADRILIPQRTGP------DAEKMQ
OsNRPC2 782 IEDAIVMNKSSLDRGFGRCIAMKKYTVTKEKYEGGYSDRIVKPQRDKDGAL---LRQNMR
AtNRPB2 797 QEDSVIMNQSSIDRGFFRSLFFRSYRDEEKKMGTLVKEDFGRPDRGSTMGM---RHGSYD
OsNRPB2 830 QEDSVIMNQSSIDRGFFRSLFFRSYRDEEKKMGTLVKEEFGRPNRENTMGM---RHGSYD
ZmNRPB2a 836 QEDSVIMNQSSIDRGFFRSLFFRSYRDEEKKMGTLIKEEFGRPNRENTMGM---RHGSYD
ZmNRPB2b 810 QEDSVIMNQSSIDRGFFRSLFFRSYRDEEKKMGTLVKEEFGRPNRENTMGM---RHGSYD
AtNRPD2a 783 QEDSIVMNKASLERGMFRSEQIRSYKAEVDAKDSEKRKKMDELVQFGKTHS---KIGKWD
AtNRPD2b 648 QEDSIVMNKASLERGMFRSEQIRSYKAEVDTKDSEKRKKMDELVQFGKTY---SKIGKWD
OsNRPD2a 828 QEDSVVMNRASLERGMFRTEHFRNYKAEVENKGGPGGNKRLKMKDKIDFGKMQSKRGRWD
ZmNRPD2/E2a 851 QEDSLVMNRASLERGMFRTEHLRSYKADVENK---DGTKRLKLKEKIDFGKTESKRGRWD
ZmNRPD2/E2b 851 QEDSLVMNRASLERGMFRTEHLRSYKADVENK---DGTKRLKLKEKIDFGKTESKRGRWD
OsNRPD2b 754 QEDSLVLNRASLERGMFRTQHFKSYKALIENK---EITKRLKHKENINFGKTPSKKGLWD
ZmNRPD2/E2c 880 QEDSLVMNRASLERGMFRTLHFKSYKAQVENK---EITRRLKHRENINFGKVQSKRGKWD
Conserved domain F
Conserved domain G
EcRpoB 674 KLDESGIVYIGAEVTGGDILVGKVTPKGETQLTPEEKLLRAIFGEKASDVKDSSLRVPNG
ScRPB2 881 KLDDDGLIAPGVRVSGEDVIIGKTTTP----ISPDEEL---GQRTAYHSKRDASTPLRST
AtNRPA2 737 RIDADGLPSVGQKLYPDEPYCSIYDE---------------------VTNKTRHMKRKGT
OsNRPA2 614 AIESDGLPRIGENIFPNEQYYSVCNN---------------------LTGTVRPIKLKGS
AtNRPC2 815 ILDDDGLATPGEIIRPNDIYINKQVPVDTVTKFTS------ALSDSQYRPAREYFKGPEG
OsNRPC2 839 ALDEDGFAAPGLIIRNHDIYVNKQTPRNTKRDSGA------HLTDRDYKDSPAVYKGVDG
AtNRPB2 854 KLDDDGLAPPGTRVSGEDVIIGKTTPISQDEA---------QGQSSRYTRRDHSISLRHS
OsNRPB2 887 KLDDDGLAPPGTRVSGEDVIIGKTSPIPQDDA---------QGQATRYTKRDHSTSLRHS
ZmNRPB2a 893 KLDDDGLAPPGTRVSGEDVIIGKTSPIPQDDA---------QGQASRYSKRDHSTALRHS
ZmNRPB2b 867 KLDDDGLAPPGTRVSGEDVIIGKTSPIPQDDA---------QGQASRYSKRDHSTSLRHS
AtNRPD2a 840 SLEDDGFPFIGANMSTGDIVIGRCTE----------------------SGADHSIKLKHT
AtNRPD2b 705 SLEDDGFPFIGANMSTGDIVIGRCTE----------------------SGADHSIKLKHT
OsNRPD2a 888 NLDDDGLPYVGASLQSGDIVIGKVSE----------------------SGEDHSIKLKHT
ZmNRPD2/E2a 908 NLDDDGLPYVGASLQTNDIVIGKVSE----------------------SGEDHSIKLKHT
ZmNRPD2/E2b 908 NLDDDGLPYIGASLQTNDIVIGKVSE----------------------SGEDHSIKLKHT
OsNRPD2b 811 SLDIDGLPYIGASLQSNDIIIGKVSD----------------------SGEDHSIKLLHT
ZmNRPD2/Ec 937 SLDSDGLPYVGASLQSGDIVIGKVTE----------------------SGEDHSAKLMHT
Conserved domain G

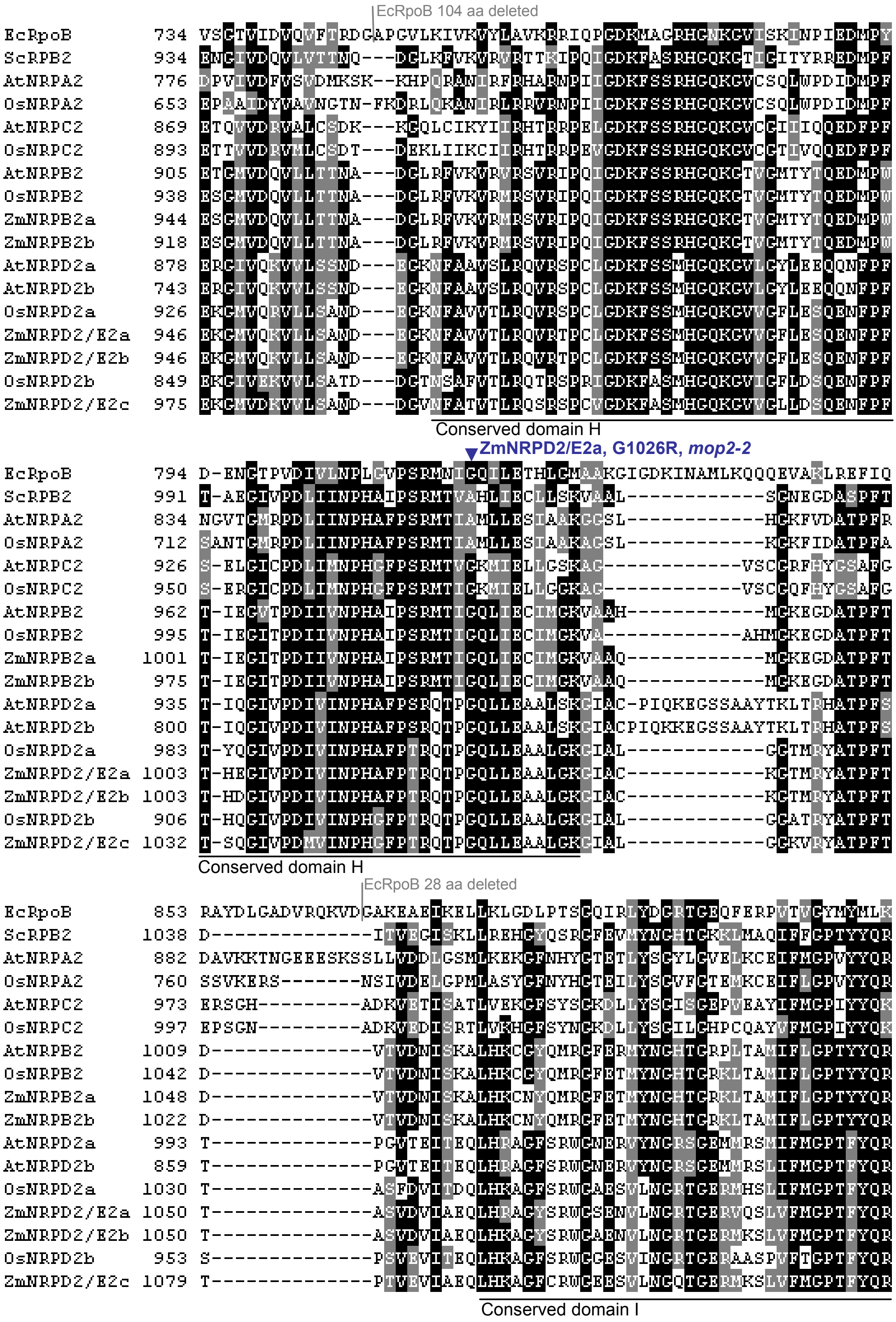
EcRpoB 104 aa deleted
EcRpoB 734 VSGTVIDVQVFTRDGAPGVLKIVKVYLAVKRRIQPGDKMAGRHGNKGVISKINPIEDMPY
ScRPB2 934 ENGIVDQVLVTTNQ---DGLKFVKVRVRTTKIPQIGDKFASRHGQKGTIGITYRREDMPF
AtNRPA2 776 DPVIVDFVSVDMKSK--KHPQRANIRFRHARNPIIGDKFSSRHGQKGVCSQLWPDIDMPF
OsNRPA2 653 EPAAIDYVAVNGTN-FKDRLQKANIRLRRVRNPIIGDKFSSRHGQKGVCSQLWPDIDMPF
AtNRPC2 869 ETQVVDRVALCSDK---KGQLCIKYIIRHTRRPELGDKFSSRHGQKGVCGIIIQQEDFPF
OsNRPC2 893 ETTVVDRVMLCSDT---DEKLIIKCIIRHTRRPEVGDKFSSRHGQKGVCGTIVQQEDFPF
AtNRPB2 905 ETGMVDQVLLTTNA---DGLRFVKVRVRSVRIPQIGDKFSSRHGQKGTVGMTYTQEDMPW
OsNRPB2 938 ESGMVDQVLLTTNA---DGLRFVKVRMRSVRIPQIGDKFSSRHGQKGTVGMTYTQEDMPW
ZmNRPB2a 944 ESGMVDQVLLTTNA---DGLRFVKVRMRSVRIPQIGDKFSSRHGQKGTVGMTYTQEDMPW
ZmNRPB2b 918 ESGMVDQVLLTTNA---DGLRFVKVRMRSVRIPQIGDKFSSRHGQKGTVGMTYTQEDMPW
AtNRPD2a 878 ERGIVQKVVLSSND---EGKNFAAVSLRQVRSPCLGDKFSSMHGQKGVLGYLEEQQNFPF
AtNRPD2b 743 ERGIVQKVVLSSND---EGKNFAAVSLRQVRSPCLGDKFSSMHGQKGVLGYLEEQQNFPF
OsNRPD2a 926 EKGMVQRVLLSAND---EGKNFAVVTLRQVRSPCLGDKFSSMHGQKGVVGFLESQENFPF
ZmNRPD2/E2a 946 EKGMVQKVLLSAND---EGKNFAVVTLRQVRTPCLGDKFSSMHGQKGVVGFLESQENFPF
ZmNRPD2/E2b 946 EKGMVQKVLLSAND---EGKNFAVVTLRQVRTPCLGDKFSSMHGQKGVVGFLESQENFPF
OsNRPD2b 849 EKGIVEKVVLSATD---DGTNSAFVTLRQTRSPRIGDKFASMHGQKGVIGFLDSQENFPF
ZmNRPD2/E2c 975 EKGMVDKVVLSAND---DGVNFATVTLRQSRSPCVGDKFASMHGQKGVVGLLDSQENFPF
Conserved domain H
ZmNRPD2/E2a, G1026R, *mop2-2*
EcRpoB 794 D-ENGTPVDIVLNPLGVPSRMNIGQILETHLGMAAKGIGDKINAMLKQQQEVAKLREFIQ
ScRPB2 991 T-AEGIVPDLIINPHAIPSRMTVAHLIECLLSKVAAL-------------SGNEGDASPFT
AtNRPA2 834 NGVTGMRPDLIINPHAFPSRMTIAMLLESIAAKGGSL-------------HGKFVDATPFR
OsNRPA2 712 SANTGMRPDLIINPHAFPSRMTIAMLLESIAAKAGSL-------------KGKFIDATPFA
AtNRPC2 926 S-ELGICPDLIMNPHGFPSRMTVGKMIELLGSKAG-------------VSCGRFHYGSAFG
OsNRPC2 950 S-ERGICPDLIMNPHGFPSRMTIGKMIELLGGKAG-------------VSCGQFHYGSAFG
AtNRPB2 962 T-IEGVTPDIIVNPHAIPSRMTIGQLIECIMGKVAAH-------------MGKEGDATPFT
OsNRPB2 995 T-IEGITPDIIVNPHAIPSRMTIGQLIECIMGKVA-------------AHMGKEGDATPFT
ZmNRPB2a 1001 T-IEGITPDIIVNPHAIPSRMTIGQLIECIMGKVAAQ-------------MGKEGDATPFT
ZmNRPB2b 975 T-IEGITPDIIVNPHAIPSRMTIGQLIECIMGKVAAQ-------------MGKEGDATPFT
AtNRPD2a 935 T-IQGIVPDIVINPHAFPSRQTPGQLLEAALSKGIAC-PIQKEGSSAAYTKLTRHATPFS
AtNRPD2b 800 T-IQGIVPDIVINPHAFPSRQTPGQLLEAALSKGIACPIQKKEGSSAAYTKLTRHATPFS
OsNRPD2a 983 T-YQGIVPDIVINPHAFPTRQTPGQLLEAALGKGIAL-------------GGTMRYATPFT
ZmNRPD2/E2a 1003 T-HEGIVPDIVINPHAFPTRQTPGQLLEAALGKGIAC-------------KGTMRYATPFT
ZmNRPD2/E2b 1003 T-HDGIVPDIVINPHAFPTRQTPGQLLEAALGKGIAC-------------KGTMRYATPFT
OsNRPD2b 906 T-HQGIVPDIVINPHGFPTRQTPGQLLEAALGKGIAL-------------GGATRYATPFT
ZmNRPD2/E2c 1032 T-SQGIVPDMVINPHGFPTRQTPGQLLEAALGKGIAL-------------GGKVRYATPFT
Conserved domain H
EcRpoB 28 aa deleted
EcRpoB 853 RAYDLGADVRQKVDGAKEAEIKELLKLGDLPTSGQIRLYDGRTGEQFERPVTVGYMYMLK
ScRPB2 1038 D--------------ITVEGISKLLREHGYQSRGFEVMYNGHTGKKLMAQIFFGPTYYQR
AtNRPA2 882 DAVKKTNGEEESKSSLLVDDLGSMLKEKGFNHYGTETLYSGYLGVELKCEIFMGPVYYQR
OsNRPA2 760 SSVKKERS-------NSIVDELGPMLASYGFNYHGTEILYSGVFGTEMKCEIFLGPVYYQR
AtNRPC2 973 ERSGH----------ADKVETISATLVEKGFSYSGKDLLYSGISGEPVEAYIFMGPIYYQK
OsNRPC2 997 EPSGN----------ADKVEDISRTLVKHGFSYNGKDLLYSGILGHPCQAYVFMGPIYYQK
AtNRPB2 1009 D---------------VTVDNISKALHKCGYQMRGFERMYNGHTGRPLTAMIFLGPTYYQR
OsNRPB2 1042 D---------------VTVDNISKALHKCGYQMRGFETMYNGHTGRKLTAMIFLGPTYYQR
ZmNRPB2a 1048 D---------------VTVDNISKALHKCNYQMRGFETMYNGHTGRKLTAMIFLGPTYYQR
ZmNRPB2b 1022 D---------------VTVDNISKALHKCNYQMRGFETMYNGHTGRKLTAMIFLGPTYYQR
AtNRPD2a 993 T---------------PGVTEITEQLHRAGFSRWGNERVYNGRSGEMMRSMIFMGPTFYQR
AtNRPD2b 859 T---------------PGVTEITEQLHRAGFSRWGNERVYNGRSGEMMRSLIFMGPTFYQR
OsNRPD2a 1030 T---------------ASFDVITDQLHKAGFSRWGAESVLNGRTGERMHSLIFMGPTFYQR
ZmNRPD2/E2a 1050 T---------------ASVDVIAEQLHRAGYSRWGSENVLNGRTGERVQSLVFMGPTFYQR
ZmNRPD2/E2b 1050 T---------------ASVDVIAEQLHKAGYSRWGAENVLNGRTGERMKSLVFMGPTFYQR
OsNRPD2b 953 S---------------PSVEVITEQLHKAGFSRWGGESVINGRTGERAASPVFTGPTFYQR
ZmNRPD2/E2c 1079 T---------------PTVEVIAEQLHKAGFCRWGEESVLNGQTGERMKSLVFMGPTFYQR
Conserved domain I

ZmNRPD2/E2a,E1079K, *Mop2-1*

```
EcRpoB       913  LNHLVDDKMHARSTGSYSLVTQQPLGGKAQFGGQRFGEMEVWALEAYGAAYTLQEMLTVK
ScRPB2      1084  LRHMVDDKIHARARGPMQVLTRQPVEGRSRDGGLRFGEMERDCMIAHGAASFLKERLMEA
AtNRPA2      942  LRHMVSDKFQVRSTGQVDQLTHQPIKGRKRGGGIRFGEMERDSLLAHGASYLLHDRLHTS
OsNRPA2      813  LRHMVSDKFQVRTTGRIDQITRQPIGGRKYGGGIRFGEMERDALLAHGASYLLHDRLHSC
AtNRPC2     1024  LKHMVLDKMHARGSGPRVMMTRQPTEGKSKNGGLRVGEMERDCLIAYGASMLIYERLMIS
OsNRPC2     1048  LKHMVLDKMHARASGPRVLLTRQPTEGRSRDGGLRLGEMERDCLIAYGASMLIFERLLIS
AtNRPB2     1055  LKHMVDDKIHSRGRGPVQILTRQPAEGRSRDGGLRFGEMERDCMIAHGAAHFLKERLFDQ
OsNRPB2     1088  LKHMVDDKIHSRGRGPVQILTRQPAEGRSRDGGLRFGEMERDCMIAHGAAFFLKERLFDQ
ZmNRPB2a    1094  LKHMVDDKIHSRGRGPVQILTRQPAEGRSRDGGLRFGEMERDCMIAHGAAFFLKERLFDQ
ZmNRPB2b    1068  LKHMVDDKIHSRGRGPVQILTRQPAEGRSRDGGLRFGEMERDCMIAHGAAFFLKERLFDQ
AtNRPD2a    1039  LVHMSEDKVKFRNTGPVHPLTRQPVADRKRFGGIKFGEMERDCLIAHGASANLHERLFTL
AtNRPD2b     905  LVHMSENKVKFRNTGPVHPLTRQPVADRKRFGGIRFGEMERDCLIAHGASANLHERLFTL
OsNRPD2a    1076  LIHMAEDKVKFRNTGPVHPLTRQPVADRKRFGGVKFGEMERDCLLAHGAAANLHERLFML
ZmNRPD2/E2a 1096  LIHMSEDKVKFRNTGPVHPLTRQPVADRKRFGGVKFGEMERDCLLAHGSAANLHERLFLL
ZmNRPD2/E2b 1096  LIHMSEDKVKYRNTGPVHPLTRQPVADRKRFGGVKFGEMERDCLLAHGSAANLHERLFML
OsNRPD2b     999  LHHMAEDKVKFRNTGPVHPLTRQPVEDRRRYGGVKFGEMERDCLLAHGAAANLHERLFLL
ZmNRPD2/E2c 1125  LVHMAEDKVKFRNTGPVHPLTRQPVEDKKRFGGVKFGEMERDCMLAHGAAANLHERLFTL
```

Conserved domain I

AtNRPA2 8 aa deleted
OsNRPA2 7 aa deleted

```
EcRpoB       973  SDDVNGRTKMYKNIVD----------------------------GNHQMEP-GMPESFNV
ScRPB2      1144  SDAFR-VHICGICGLMTVIA----------KLNHNQFECKGCDN-KIDIYQI-HIPYAAK
AtNRPA2     1002  SDHHI-ADVCSLCGSLLTSSLIQEIGKLPPGRTPKKVTCYSCKT-SKGMETV-AMPYVFR
OsNRPA2      873  SDYHI-ADVCSICGSLLTATAKRDMLGLPTVKPPKNFACQACKT-SKGMETV-AMPYVFR
AtNRPC2     1084  SDPFE-VQVCRACGLLGYYN-----------YKLKKAVCTTCKN-GDNIATM-KLPYACK
OsNRPC2     1108  SDPYQ-VQVCRKCGLLGYYN-----------HKLKASYCSMCKN-GENMAKM-RMPYACK
AtNRPB2     1115  SDAYR-VHVCEVCGLIAIAN-----------LKKNSFECRGCKN-KTDIVQV-YIPYACK
OsNRPB2     1148  SDAYR-VHVCEKCGLIAIAN-----------LKKNSFECRGCKN-KTDIVQV-HIPYACK
ZmNRPB2a    1154  SDAYR-VHVCEKCGLIAIAN-----------LKKNSFECRGCKN-KTDIVQV-HIPYACK
ZmNRPB2b    1128  SDAYR-VHVCEKCGLIAIAN-----------LKKNSFECRGCKN-KTDIVQV-HIPYACK
AtNRPD2a    1099  SDSSQ-MHICRKCKTYANVI----ERTPSSGRKIRGPYCRVCVS-SDHVVRV-YVPYGAK
AtNRPD2b     965  SDSSQ-MHICRKCKTYANVI----ERTPSSGRKIRGPYCRVCAS-SDHVVRV-YVPYGAK
OsNRPD2a    1136  SDFSQ-MHVCQTCERVANVI----MRPVPGGKKIRGPYCGFCRS-SENIVRI-NVPYGAK
ZmNRPD2/E2a 1156  SDFSQ-MHICQTCERVANVV----MRSVPGGKKIRGPYCGFCKS-SENIVRI-NVPYGAK
ZmNRPD2/E2b 1156  SDFSQ-MHICQTCERVANVV----MRSVPGGKKIRGPYCGFCKS-SENIVRI-NVPYGAK
OsNRPD2b    1059  SDVSR-LHVCRRCQRAAVVVSPAVAADGGGGRKVRGPYCRFCRS-AEEVVRV-SVPYGAK
ZmNRPD2/E2c 1185  SDFSR-MHICQACERVANVI--VRAAEGGGGKKVRGPYCLFCRS-AERVVRV-DVPYGSK
```

```
EcRpoB      1004  LLKEIRSLGINIELEDE--------------
ScRPB2      1191  LLFQELMAMNITPRLYTDRSRDF--------
AtNRPA2     1059  YLAAELASMNIKMTLQLSDREGVTD------
OsNRPA2      930  YLASELAAMNIKLELRLSNRTEHPTTTSEES
AtNRPC2     1130  LLFQELQSMNVVPRLKLTEA-----------
OsNRPC2     1154  LLFQELQAMNVVPRLKLTEG-----------
AtNRPB2     1161  LLFQELMSMAIAPRMLTKHLKSAKGRQ----
OsNRPB2     1194  LLFQELMAMAIAPRMLTQDNKTGKDQKKR--
ZmNRPB2a    1200  LLFQELMAMAIAPRMLTHDMKTGKDQKKR--
ZmNRPB2b    1174  LLFQELMAMAIAPRMLTHDMKTGKDQKKR--
AtNRPD2a    1152  LLCQELFSMGITLNFDTKLC-----------
AtNRPD2b    1018  LLCQELFSMGITLNFDTKLC-----------
OsNRPD2a    1189  LLYQELFSMGICLRFETEVC-----------
ZmNRPD2/E2a 1209  LLYQELFSMGICLKFETQVC-----------
ZmNRPD2/E2b 1209  LLYQELFSMGICLKFETEVC-----------
OsNRPD2b    1116  ILYQELFSMGICLKFDTELI-----------
ZmNRPD2/E2c 1240  LLYQELFSMGICLKFETEVR-----------
```

Organisms designations:
Ec (*Escherichia* coli)
Sc (*Saccharomyces cerevisiae*)
At (*Arabidopsis thaliana*)
Os (*Oryza sativa*)
Zm (*Zea mays)*

Supplementary Figure 3. Alignment of Second Largest Subunits of RNA Polymerases. Alignment was performed using MUSCLE, edited using GENEDOC, and shaded using BOXSHADE. Identical amino acids are shaded in black, while similar amino acids are shaded in gray. Conserved domains are underlined and indicated A though I [72]. The active site (metal B) is indicated by asterisks [72]. Positions of *Mop2-1* and *mop2-2* mutations are indicated above the alignment in blue. Positions and number of amino acids removed from alignment are indicated in gray.
