# Supplementary material for: A Dominant Mutation in mediator of paramutation2, One of Three Second-Largest Subunits of a Plant-Specific RNA Polymerase, Disrupts Multiple siRNA Silencing Processes
Source: PLoS Genet. 2009 Nov 20;5(11):e1000725. doi: 10.1371/journal.pgen.1000725 (PMC2774164; doi:10.1371/journal.pgen.1000725)
Supplement: Figure S4 — Crossing schema for experiment to test effect of Mop2-1 on p1 paramutation. The paramutagenic P1-rr' allele has light patterned pericarp, while paramutable P1-rr has red pericarp pigment. The P1.2b::GUS transgene carried the highly paramutagenic P2P147-37 integration event [13]. To assay whether the Mop2-1 mutation would prevent p1 paramutation, plants carrying the paramutagenic endogenous P1-rr' allele and the P1.2b::GUS transgene were pollinated with the Mop2-1 P1-rr stock. Because the B' allele was introduced together with the Mop2-1 mutation we used dark plant pigment for initial identification of Mop2-1 homozygous plants in segregating families, and subsequent molecular markers were used to verify the Mop2-1/Mop2-1 and Mop2-1/+ genotypes. Spraying with the BASTA herbicide eliminated non transgenic plants. In the F1 all transgenic plants had light pericarp color indicating that when Mop2-1 is heterozygous it does not p1 prevent paramutation. A backcross with the Mop2-1 stock was used to generate families in which the effect of homozygous Mop2-1 on preventing p1 paramutation was assayed. Results are presented in Table 2. (0.17 MB PDF) [file pgen.1000725.s004.pdf]

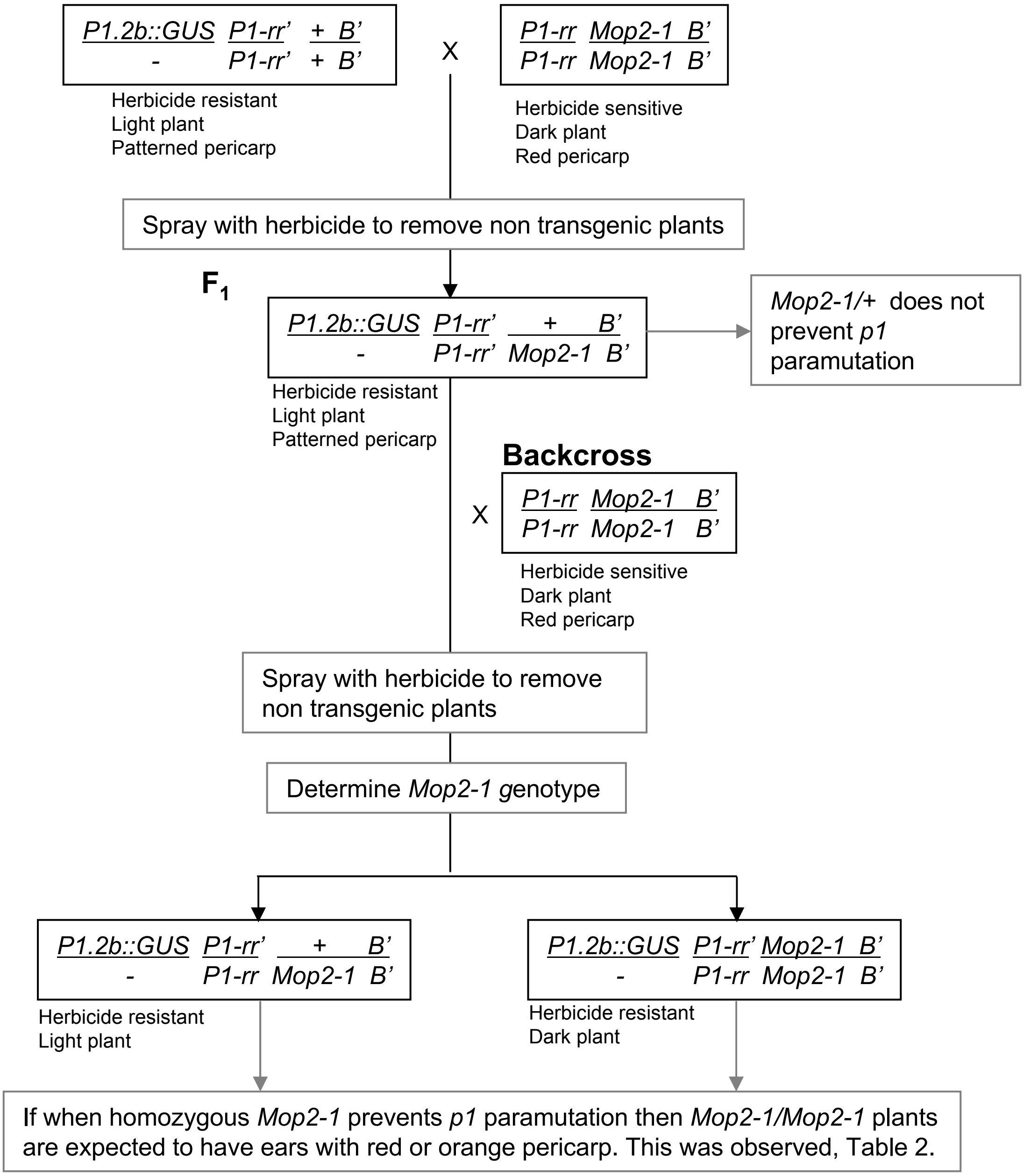

Figure S4. Crossing schema to test whether *Mop2-1* prevents *p1* paramutation. The paramutagenic *P1-rr'* allele has light patterned pericarp, while paramutable *P1-rr* has red pericarp pigment. The P1.2b::GUS transgene carried the highly paramutagenic P2P147-37 integration event [13]. To assay whether the *Mop2-1* mutation would prevent *p1* paramutation, plants carrying the paramutagenic endogenous *P1-rr'* allele and the P1.2b::GUS transgene were pollinated with the *Mop2-1 P1-rr* stock. Because the *B'* allele was introduced together with the *Mop2-1* mutation we used dark plant pigment for initial identification of *Mop2-1* homozygous plants in segregating families, and subsequent molecular markers were used to verify the *Mop2-1/Mop2-1* and *Mop2-1/+* genotypes. Spraying with the BASTA herbicide eliminated non transgenic plants. In the F_1 all transgenic plants had light pericarp color indicating that when *Mop2-1* is heterozygous it does not *p1* prevent paramutation. A backcross with the *Mop2-1* stock was used to generate families in which the effect of homozygous *Mop2-1* on preventing *p1* paramutation was assayed. Results are presented in Table 2.
