# Supplementary material for: A Dominant Mutation in mediator of paramutation2, One of Three Second-Largest Subunits of a Plant-Specific RNA Polymerase, Disrupts Multiple siRNA Silencing Processes
Source: PLoS Genet. 2009 Nov 20;5(11):e1000725. doi: 10.1371/journal.pgen.1000725 (PMC2774164; doi:10.1371/journal.pgen.1000725)
Supplement: Figure S5 — Schematic drawing of genetic experiment that tests Mop2-1 effect on preventing r1 paramutation. Plants heterozygous for Mop2-1/+ and carrying R-st/r or R-r/r were crossed to produce F1 plants. Although r1 paramutation occurs in the F1, observation of paramutation requires a testcross to a colorless allele that does not participate in paramutation (r) to obtain seeds in which pigment levels reveal the extent of paramutation of R-r to R-r' [81]. To produce testcross progeny, mottled and fully colored F1 seeds (R-st/R-r' or r/R-r) were planted. Resulting plants were genotyped for the Mop2-1 mutation and out crossed onto silks carrying the r allele. Seeds resulting from the test cross were sorted to identify mottled and/or full colored seeds and light reflectance was measured to determine the relative color of the seeds. Data summarized in Figure 6C. (0.10 MB PDF) [file pgen.1000725.s005.pdf]

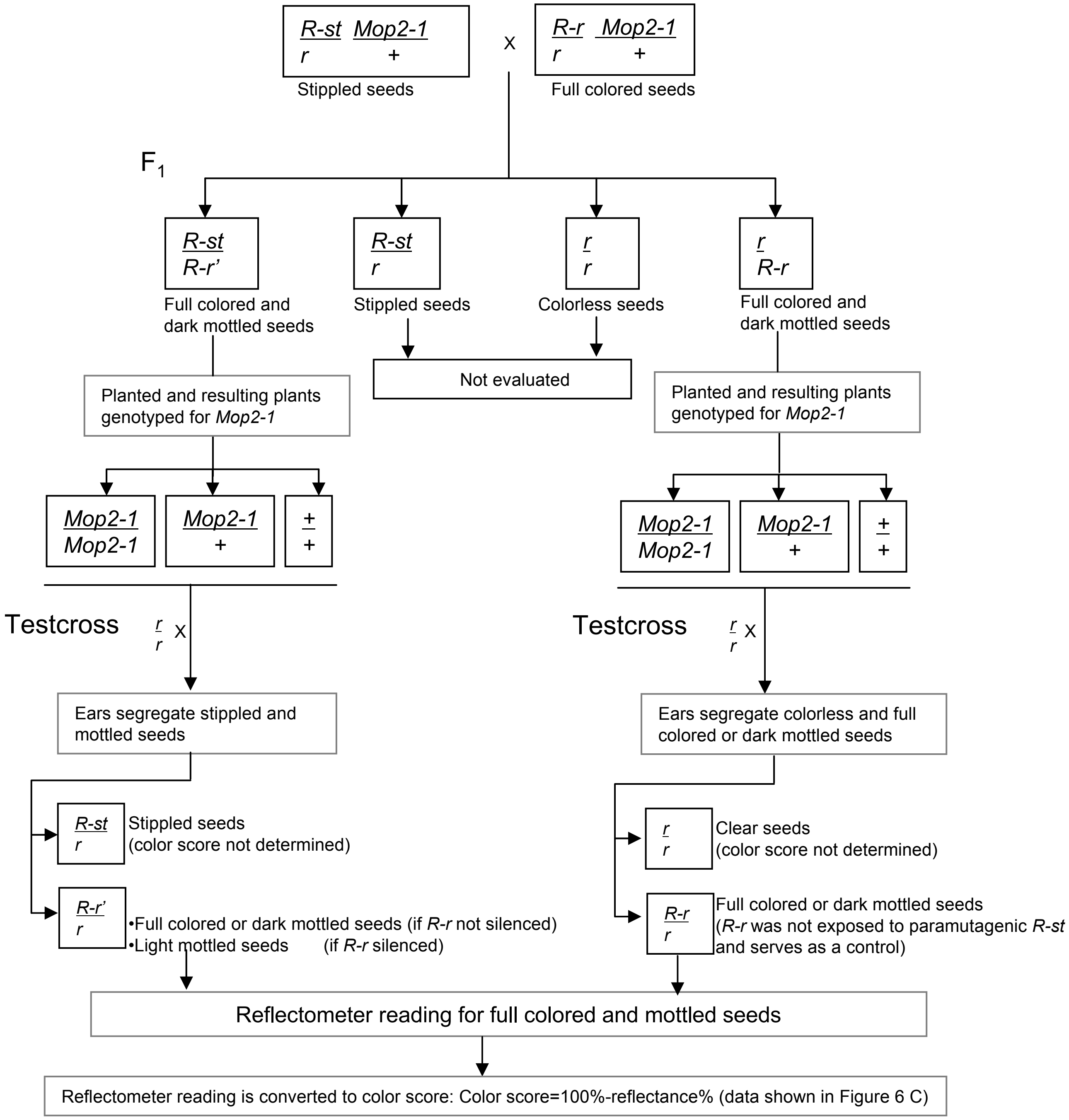

Figure S5. Schematic drawing of genetic experiment to test whether *Mop2-1* prevents *r1* paramutation. Plants heterozygous for *Mop2-1/+* and carrying *R-st/r* or *R-r/r* were crossed to produce F_1 plants. Although *r1* paramutation occurs in the F_1, observation of paramutation requires a testcross to a colorless allele that does not participate in paramutation *(r)* to obtain seeds in which pigment levels reveal the extent of paramutation of *R-r* to *R-r'* [81]. To produce testcross progeny, mottled and fully colored F_1 seeds (*R-st/R-r'* or *r/R-r*) were planted. Resulting plants were genotyped for the *Mop2-1* mutation and out crossed onto silks carrying the *r* allele. Seeds resulting from the test cross were sorted to identify mottled and/or full colored seeds and light reflectance was measured to determine the relative color of the seeds. Data summarized in Figure 6C.
