# Supplementary material for: A Dominant Mutation in mediator of paramutation2, One of Three Second-Largest Subunits of a Plant-Specific RNA Polymerase, Disrupts Multiple siRNA Silencing Processes
Source: PLoS Genet. 2009 Nov 20;5(11):e1000725. doi: 10.1371/journal.pgen.1000725 (PMC2774164; doi:10.1371/journal.pgen.1000725)
Supplement: Figure S6 — Schematic drawing of genetic experiment that tests Mop2-1 effect on Ubi::MS45pIR and Ubi::5126pIR transgene induced silencing. pIR is used to symbolize the inverted repeat transgenes. Dark plant pigmentation specified by B' in Mop2-1/Mop2-1 plants was used to classify progeny. Molecular genotyping of a subset of plants revealed close correspondence between the B' phenotype and the Mop2-1 genotype; 28/33 dark plants were homozygous and 28/30 light plants were heterozygous for Mop2-1. Recombination between the linked b1 and mop2 loci does not influence outcome of this experiment because at least one B' allele is present to report the Mop2-1 genotype. (0.16 MB PDF) [file pgen.1000725.s006.pdf]

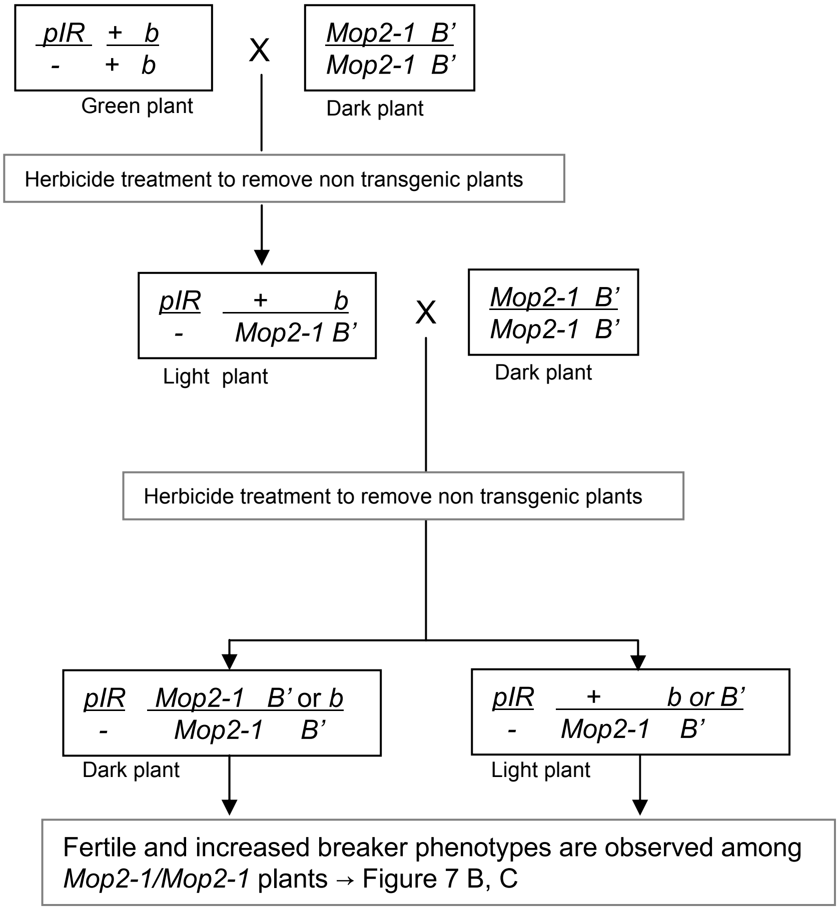

Figure S6. Schematic drawing of genetic experiment to whether *Mop2-1* prevents on Ubi::MS45pIR and Ubi::5126pIR transgene induced silencing. pIR is used to symbolize the inverted repeat transgenes. Dark plant pigmentation specified by *B'* in *Mop2-1/Mop2-1* plants was used to classify progeny. Molecular genotyping of a subset of plants revealed close correspondence between the *B'* phenotype and the *Mop2-1* genotype; 28/33 dark plants were homozygous and 28/30 light plants were heterozygous for *Mop2-1*. Recombination between the linked *b1* and *mop2* loci does not influence outcome of this experiment because at least one *B'* allele is present to report the *Mop2-1* genotype.
