# Supplementary material for: A Dominant Mutation in mediator of paramutation2, One of Three Second-Largest Subunits of a Plant-Specific RNA Polymerase, Disrupts Multiple siRNA Silencing Processes
Source: PLoS Genet. 2009 Nov 20;5(11):e1000725. doi: 10.1371/journal.pgen.1000725 (PMC2774164; doi:10.1371/journal.pgen.1000725)
Supplement: Figure S7 — Protein models of maize second largest subunits of Pol-I and Pol-II used for phylogenetic analysis. Protein sequences of the maize second largest subunits of Pol-I (ZmNRPA2) and Pol-II (ZmNRPB2a, ZmNRPB2b) were predicted using FGENESH+ (http://linux1.softberry.com/) software and the corresponding Arabidopsis proteins as guides. (0.04 MB DOC) [file pgen.1000725.s007.doc]

**Figure S7. Protein Models of Maize Second Largest Subunits of Pol-I and Pol-II Used for Phylogenetic Analysis.** Protein sequences of the maize second largest subunits of Pol-I *(ZmNRPA2)* and Pol-II (*ZmNRPB2a*, *ZmNRPB2b)* were predicted using FGENESH+ (<http://linux1.softberry.com/>) software and the corresponding Arabidopsis proteins as guides.

>ZmNRPA2; putative second largest subunit of maize RNA polymerase I similar to AtNRPA2 NP_564341.2; predicted from AC195164.3 Zea mays chromosome 1 clone ZMMBBb-332C21; ZMMBBb0332C21 MGKANKSPSPAVAKEGDYSALRELFRPHVESFDYFLDKGLDEMIESIRPMVIRDPNSSNT

LKNILHASNSFFQCRQARISYHGEFKVEVCFQYNEGAQIRHTFNFGHLPIMLMSKLCHLR

GADPHKLVFHGEEATEMGGYFICGGMERLVRILVLQKRNYPMGLVRNSFLKRGAGYTDKA

VVIRCVQRDQSSVTIKLYYILNGSARLGFWLGGREFLLPVGIVLKALIDASDREIFTSLT

CCYSDKHGRGKGVVSTQLIGERAQIVLDEVRNLSILTRTQCLVHIAVLKDYIFVHLENNH

DKFNLLIFMLQKLYALVDQTASPDNPDALQYQEALLPGHLFTVFLKDRLQEWLRKSKRLI

LEEAAKNKALEIRKFLTKYTTSVGRAVESMIKVGNVNSRSGLDLPQREGMTIHAERLNFH

RYISHFRSVHRGSAFAKMRTTSVRKLLPESWGFLCPVHTPDGEPCGLLNHMTSTCRVSSF

YNSEGVIKNFGEMKKLLSAELVRVGMNPVLPKIEQTGPPEVLHVHLDGCILGTIASAMIE

KAVNYLRTLKLLAHSGIPEDLEVGYVPPSFNGAFPGLYLFTNPARFVRPVRNLFILSDGK

QSIELIGPFEQAFMEIRCPDGGDGGRQEPFPATHEEIHPTAILSVVANLTPWSDHNQSPR

NMYQCQMAKQTMGFCGQALKFRTDVKAFHLQTPQTPIVRTGTYTKYCMDEFPSGTNAIVA

VLSYTGYDMEDAMILNKSAVERGMFRGHIYQASIVSLIIYYGLPRLGQAVHPNEQYYSVH

NSLTNVIRPVKLKGTEPAFIDYVAVNGTGSKCGLQKANIRLRRVRNPIIGDKFSSRHGQK

GVCSQLWPDIDMPFSANTGMRPDLIINPHAFPSRMTIAMLLESMAAKAGSLHGKFIDATP

FANSLKKDKDSDKPNSIVDELGPMLASYGFNYHGNEVLYSGVFGTEMDCEIFIGPVYYQR

LRHMVSDKFQVRTTGRIDQITRQPIGGRKHGGGIRFGEMERDALLAHGSSYLLHDRLHSC

SDYHIADVCSFCGSLLTATMIKSDTQKKVKHEMLGLPTVRPAKNFACQACKTSKGMETVA

MPYVFRYLAAELAAMNIKLELRLSNKSGLPLS

>ZmNRPB2a; putative second largest subunit of maize RNA polymerase II similar to AtNRPD2 CAA79528.1; predicted from AC194338.3; Zea mays chromosome 5 clone ZMMBBb-420P11; ZMMBBb0420P11

MEDDEYEDGMDMGYGGHHQRGGGHAGYGAEEDDEMGYGEGGGDGDEMEEEADGDAEQQED

ITQDDAWAVISAYFEEKGLVRQQLDSFDEFIQNTMQEIVDESADIEIRPESQHNPGRQAE

FAETLHKISFGQIYLSKPMMTEADGETATLFPKSARLRNLTYSAPLYVDVSYRVMKKGHD

CEEVTETAEYPKVFIGKVPIMLRSSYCTLYQQSEKDLTELGECPYDQGGYFIINGSEKVL

IAQEKMSTNHVYVFKKRQPNKFAYVAEVRSMAENQNRPASSMFVRMLSRAGAKGGSSGQY

IRATLPYIRADIPIIIVFRALGFVADKDILEHICYDFSDTQMMELLRPSLEEAFVIQNQQ

VALDYIGKRGATVGVTREKRIKYAKEILQKEMLPHVGVGEFCETKKAYYFGYIIHRLLMC

ALSRRAEDDRDHYGNKRLDLAGPLLGGLFRMLFRKLTRDVRSYVQKCVDNGKEVNLQFAI

KAKTITSGLKYSLATGNWGQANQAGTRAGVSQVLNRLTYASTLSHLRRLNSPIGREGKLA

KPRQLHNSHWGMMCPAETPEGQACGLVKNLALMVYITVGSAANPILEFLEEWGTENFEEI

SPAVIPQAAKIFVNGCWVGIHRNPDLLVKTLRRLRRQIDVNTEVGVVRDIRLKELRLYTD

YGRCSRPLFIVEGQRLLIKKAHIRALQQRETPDEGWHELVSKGYIEYIDTEEEETTMISM

TINDLQNARHNPEEAYSETYTHCEIHPSLILGVCASIIPFPDHNQSPRNTYQSAMGKQAM

GIYVTNYQLRMDTLAYVLYYPQKPLVTTRAMEHLHFRQLPAGINAIVAIACYSGYNQEDS

VIMNQSSIDRGFFRSLFFRSYRDEEKKMGTLIKEEFGRPNRENTMGMRHGSYDKLDDDGL

APPGTRVSGEDVIIGKTSPIPQDDAQGQASRYSKRDHSTALRHSESGMVDQVLLTTNADG

LRFVKVRMRSVRIPQIGDKFSSRHGQKGTVGMTYTQEDMPWTIEGITPDIIVNPHAIPSR

MTIGQLIECIMGKVAAQMGKEGDATPFTDVTVDNISKALHKCNYQMRGFETMYNGHTGRK

LTAMIFLGPTYYQRLKHMVDDKIHSRGRGPVQILTRQPAEGRSRDGGLRFGEMERDCMIA

HGAAFFLKERLFDQSDAYRVHVCEKCGLIAIANLKKNSFECRGCKNKTDIVQVHIPYACK

LLFQELMAMAIAPRMLTHDMKTGKDQKKR

>ZmNRPB2b partial; putative second largest subunit of maize RNA polymerase II similar to AtNRPD2 CAA79528.1; predicted from AC213890.4 Zea mays chromosome 1 clone CH201-88N18; ZMMBBc0088N18, MEDDEYEEGMEMGYGGHHQHGGGHAGYGAEEDDEVGYGGGGGEEMDEDADGDAEQQEDIT

QDDAWAVISAYFEEKGLVRQQLDSFDEFIQNTMQEIVDESADIEIRPESQHNPGRQAEFA

ETLHRISFGQIYLSKPMMTEADGETATLFPKSARLRNLTYSAPLYVDVSYRVMKKGHDCE

EVTETAEYPKVFIGKVPIMLRSSYCTLYQQSEKDLTELGECPYDQGGYFIINGSEKVLIA

QEKMSTNHVYVFKKRQPNKFAYVAEVRSMAENQNRPASGMFVRMLSRAGAKGGSSGQYIR

ATLPYIRADIPIIIVFRALGFVADKDILEHICYDFSDTQMMELLRPSLEEAFVIQNQQVA

LDYIGKRGATVGVTREKRIKYAKEILQKEMLPHVGVGEFCETKKAYYFGYIIHRLLMCAL

SRRAEDDRDHYGNKRLDLAGPLLGGLFRMLFRKLTRDVRSYVQKCVDNGKEVNLQFAIKA

KTVTSGLKYSLATGNWGQANQAGTRAGVSQ_gap_of_unknown_length_GKLAKPRQLHNSHWGMMCPAETPEGQACGLVKNLALMVYITVGSAANPILEFLEEWGTENFEEISPAVIPQAAKIFVNGCWVGIHRNPDLLVKTLRRLRRQIDVNTEVGVVRDIRLKELRLYTDYGRCSRPLFIVEGQRLLIKKAHIRALQQRETPDEGWHELVSKGYIEYIDTEEEETTMISMTINDLQNARHNPEEAYSETYTHCEIHPSLILGVCASIIPFPDHNQSPRNTYQSAMGKQAMGIYVTNYQLRMDTLAYVLYYPQKPLVTTRAMEHLHFRQLPAGINAIVAIACYSGYNQEDSVIMNQSSIDRGFFRSLFFRSYRDEEKKMGTLVKEEFGRPNRENTMGMRHGSYDKLDDDGLAPPGTRVSGEDVIIGKTSPIPQDDAQGQASRYSKRDHSTSLRHSESGMVDQVLLTTNADGLRFVKVRMRSVRIPQIGDKFSSRHGQKGTVGMTYTQEDMPWTIEGITPDIIVNPHAIPSRMTIGQLIECIMGKVAAQMGKEGDATPFTDVTVDNISKALHKCNYQMRGFETMYNGHTGRKLTAMIFLGPTYYQRLKHMVDDKIHSRGRGPVQILTRQPAEGRSRDGGLRFGEMERDCMIAHGAAFFLKERLFDQSDAYRVHVCEKCGLIAIANLKKNSFECRGCKNKTDIVQVHIPYACKLLFQELMAMAIAPRMLTHDMKTGKDQKKR
