# Supplementary material for: A Dominant Mutation in mediator of paramutation2, One of Three Second-Largest Subunits of a Plant-Specific RNA Polymerase, Disrupts Multiple siRNA Silencing Processes
Source: PLoS Genet. 2009 Nov 20;5(11):e1000725. doi: 10.1371/journal.pgen.1000725 (PMC2774164; doi:10.1371/journal.pgen.1000725)
Supplement: Table S1 — Information for DNA dependent RNA polymerase second-largest subunits used for phylogenetic analysis. (0.02 MB PDF) [file pgen.1000725.s008.pdf]

Table S1. Information for DNA Dependent RNA Polymerase Second Largest Subunits Used for Phylogenetic Analysis

Accession	Abbreviation	Species
Pol I		
M62804	ScRPA2	*Saccharomyces cerevisiae*
AAF51503	DmRPA2	*Drosophila melanogaster*
Q9H9Y6	HsRPA2	*Homo sapiens*
NP_922143	OsNRPA2	*Oryza sativa*
NM_102734	AtNRPA2	*Arabidopsis thaliana*
XP_002297732.1	PtNRPA2	*Populus trichocarpa*
Figure S7	ZmNRPA2	*Zea mays*
EDQ61303.1	PpNRPA2	*Physcomitrella patens subsp. Patens*
Pol II		
EDQ68753.1	PpNRPB2	*Physcomitrella patens subsp. patens*
NM_118291	AtNRPB2	*Arabidopsis thaliana*
P08266	DmRPB2	*Drosophila melanogaster*
P08518	ScRPB2	*Saccharomyces cerevisiae*
AAH23503	HsRPB2	*Homo sapiens*
AAC49273.1	SlNRPB2	*Solanum lycopersicum*
Figure S7	ZmNRPB2a	*Zea mays*
Figure S7	ZmNRPB2b	*Zea mays*
ABF97884.1	OsNRPB2	*Oryza sativa*
Pol III		
XP_002321356.1	PtNRPC2	*Populus trichocarpa*
EDQ79517.1	PpNRPC2	*Physcomitrella patens subsp. patens*
NM_123882	AtNRPC2	*Arabidopsis thaliana*
AAB59324	ScNRPC2	*Saccharomyces cerevisiae*
CAA35185	DmRPC2	*Drosophila melanogaster*
AAM18214	HsRPC2	*Homo sapiens*
XP_470900	OsNRPC2	*Oryza sativa*
Pol IV		
XP_002324332.1	PtNRPD2	*Populus trichocarpa*
NM_113282	AtNRPD2/E2a	*Arabidopsis thaliana* (At3g23780)
NM_112691	AtNRPD2/E2b	*Arabidopsis thaliana* (At3g18090)
AK121416	OsNRPD2a	*Oryza sativa*
XM_480298	OsNRPD2b	*Oryza sativa*
GQ453405	ZmNRPD2/E2a	*Zea mays*
GRMZM2G146935_T02	ZmNRPD2/E2b	*Zea mays* (model from www.maizesequence.org)
GRMZM2G133512_T01	ZmNRPD2/E2c	*Zea mays* (model from www.maizesequence.org)
AAY68206.1	SlNRPD2	*Solanum lycopersicum*
Moss		
EDQ56228.1	PpNRPD2a	*Physcomitrella patens subsp. patens*
EDQ80791.1	PpNRPD2b	*Physcomitrella patens subsp. Patens*
Achaeae		
CAA47722	Tc	*Thermococcus celer*
NP_148216.1	Ap	*Aeropyrum pernix K1*
CAA32924	Sa	*Sulfolobus acidocaldarius*
Virus		
CAE52727	FPV	*Fowlpox virus*
AAC55257	MCV	*Molluscum contagiosum virus*
AAF15002	MV	*Myxoma virus*
AAO89423	VV	*Vaccinia virus*
AAL40593	MPV	*Monkeypox virus*

Bacteria		
NP_807130.1	SeRpoB	*Salmonella enterica*
NP_252960.1	PaRpoB	*Pseudomonas aeruginosa*
NP_312937.1	EcRpoB	*Escherichia coli*
Chloroplast and Cyanobacteria		
BAA57969	CvCPST	*Chlorella vulgaris (green algae)*
CAA50138	EgCPST	*Euglena gracilis*
NP_045031	CcCPST	*Cyanidium caldarium*
NP_043230	CpCPST	*Cyanophora paradoxa*
Q9TL06	NoCPST	*Nephroselmis olivacea*
BAA84377	AtCPST	*Arabidopsis thaliana*
CAA60276	ZmCPST	*Zea mays*
P11703	SoCPST	*Spinacia oleracea*
NP_039373	OsCPST	*Oryza sativa*
P06271	NtCPST	*Nicotiana tabacum*
